# Probing the Binding Requirements of Modified Nucleosides with the DNA Nuclease SNM1A

**DOI:** 10.3390/molecules26020320

**Published:** 2021-01-09

**Authors:** Eva-Maria Dürr, Joanna F. McGouran

**Affiliations:** School of Chemistry and Trinity Biomedical Sciences Institute, Trinity College Dublin, 152-160 Pearse St., D02 R590 Dublin, Ireland; durre@tcd.ie

**Keywords:** nuclease, interstrand crosslink repair, SNM1A, nucleoside inhibitor, malonate, hydroxamic acid

## Abstract

SNM1A is a nuclease that is implicated in DNA interstrand crosslink repair and, as such, its inhibition is of interest for overcoming resistance to chemotherapeutic crosslinking agents. However, the number and identity of the metal ion(s) in the active site of SNM1A are still unconfirmed, and only a limited number of inhibitors have been reported to date. Herein, we report the synthesis and evaluation of a family of malonate-based modified nucleosides to investigate the optimal positioning of metal-binding groups in nucleoside-derived inhibitors for SNM1A. These compounds include ester, carboxylate and hydroxamic acid malonate derivatives which were installed in the 5′-position or 3′-position of thymidine or as a linkage between two nucleosides. Evaluation as inhibitors of recombinant SNM1A showed that nine of the twelve compounds tested had an inhibitory effect at 1 mM concentration. The most potent compound contains a hydroxamic acid malonate group at the 5′-position. Overall, our studies advance the understanding of requirements for nucleoside-derived inhibitors for SNM1A and indicate that groups containing a negatively charged group in close proximity to a metal chelator, such as hydroxamic acid malonates, are promising structures in the design of inhibitors.

## 1. Introduction

The nuclease SNM1A (also known as DCLRE1A) is part of the interstrand crosslink repair machinery in mammalian cells [1]. Depletion of SNM1A increases sensitivity to crosslinking agents [2,3,4,5], although the precise mechanisms behind this are still under investigation. Current research suggests that SNM1A digests past crosslinks from an incision 5′ to the site of the lesion; this role and other proposed pathways are summarised in a recent review [1]. SNM1A has been reported to have 5′-exonuclease as well as endonuclease activity, although endonuclease activity requires a blocked 5′-end and stoichiometric amounts of enzyme [6]. Exonuclease activity requires a 5′-phosphate group and is processive for high molecular weight substrates [7]. The active site of SNM1A is postulated to contain a di-metal centre, based on similarity with other members of the β-CASP family [8]. However, only one metal ion has been observed in crystal structures of SNM1A to date [9]. The active site is located within a wide binding groove, which permits the processing of bulky substrates such as DNA containing interstrand crosslinks [8].

**Figure 1:**
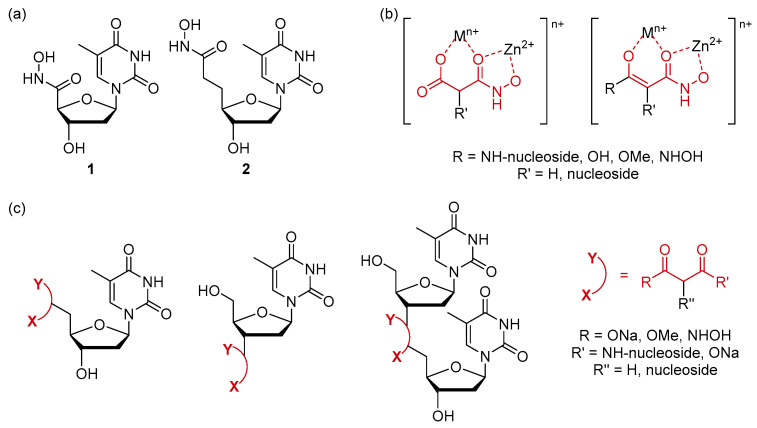
Design of modified nucleosides designed to bind to SNM1A. (**a**) Previously reported nucleoside inhibitors of SNM1A; (**b**) the proposed binding mode for hydroxamic acid malonate derivatives; (**c**) structures of target (di)nucleosides.

Like other DNA damage repair proteins [10], SNM1A is a potential target for overcoming resistance to crosslinking agents in cancers [11]. Inhibitors reported to date are limited to *o*-phenanthroline [12], cephalosporins [13], and a modified nucleoside inhibitor [14]. In our previous work designing nucleoside inhibitors, the hydroxamic acid modification emerged as the most promising group [14]. Nucleosides **1** and **2** (Figure 1a) were found to inhibit SNM1A to varying degrees, and the more potent compound **1** was found to have an IC_50_ value of 139 µM. However, both compounds likely only target one of the two postulated metal ions in the active site of SNM1A. Therefore, in this work, we expand the metal-binding group to a malonate group to probe whether an extended metal-binding group achieves chelation to both postulated metal ions.

In recent crystal structures of SNM1A, malonate was observed bound to the metal ion in the active site [9]. This highlights the suitability of the group as a metal chelator. The malonate group has found application both as a scaffold for a hydroxamic acid group [15,16,17,18,19,20,21,22] and as a phosphate bioisostere in many different contexts, including analogues of phosphosugars [23,24,25,26] and nucleotides [27,28]. By combining the malonate group with the hydroxamic acid group, binding of both metal ions is envisaged (Figure 1b). The substituents of the malonate group were varied to include esters and carboxylate salts to obtain further information about the binding mode.

Derivatives utilised either one carboxylic acid group of the malonate group to connect the group to the nucleoside via an amide linkage (*N*-linked), or alkylation at the α-carbon (*C*-linked) was employed to install the malonate group onto the nucleoside. The nucleobase used for this family of modified nucleosides is thymine, allowing for direct comparison with the previously evaluated compounds [14]. The 5′-*C*-linked series in particular is directly related to the previously reported compound **2**, as its malonate group is at the same distance from the deoxyribose core as the hydroxamic acid group in compound **2**. However, the malonate modifications were introduced not only at the 5′-position, but also at the 3′-position and as a linker between two nucleosides (Figure 1c) to probe the interactions between the nucleoside and the active site of recombinant SNM1A in gel-based assays.

## 2. Results

### 2.1. Synthesis

**Scheme 1:**
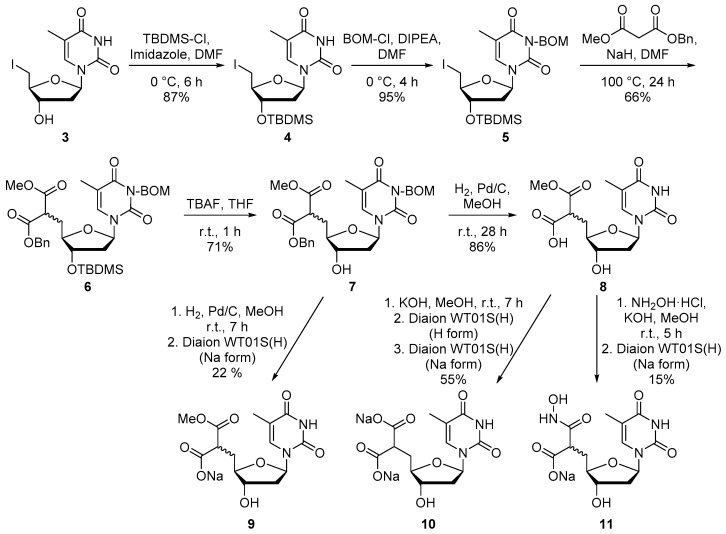
Synthesis of 5′-modified *C*-linked targets **9**, **10** and **11**.

The synthesis of the 5′-alkylated targets was adapted from Zlatev et al. [28] and is shown in Scheme 1. Starting from 5′-deoxy-5′-iodothymidine (**3**), protection of the 3′-hydroxy group as the silyl ether and of the nucleobase with the benzyloxymethyl group was carried out in 87% and 95% yield, respectively. This protected iodide **5** then underwent nucleophilic substitution with benzyl methyl malonate in 66% yield. Removal of the silyl ether of malonate ester **6** afforded alcohol **7** in 71% yield, and catalytic hydrogenation removed both the benzyloxymethyl group and the benzyl group to give carboxylic acid **8** in 86% yield. For use in assays, the sodium carboxylate salt **9** was required, so ion exchange was carried out following hydrogenation. Due to contamination with ion-exchange resin, purification by reversed-phase preparative TLC was employed and gave target **9** in a low yield of 22%. Carboxylic acid **8** was used as an intermediate for targets **10** and **11**. Following ester hydrolysis and ion exchange to furnish carboxylate salt **10**, reversed-phase preparative TLC was again used to purify compound **10**, which was obtained in 55% yield. Carboxylic acid **8** was also employed in the synthesis of hydroxamic acid **11**, which was obtained in 15% yield via aminolysis with hydroxylamine [29]. Targets **9** and **11** were isolated and used as mixtures of interconverting diastereomers. Unfortunately, synthesis of a 1,3-dihydroxamic acid is not possible using aminolysis with hydroxylamine, as the treatment of a 1,3-diester with hydroxylamine under basic conditions results in cyclisation to form isoxazolidine-3,5-diones [30,31].

**Scheme 2:**
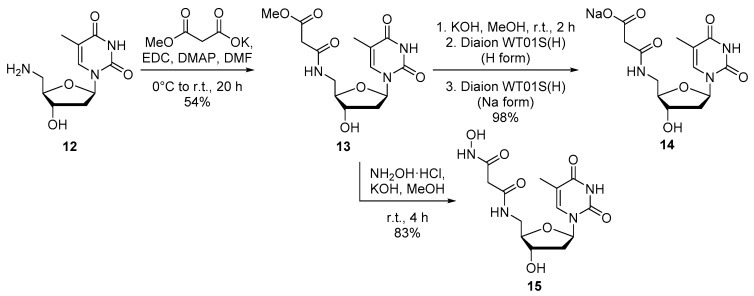
Synthesis of 5′-modified *N*-linked targets **13**, **14** and **15**.

For the *N*-linked targets, amide coupling between 5′-amino-5′-deoxythymidine (**12**) and monomethyl potassium malonate was employed (Scheme 2) to furnish ester **13** in 54% yield. This target served as an intermediate in the synthesis of targets **14** and **15**. Ester hydrolysis followed by ion exchange afforded sodium carboxylate **14** in 98% yield. Hydroxamic acid **15** was obtained in 83% yield by treating ester **13** with hydroxylamine under basic conditions [29].

**Scheme 3:**
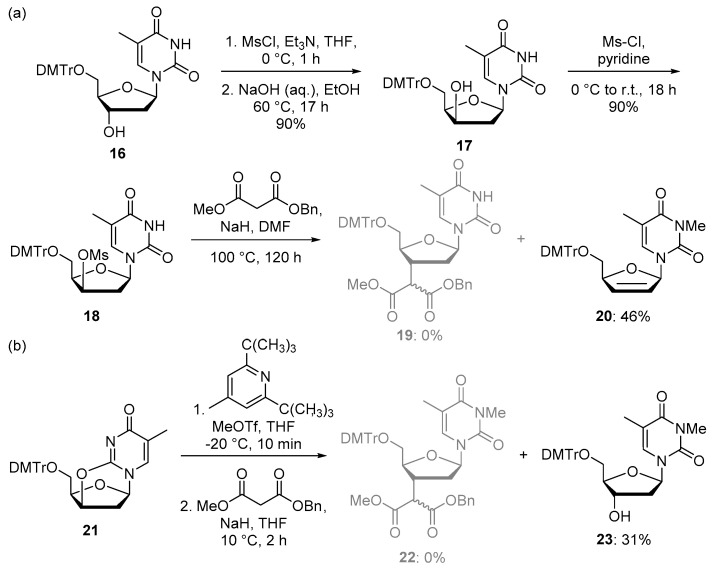
Attempted syntheses of 3′-modified targets. (**a**) Attempted synthesis of *C*-linked targets from mesylate **18**. (**b**) Attempted synthesis of *C*-linked targets from protected anhydrothymidine **21**.

Synthesis of the 3′-modified targets was attempted analogous to the 5′-modified compounds. For the *C*-linked targets, initially nucleophilic substitution of a mesylate was envisaged (Scheme 3a). Inversion of the 3′-hydroxy group of protected thymidine **16** was carried out by sequential mesylation and hydrolysis [32] in 90% yield. Mesylation [33] of the alcohol **17** proceeded in 90% yield. However, substitution of this mesylate **18** with benzyl methyl malonate was unsuccessful, with the *N*-methylated elimination product **20** obtained in 46% yield instead of the desired product **19**. An alternative strategy to functionalise the 3′-position using protected 2,3′-anhydrothymidine [34] **21** and methyl triflate as an activating agent as reported by Saha et al. [35] was unsuccessful in our hands. Only the hydrolysed product **23** was obtained after quenching the reaction, with none of the desired product **22** observed (Scheme 3b).

**Scheme 4:**
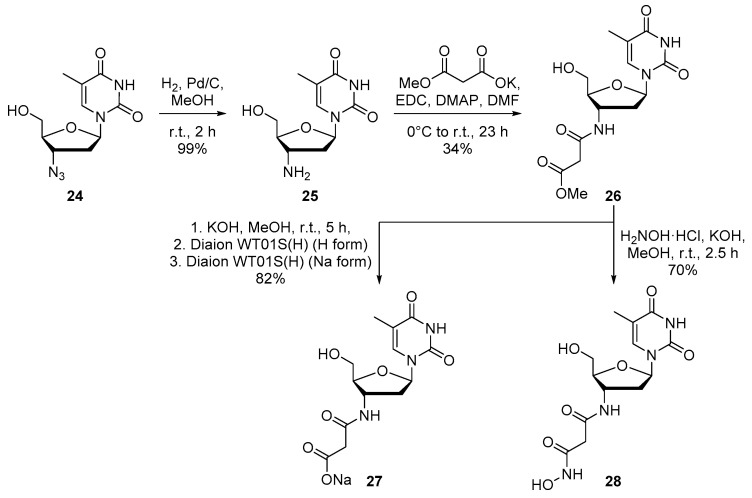
Synthesis of 3′-modified *N*-linked targets **26**–**28**.

We then turned our attention to the *N*-linked targets. Commercially available AZT (**24**) was reduced to aminothymidine (**25**) by catalytic hydrogenation in 99% yield (Scheme 4). Amine **25** was then used in an EDC-mediated amide coupling with monomethyl potassium malonate, which afforded target **26** in 34% yield. Treatment of ester **26** with KOH followed by ion exchange produced carboxylate salt **27** in 82% yield, while aminolysis of this ester using the same conditions as for the 5′-modified nucleosides afforded hydroxamic acid **28** in 70% yield.

**Scheme 5:**
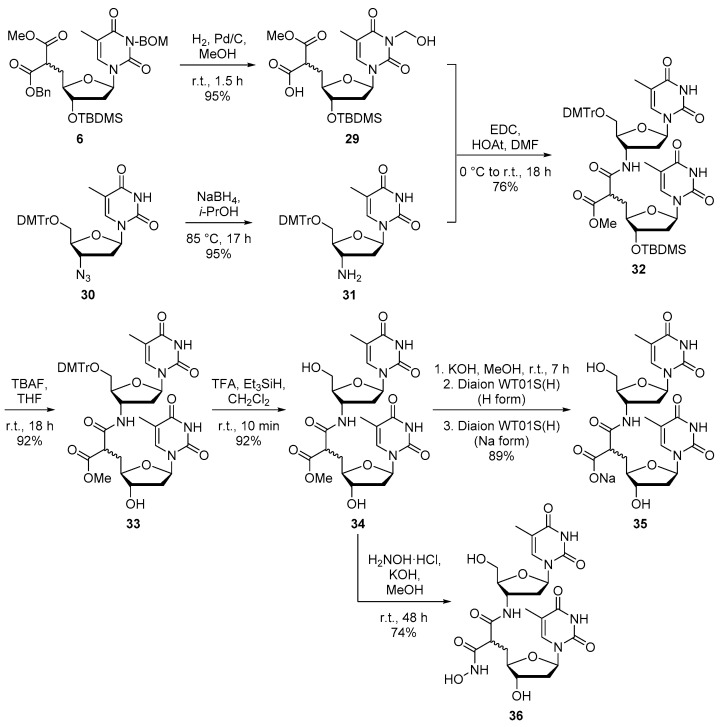
Synthesis of malonate-linked dinucleosides **34**–**36**.

For the final group of compounds, the malonate group forms a phosphodiester replacement and links two nucleosides. To this end, a 5′-modified carboxylic acid and a 3′-amino nucleoside were combined (Scheme 5). For the synthesis of the dinucleosides, protecting groups for the 5′- and 3′-hydroxy groups were used to aid solubility. The carboxylic acid nucleoside **29** was obtained from protected malonate ester **6** by catalytic hydrogenation. The benzyloxymethyl group of **6** was only partially removed in this step, but this hemiaminal did not interfere with further reactions. The other nucleoside, 5′-protected 3′-aminothymidine **31**, was synthesised from DMTr-protected AZT **30** [36]. Reduction of azide **30** furnished amine **31** in 95% yield. EDC-mediated coupling between nucleosides **29** and **31** afforded the fully protected dinucleoside **32** in 76% yield. Deprotection of the 3′- and 5′-hydroxy groups was achieved using TBAF and TFA, respectively, in 92% yield for each step. This sequence afforded target **34**, which was further derivatised. Sodium carboxylate **35** was furnished from ester **34** in 89% yield by ester hydrolysis and subsequent ion exchange, and hydroxamic acid **36** was obtained in 74% yield from ester **34** by aminolysis. As observed with the 5′-modified *C*-linked compounds **9** and **11**, the dinucleosides **34**–**36** were also isolated and used as mixtures of interconverting diastereomers.

The successful synthesis of the dinucleosides **34**–**36** completes a series of twelve nucleosides. These can be divided into four groups: 5′-modified *C*-linked, 5′-modified *N*-linked, 3′-modified *N*-linked, and dinucleosides. Each group consists of three compounds each, with an ester, sodium carboxylate and a hydroxamic acid analogue.

### 2.2. Biological Evaluation

**Figure 2:**
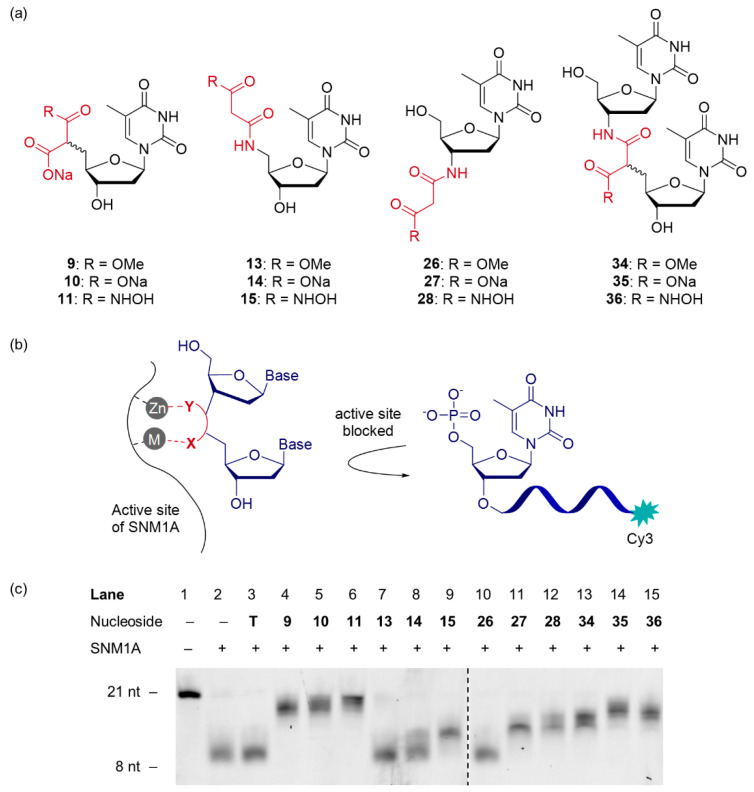
Evaluation of modified (di)nucleosides as inhibitors of SNM1A. (**a**) Structures of the twelve (di)nucleosides that were evaluated as inhibitors of SNM1A; (**b**) schematic representation of assay; (**c**) gel electrophoresis showing the extent of digestion of fluorescent 21-mer oligonucleotide substrate. SNM1A (2.5 nM) was pre-incubated with the modified (di)nucleosides (1 mM) for 5 min before the oligonucleotide substrate (80 nM) was added and a further 60 min incubation. nt = nucleotides.

The twelve modified nucleosides (Figure 2a) were evaluated as inhibitors of recombinant SNM1A to evaluate binding to the active site, using a previously reported gel-based assay with the same 21-mer oligonucleotide substrate [14]. The nucleosides were incubated with SNM1A for 5 min prior to the addition of the fluorescently tagged 21-mer oligonucleotide substrate and further incubation for 1 h. In the event of the nucleoside binding to the active site, digestion of the substrate proceeds less efficiently (Figure 2b). SNM1A removes nucleotide monomers from the 5′-end, generating a shorter oligonucleotide that in turn is a substrate for SNM1A and can be digested further. The size of the oligonucleotide products, analysed by gel electrophoresis, therefore indicates how strongly a compound binds to the nuclease.

The results of this assay for the twelve modified nucleosides are shown in Figure 2c. Lane 1 shows the oligonucleotide substrate in the absence of SNM1A, while lane 2 shows maximal digestion in 60 min, in the absence of any modified nucleoside. Thymidine (**T**) was used as a negative control to ensure that any inhibitory effects in lanes 4–15 can be attributed to the nucleoside modification, as thymidine has no effect on the hydrolysis of the substrate (Figure 2c, lane 3). Encouragingly, nine out of twelve malonate-based nucleosides showed some inhibition at 1 mM concentration (Figure 2c). Only three nucleosides (Figure 2c, lanes 7, 8, 10) had no impact on the activity of SNM1A.

Analysis of the size of oligonucleotide products remaining after the incubation for each compound family revealed the following: the 5′-*C*-linked series produced three compounds that led to almost full inhibition at 1 mM concentration, with malonate hydroxamic acid **11** emerging as the most potent overall (Figure 2c, lane 6). The dinucleoside series also afforded three active compounds that impeded SNM1A activity (Figure 2c, lanes 13–15) and was only slightly less effective in this assay than the 5′-*C*-linked series (Figure 2c, lanes 4–6). In contrast, both the 3′- and 5′-*N*-linked series contain compounds that did not have any effect on SNM1A, with only nucleosides **13**, **14** and **26** slowing the rate of hydrolysis (Figure 2c, lanes 9, 11, 12). Within each series, the hydroxamic acid modification was found to lead to the strongest inhibition (Figure 2c, lanes 6, 9, 12, 15), with the exception of the dinucleosides, where carboxylate **35** and hydroxamic acid **36** appear to have approximately equal potency (Figure 2c, lanes 14 vs. 15). The carboxylate modification resulted in inhibition in all families except the 5′-*N*-linked series (Figure 2c, lanes 5, 11, 14). The methyl ester analogues were only active for the 5′-*C*-linked series, which contains an additional carboxylate group, and the dinucleoside series (Figure 2c, lanes 4, 13).

**Figure 3:**
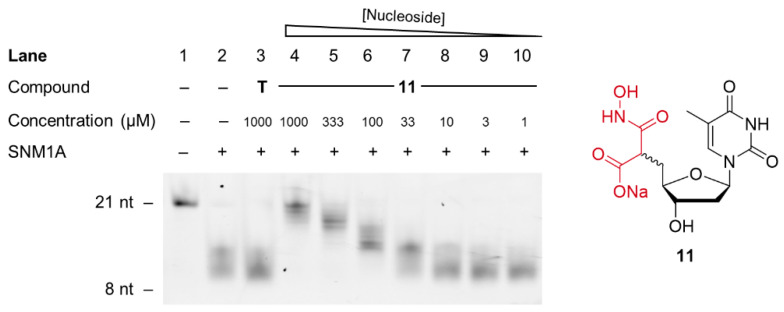
Concentration dependence of 5′-C-linked malonate nucleoside **11**. Digestion of the phosphorylated oligonucleotide (80 nM) after incubation with SNM1A (2.5 nM) and nucleoside **11** (1000–1 µM) or thymidine (**T**) (1 mM) for 60 min at 37 °C, analysed by denaturing PAGE. SNM1A was preincubated with the modified nucleoside for 5 min at 37 °C prior to the addition of the substrate. nt = nucleotides.

To further investigate the potency of the different nucleosides, concentration-dependence experiments were carried out for the nine nucleosides that showed inhibition (Figure 2 and Figures S1–S4), with concentrations ranging from 1 mM to 1 µM. Although partial digestion of the substrate by SNM1A was observed at concentrations below 1 mM, the strongest inhibitor **11** was found to impede hydrolysis at concentrations as low as 33 µM (Figure 3, lanes 4–7) and its activity was found to be concentration dependent.

**Figure 4:**
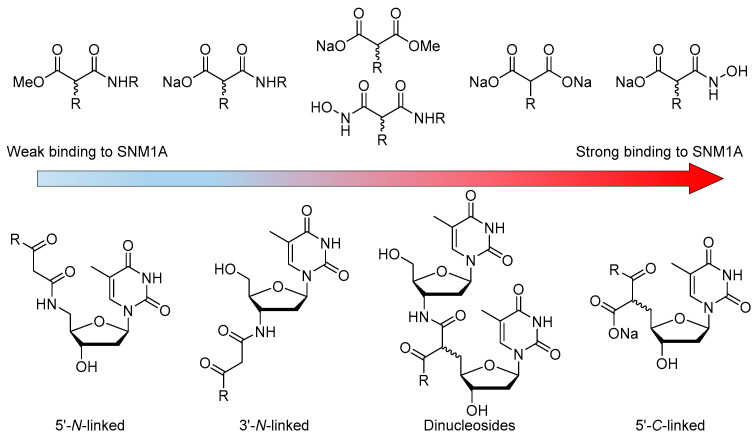
Trends observed from the analysis of modified (di)nucleosides in this work. Potency of the modifications increases in the following order: methyl ester malonamide < sodium carboxylate malonamide < hydroxamic acid malonamide ≈ monomethyl sodium malonate < disodium malonate < hydroxamic acid sodium malonate. For the site of modification, the observed trend is 5′-*N*-linked < 3′-*N*-linked < dinucleoside < 5′-*C*-linked.

The results of identical assays for the other modified nucleosides are summarised in Table 1 (for gel images see Figures S1–S4). All compounds that were active at 1 mM concentration also have a partial inhibitory effect at 333 µM, except dinucleoside **34** (Table 1, entry 10). Only the malonate hydroxamic acid **11** (Table 1, entry 3) was effective at lower concentrations. In three of the four series, the ester analogue is the weakest inhibitor (Table 1, entries 4, 7, 10; Figure 4). This result is consistent with the initial screen and can be explained by the lack of an acidic proton compared to hydroxamic acids or the lack of negatively charged atoms compared to carboxylate salts, making chelation unfavourable. This implies that the malonate group is not coordinating exclusively through the carbonyl oxygen atoms but requires substituents with the potential to hold a negative charge to achieve effective chelation. The 5′-*C*-linked malonate series (Table 1, entries 1–3) resulted in the strongest binding. This finding is not surprising given the additional carboxylate group that is present compared to all other derivatives. It is therefore likely that this carboxylate participates in coordination to the metal centre. As observed in Figure 2c and Table 1, the most potent compound is the hydroxamic acid malonate **11** (Table 1, entry 3).

In contrast to the *C*-linked malonates, of the *N*-linked 5′-modified nucleosides, only one compound (**15**) binds SNM1A. Its lower activity (Table 1, entry 6 vs. 3) compared to the corresponding *C*-linked hydroxamic acid **11** is possibly due to the increased distance of the hydroxamic acid group from the deoxyribose core in **15**, and/or the absence of the additional carboxylate group. However, the 3′-modified nucleosides **26**–**28** (Table 1, entries 7–9) also contain a malonamide group, and two compounds in this series inhibited SNM1A at 333 µM concentration (Table 1, entries, 8, 9). This implies that the relative positioning of the group compared to the deoxyribose core is a causative factor in the lack of potency of 5′-*N*-linked nucleosides (Figure 4). For dinucleosides, the lowest concentrations at which inhibition was observed for dinucleosides **35** and **36** are the same as for the 3′-modified analogues (Table 1, entries 8 vs. 11, 9 vs. 12). However, the ester derivative **34** showed some inhibition at 1 mM concentration (Table 1, entry 10), while the corresponding 3′-derivative **26** was inactive (Table 1, entry 7). These results point towards the importance of the second nucleoside as a recognition element. Nonetheless, the potency of the strongest dinucleosides was significantly lower than that of the strongest 5′-modified nucleoside **11** (Table 1, entry 3) which contained an additional carboxylate group. The benefits of a second nucleoside therefore do not counteract the negative effect of removing a carboxylate group. The trends observed in these gel-based assays are summarised in Figure 4.

**Figure 5:**
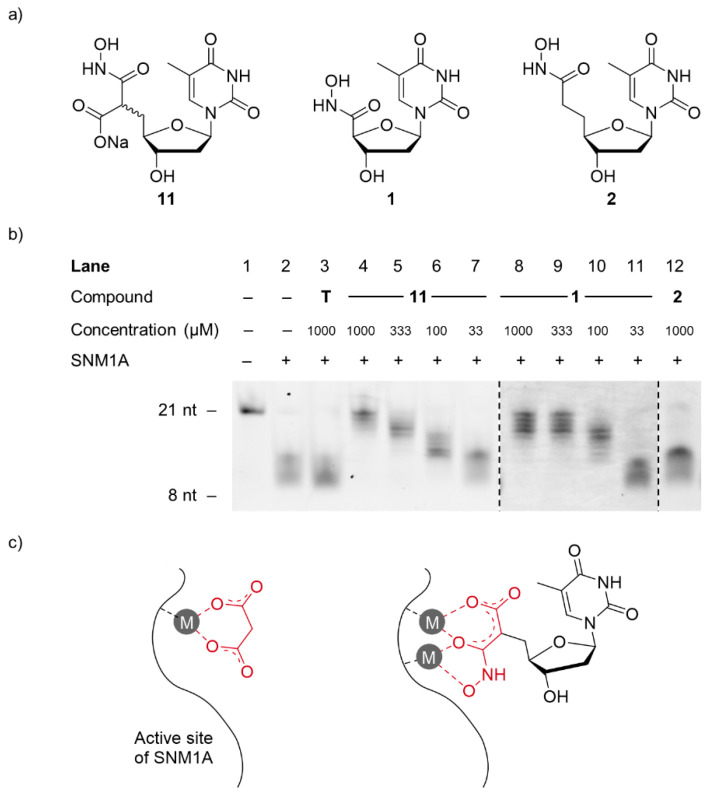
Comparison of previously reported compounds **1** and **2** and compound **11** and the proposed binding mode. (**a**) Structures of compounds **1**, **2** and **11**. (**b**) Inhibition of SNM1A by compounds **1**, **2** and **11** at various concentrations. (**c**) The observed binding mode of malonate anion in the active site of SNM1A and the proposed binding mode of compound **11**.

Comparison with the previously reported compounds **1** and **2** (Figure 5a,b) shows that compound **11** has a slightly lower potency than compound **1** (Figure 5b, lanes 4–7 vs. 8–11), which emerged as the best inhibitor in our previous work [14]. Structurally, compound **11** is an analogue of hydroxamic acid **2**, which differs only in the presence of an additional carboxylate group (Figure 5a). This additional group enhances binding significantly, as observed in Figure 5b (lane 4 vs. 12), which points towards interactions between the carboxylate group and the active site and supports the presence of a second metal ion. The binding mode of compound **11** to two metal ions shown in Figure 5c is supported by the different activities of nucleosides **2** and **11** and the observed binding mode of the malonate anion in crystal structures of SNM1A [9]. It is also consistent with the requirement for groups with the potential to hold a negative charge and the enhanced activity of the 5′-*C*-linked series, which contains an additional carboxylate group. It is, however, important to note that these trends could also be the result of other interactions such as hydrogen bonding with amino acid residues in the active site. The weaker binding of the carboxylate **10** compared to the hydroxamic acid **11** (Table 1, entries 2 vs. 3) can be attributed to the strong chelating ability of the hydroxamic acid group resulting from the formation of a five-membered ring [37], as shown in Figure 5c.

Overall, our studies show that an extended metal-chelating group, such as malonate hydroxamic acids, leads to enhanced binding to the nuclease SNM1A, consistent with the presence of two metal ions in the active site. The synthesis and evaluation of methyl ester, carboxylate and hydroxamic acid analogues of each point of installation revealed that a group with the potential to hold a negative charge is required for binding. Variation in the positioning of the malonate groups relative to the deoxyribose group revealed that of the *N*-linked compounds, modification of the 3′-position led to increased potency compared to installation at the 5′-position. Interestingly, malonamide-linked dinucleosides were found to be slightly superior to their 3′-modified analogues, which highlights the inclusion of two nucleosides as recognition elements as a promising avenue for future nucleoside-based inhibitors.

## 3. Materials and Methods

### 3.1. General

^1^H and ^13^C NMR spectra were recorded on Bruker 400 MHz or 600 MHz system spectrometers in DMSO-*d*_6_, CDCl_3_, CD_3_OD, acetone-*d*_6_ or D_2_O relative to residual DMSO (δ_H_ = 2.50 ppm, δ_C_ = 39.52 ppm), CDCl_3_, (δ_H_ = 7.26 ppm, δ_C_ = 77.16 ppm), CD_3_OD (δ_H_ = 3.31 ppm, δ_C_ = 49.00 ppm), acetone-*d*_6_ (δ_H_ = 2.05 ppm, δ_C_ = 29.84 ppm) or D_2_O (δ_H_ = 4.79 ppm) [38]. Chemical shifts are reported in ppm and coupling constants are reported in Hertz (Hz) and accurate to 0.2 Hz. ^13^C NMR spectra are proton decoupled. NMR spectra were assigned using HSQC, HMBC, DEPT and EXSY experiments. Modified nucleosides are numbered according to standard nucleoside conventions. Mass spectrometry measurements were carried out on a Bruker ESI or APCI HRMS. Melting points were measured using a Griffin melting point apparatus and are uncorrected. Infrared (IR) spectra were obtained on a Perkin Elmer spectrophotometer. Flash column chromatography was carried out using silica gel, particle size 0.04–0.063 mm, purchased from Sigma Aldrich or VWR. TLC analysis was performed on TLC Silica gel 60 F_254_ plates purchased from Merck and visualised by UV irradiation (254 nm), ninhydrin stain (1.5 g ninhydrin, 5 mL AcOH, 500 mL 95% EtOH), anisaldehyde stain (9.2 mL *p*-methoxybenzaldehyde, 3.75 mL AcOH, 338 mL 95% EtOH, 12.5 mL conc. H_2_SO_4_) and iodine. Ion-exchange resin refers to Diaion WT01S(H) resin, which was purchased from Alfa Aesar and activated by consecutive washes with acetone, MeOH, 1 M NaOH (Na form only), H_2_O and MeOH. Preparative reversed-phase TLC was carried out on TLC silica gel 60 RP-18 F_254_S plates purchased from Merck. THF and CH_2_Cl_2_ were dried using a PureSolv MD solvent purification system. Petroleum ether refers to the fraction of petroleum ether that boils at 40–60 °C. Chemicals were purchased from Acros Organics (Fair Lawn, NJ, US), Aldrich (Milwaukee, W, USA), Alfa Aesar(Haverhill, MA, USA), Carbosynth (Compton, UK), Fisher Scientific (Waltham, MA, USA), Fluorochem (Glossop, UK), Sigma Aldrich (St. Louis, MO, USA) and Merck (Kenilworth, NJ, USA) and were used as purchased without further purification.

### 3.2. Synthesis

**3′-*O*-(*tert*-Butyldimethylsilyl)-5′-deoxy-5′-iodothymidine (4):** Nucleoside **4** was prepared according to a modified procedure [39]. Iodide **3** (10.0 g, 28.4 mmol) was dissolved in dry DMF (20 mL) under argon and cooled to 0 °C. Imidazole (2.51 g, 36.9 mmol) and TBDMS-Cl (5.56 g, 36.9 mmol) were added and the reaction mixture was stirred at 0 °C for 6 h. After this time, TLC analysis (EtOAc) showed the consumption of starting material (R_f_ = 0.5) and the formation of the product (R_f_ = 0.8). The reaction was quenched by the addition of MeOH (5 mL), diluted with EtOAc (250 mL) and H_2_O (100 mL). The layers were separated and the organic layer was washed with brine (100 mL), dried over MgSO_4_, filtered and concentrated. The residue was recrystallised from EtOH to afford the desired product **4** as a white crystalline solid (11.59 g, 87%); mp 156–159 °C. ν_max_/cm^−1^ (neat) 3155 (NH), 3024 (CH), 2930 (CH), 2892 (CH), 2857 (CH), 1691 (C=O), 1655 (C=O), 1470 (CH), 1426, 1402, 1369, 1309, 1291, 1274, 1197, 1130, 1100 (C-O), 1051, 1039, 829, 813, 777. ^1^H NMR (400 MHz, CDCl_3_): δ = 0.11 (s, 3 H, CH_3_^TBDMS^), 0.12 (s, 3 H, CH_3_^TBDMS^), 0.90 (s, 9 H, *t*-Bu^TBDMS^), 1.95 (s, 3 H, CH_3_^T^), 2.15–2.22 (m, 1 H, H2′a), 2.29 (ddd, *J_2′b,3′_* = 3.9 Hz, *J_1′,2′b_* = 6.6 Hz, *J_2′a,2′b_* = 13.7 Hz, 1 H, H2′b), 3.39 (dd, *J_4′,5′a_* = 4.6 Hz, *J_5′a,5′b_* = 11.0 Hz, 1 H, H5′a), 3.47 (dd, *J_4′,5′b_* = 3.7 Hz, *J_5′a,5′b_* = 11.0 Hz, 1 H, H5′b), 3.67–3.70 (m, 1 H, H4′), 4.26 (app. dt, *J* = 3.9 Hz, *J* = 7.0 Hz, 1 H, H3′), 6.27 (app. t, *J* = 6.6 Hz, 1 H, H1′), 7.47 (s, 1 H, H6), 8.49 (bs, 1 H, NH) ppm. ^13^C NMR (100 MHz, CDCl_3_): δ = −4.5 (CH_3_^TBDMS^), −4.4 (CH_3_^TBDMS^), 7.3 (C5′), 12.8 (CH_3_^T^), 18.0 (qC, *t*-Bu^TBDMS^), 25.8 (*t*-Bu^TBDMS^), 40.6 (C2′), 75.2 (C3′), 84.3 (C4′), 84.8 (C1′), 111.4 (C5), 136.0 (C6), 150.1 (C2), 163.6 (C4) ppm. HRMS (ESI^−^): *m/z* calc. 465.0712 [M − H]^−^, found: 465.0704. The spectroscopic data are in agreement with those reported in the literature [39].

***N*-Benzyloxymethyl-3′-*O*-(*tert*-butyldimethylsilyl)-5′-deoxy-5′-iodothymidine (5):** Protected iodide **4** (9.33 g, 20.0 mmol) was dissolved in dry DMF (80 mL) under argon and cooled to 0 °C. DIPEA (17.5 mL, 97.6 mmol) was added, followed by the dropwise addition of benzyl chloromethyl ether (8.4 mL, 57.7 mmol). The reaction mixture was stirred at 0 °C for 4 h. After this time, TLC analysis (petroleum ether-EtOAc, 2:1) showed the consumption of starting material (R_f_ = 0.1) and the formation of the product (R_f_ = 0.4). The reaction was quenched by the addition of MeOH (10 mL) followed by H_2_O (100 mL). The mixture was extracted with Et_2_O (2 × 100 mL) and the combined organic extracts were dried over MgSO_4_, filtered, concentrated and coevaporated with toluene. Purification by flash column chromatography (EtOAc-petroleum ether, 3:1→2:1) afforded the desired product **5** as a yellow oil (1.16 g, 95%). ν_max_/cm^−1^ (neat) 2954 (CH), 2929 (CH), 2857 (CH), 1710 (C=O), 1655 (C=O), 1463 (CH), 1361, 1275, 1253, 1069 (C-O), 1030, 833, 773, 733, 697. ^1^H NMR (400 MHz, CDCl_3_): δ = 0.11 (s, 3 H, CH_3_^TBDMS^), 0.12 (s, 3 H, CH_3_^TBDMS^), 0.90 (s, 9 H, *t*-Bu^TBDMS^), 1.95 (d, *J* = 1.1 Hz, 3 H, CH_3_^T^), 2.13 (app. dt, *J* = 7.0 Hz, *J* = 13.7 Hz, 1 H, H2′a), 2.31 (ddd, *J_2′b,3′_* = 4.1 Hz, *J_1′,2′b_* = 6.4 Hz, *J_2′a,2′b_* = 13.7 Hz, 1 H, H2′b), 3.38 (dd, *J_4′,5′a_* = 4.1 Hz, *J_5′a,5′b_* = 10.8 Hz, 1 H, H5′a), 3.46 (dd, *J_4′,5′b_* = 4.6 Hz, *J_5′a,5′b_* = 10.8 Hz, 1 H, H5′b), 3.70 (app. q, *J* = 4.1 Hz, 1 H, H4′), 4.25 (app. dt, *J* = 4.1 Hz, *J* = 7.0 Hz, 1 H, H3′), 4.70 (s, 2 H, O-CH_2_-Ph), 5.49 (s, 2 H, N-CH_2_-O), 6.29 (dd, *J_1′,2′b_* = 6.4 Hz, *J_1′,2′a_* = 7.0 Hz, 1 H, H1′), 7.23–7.27 (m, 1 H, H^ar^), 7.30–7.33 (m, 2 H, H^ar^), 7.36–7.38 (m, 2 H, H^ar^), 7.44 (app. d, *J* = 1.1 Hz, 1 H, H6) ppm. ^13^C NMR (100 MHz, CDCl_3_): δ = −4.5 (CH_3_^TBDMS^), −4.4 (CH_3_^TBDMS^), 7.2 (C5′), 13.5 (CH_3_^T^), 18.0 (qC, *t*-Bu^TBDMS^), 25.8 (*t*-Bu^TBDMS^), 40.7 (C2′), 70.7 (N-CH_2_-O), 72.4 (O-CH_2_-Ph), 75.2 (C3′), 84.3 (C4′), 85.4 (C1′), 110.6 (C5), 127.77 (Ph), 127.81 (Ph), 128.4 (Ph), 134.7 (C6), 138.1 (qC, Ph), 150.9 (C2), 163.5 (C4) ppm. HRMS (APCI^+^): *m/z* calc. 587.1433 [M + H]^+^, found: 587.1421.

***C*-(*N^3^*-Benzyloxymethyl-3′-*O*-(*tert*-butyldimethylsilyl)-5′-deoxythymidin-5′-yl) benzyl methyl malonate (6):** NaH (60% dispersion in mineral oil, 0.153 g, 3.82 mmol) was suspended in dry DMF (10 mL) under argon and benzyl methyl malonate (2.1 mL, 11.7 mmol) was added. BOM-protected iodide **5** (1.117 g, 1.91 mmol) was dissolved in dry DMF (10 mL) and added dropwise, the reaction mixture was heated to 100 °C and stirred for 24 h. After this time, TLC analysis (CH_2_Cl_2_-EtOAc, 19:1) showed the consumption of starting material (R_f_ = 0.4) and the formation of the product (R_f_ = 0.3). The reaction was cooled to r.t., diluted with EtOAc (40 mL) and washed with H_2_O (20 mL). The aqueous layer was extracted with EtOAc (2 × 40 mL) and the combined organic extracts were dried over MgSO_4_, filtered and concentrated. Purification by flash column chromatography (CH_2_Cl_2_-EtOAc, 19:1) afforded the desired product **6** as a yellow oil (0.838 g, 66%). Compound **6** was isolated as a 1:1 mixture of interconverting diastereoisomers. ν_max_/cm^−1^ (neat) 2953 (CH), 2930 (CH), 2857 (CH), 1734 (C=O), 1710 (C=O), 1656 (C=O), 1464 (CH), 1361, 1253, 1151, 1087 (C-O), 1046 (C-O), 833, 773, 735, 696. ^1^H NMR (600 MHz, CDCl_3_): δ = 0.07–0.08 (m, 12 H, 4 × CH_3_^TBDMS^), 0.88–0.89 (2 × s, 18 H, *t*-Bu^TBDMS^), 1.91 (d, *J* = 1.1 Hz, 3 H, CH_3_^T^), 1.95 (d, *J* = 1.1 Hz, 3 H, CH_3_^T^), 1.97–2.02 (m, 2 H, H2′a), 2.11–2.17 (m, 2 H, H5′a), 2.22–2.26 (m, 2 H, H2′b), 2.36–2.40 (m, 2 H, H5′b), 3.62–3.66 (m, 2 H, H6′), 3.68 (s, 3 H, CO_2_CH_3_), 3.73 (s, 3 H, CO_2_CH_3_), 3.80–3.84 (m, 2 H, H4′), 4.05–4.08 (m, 2 H, H3′), 4.70 (s, 4 H, 2 × O-CH_2_-Ph^BOM^), 5.12–5.18 (m, 4 H, 2 × CO_2_CH_2_^Bn^), 5.47 (s, 2 H, N-CH_2_-O), 5.48 (s, 2 H, N-CH_2_-O), 6.14 (app. t, *J* = 6.5 Hz, 1 H, H1′), 6.18 (app. t, *J* = 6.6 Hz, 1 H, H1′), 7.04 (app. d, *J* = 1.1 Hz, 1 H, H6), 7.10 (app. d, *J* = 1.1 Hz, 1 H, H6), 7.24–7.38 (m, 20 H, 4 × Ph) ppm. ^13^C NMR (151 MHz, CDCl_3_): δ = −4.8 (CH_3_^TBDMS^), −4.63 (CH_3_^TBDMS^), −4.61 (CH_3_^TBDMS^), 13.16 (CH_3_^T^), 13.20 (CH_3_^T^), 17.9 (qC, *t*-Bu^TBDMS^), 25.7 (*t*-Bu^TBDMS^), 32.59 (C5′), 32.61 (C5′), 40.48 (C2′), 40.55 (C2′), 48.9 (C6′), 49.1 (C6′), 52.72 (CO_2_CH_3_), 52.78 (CO_2_CH_3_), 67.3 (CO_2_CH_2_^Bn^), 67.4 (CO_2_CH_2_^Bn^), 70.5 (N-CH_2_-O), 70.6 (N-CH_2_-O), 72.2 (O-CH_2_-Ph^BOM^), 74.95 (C3′), 75.03 (C3′), 84.3 (C4′), 84.5 (C4′), 85.62 (C1′), 85.64 (C1′), 110.3 (C5), 110.4 (C5), 127.6 (Ph), 127.7 (Ph), 127.9 (Ph), 128.1 (Ph), 128.3 (Ph), 128.4 (Ph), 128.47 (Ph), 128.48 (Ph), 128.6 (Ph), 134.03 (C6), 134.05 (C6), 135.23 (qC, CO_2_Bn), 135.24 (qC, CO_2_Bn), 138.0 (qC, OBn^BOM^), 150.77 (C2), 150.80 (C2), 163.37 (C4), 163.39 (C4), 168.5 (CO_2_Bn), 169.1 (CO_2_CH_3_), 169.3 (CO_2_Bn), 169.8 (CO_2_CH_3_) ppm. HRMS (APCI^+^): *m/z* calc. 667.3045 [M + H]^+^, found: 667.3054

***C*-(*N^3^*-Benzyloxymethyl-5′-deoxythymidin-5′-yl) benzyl methyl malonate (7):** Protected malonate **6** (100 mg, 150 µmol) was dissolved in THF (1.5 mL). Tetra-*n*-butylammonium fluoride trihydrate (85 mg, 270 µmol) was added and the solution was stirred at r.t. for 1 h. After this time, TLC analysis (CH_2_Cl_2_-EtOAc, 9:1) showed the complete consumption of the starting material (R_f_ = 0.7) and product formation (R_f_ = 0.2). The yellow solution was concentrated and purification by flash column chromatography (CH_2_Cl_2_-MeOH, 99:1→19:1) afforded the desired product **7** as a colourless wax (59 mg, 71%). Compound **7** was isolated as a 1:1 mixture of interconverting diastereoisomers. ν_max_/cm^−1^ (CH_2_Cl_2_) 3458 (OH), 3033 (CH), 2955 (CH), 2928 (CH), 1732 (C=O), 1705 (C=O), 1641 (C=O), 1467 (CH), 1454 (CH), 1438, 1361, 1273, 1222, 1153, 1088 (C-O), 1074 (C-O), 1048, 1028, 774, 737, 697. ^1^H NMR (400 MHz, acetone-*d*_6_): δ = 1.83 (d, *J_CH3T,6_* = 1.1 Hz, 3 H, CH_3_^T^), 1.87 (d, *J_CH3T,6_* = 1.1 Hz, 3 H, CH_3_^T^), 2.17–2.33 (m, 6 H, H2′a, H2′b, H5′a), 2.38 (ddd, *J* = 3.8 Hz, *J* = 7.8 Hz, *J* = 14.2 Hz, 2 H, H5′b), 3.65 (s, 3 H, CO_2_CH_3_), 3.69 (s, 3 H, CO_2_CH_3_), 3.69–3.74 (m, 2 H, H6′), 3.83–3.89 (m, 2 H, H4′), 4.25–4.30 (m, 2 H, H3′), 4.53 (d, *J* = 4.4 Hz, 1 H, OH), 4.54 (d, *J* = 4.4 Hz, 1 H, OH), 4.65 (s, 2 H, O-CH_2_-Ph^BOM^), 4.66 (s, 2 H, O-CH_2_-Ph^BOM^), 5.16 (d, *J* = 3.5 Hz, 2 H, CO_2_CH_2_^Bn^), 5.20 (d, *J* = 3.7 Hz, 2 H, CO_2_CH_2_^Bn^), 5.44 (m, 2 H, N-CH_2_-O), 5.45 (s, 2 H, N-CH_2_-O), 6.26 (app. t, *J* = 6.8 Hz, 1 H, H1′), 6.28 (app. t, *J* = 6.8 Hz, 1 H, H1′), 7.22–7.38 (m, 21 H, 4 × Ph, H6), 7.41 (app. d, *J* = 1.1 Hz, 1 H, H6) ppm. ^13^C NMR (100 MHz, acetone-*d*_6_): δ = 13.1 (CH_3_^T^), 33.4 (C5′), 39.9 (C2′), 40.0 (C2′), 49.6 (C6′), 49.7 (C6′), 52.8 (CO_2_CH_3_), 67.5 (CO_2_CH_2_^Bn^), 71.26 (N-CH_2_-O), 71.28 (N-CH_2_-O), 72.4 (O-CH_2_-Ph^BOM^), 74.79 (C3′), 74.85 (C3′), 84.9 (C4′), 85.1 (C4′), 86.1 (C1′), 110.35 (C5), 110.43 (C5), 128.2 (Ph), 128.68 (Ph), 128.73 (Ph), 128.9 (Ph), 129.0 (Ph), 129.2 (Ph), 129.3 (Ph), 135.84 (C6), 135.88 (C6), 136.90 (qC, CO_2_Bn), 136.92 (qC, CO_2_Bn), 139.6 (qC, OBn^BOM^), 151.79 (C2), 151.81 (C2), 163.79 (C4), 163.81 (C4), 169.4 (CO_2_Bn), 169.91 (CO_2_CH_3_), 169.94 (CO_2_Bn), 170.4 (CO_2_CH_3_) ppm. HRMS (APCI^+^): *m/z* calc. 575.2000 [M + Na]^+^, found: 575.2015.

***C*-(5′-Deoxythymidin-5′-yl) malonic acid monomethyl ester (8):** Protected malonate **7** (115 mg, 208 µmol) was dissolved in MeOH (10 mL) and added to a dried flask containing Pd/C (10%, 17 mg) and H_2_ was bubbled through the suspension while stirring at r.t. for 28 h. After this time, TLC analysis (CH_2_Cl_2_-MeOH, 9:1) showed the complete consumption of starting material (R_f_ = 0.6) and the formation of the product (R_f_ = 0.2). The reaction mixture was filtered through celite and the filtrate was concentrated to a colourless film. Purification by flash column chromatography (CH_2_Cl_2_-MeOH, 19:1→MeOH) afforded the desired product **8** as a white foam (61 mg, 86%). Compound **8** was isolated as a 1:1 mixture of interconverting diastereoisomers. ν_max_/cm^−1^ (neat) 3375 (OH, NH), 2955 (CH), 1686 (C=O), 1665 (C=O), 1588 (C=O), 1474 (CH), 1437 (OH), 1371, 1269, 1198, 1168, 1086 (CO), 1049, 767. ^1^H NMR (600 MHz, DMSO-*d*_6_): δ = 1.80 (2 × s, 6 H, CH_3_^T^), 1.89–1.95 (m, 1 H, H5′a), 1.98–2.06 (m, 4 H, H2′a, H5′a, H5′b), 2.08–2.16 (m, 3 H, H2′b, H5′b), 3.06 (app. t, *J* = 6.9 Hz, 1 H, H6′), 3.13–3.15 (m, 1 H, H6′), 3.49 (s, 3 H, CO_2_CH_3_), 3.53 (s, 3 H, CO_2_CH_3_), 3.59–3.62 (m, 1 H, H4′), 3.68–3.71 (m, 1 H, H4′), 4.00–4.02 (m, 1 H, H3′), 4.03–4.06 (m, 1 H, H3′), 4.14 (bs, 2 H, OH), 5.53 (bs, 2 H, OH), 6.05 (app. t, *J* = 6.9 Hz, 1 H, H1′), 6.09 (app. t, *J* = 6.9 Hz, 1 H, H1′), 7.36 (s, 1 H, H6), 7.42 (s, 1 H, H6), 11.27 (bs, 2 H, NH) ppm. ^13^C NMR (150 MHz, DMSO-*d*_6_): δ = 12.12 (CH_3_^T^), 12.13 (CH_3_^T^), 33.4 (C5′), 33.8 (C5′), 38.7 (C2′), 50.9 (CO_2_CH_3_), 51.4 (C6′), 52.1 (C6′), 73.1 (C3′), 73.4 (C3′), 83.38 (C1′), 83.42 (C1′), 84.2 (C4′), 85.4 (C4′), 109.5 (C5), 109.9 (C5), 136.0 (C6), 150.4 (C2), 150.5 (C2), 163.8 (C4), 172.3 (CO_2_), 172.8 (CO_2_) ppm. HRMS (APCI^−^): *m/z* calc. 341.0990 [M − H]^−^, found: 341.0990.

***C*-(5′-Deoxythymidin-5′-yl) monomethyl sodium malonate (9):** Protected malonate **7** (250 mg, 452 µmol) was dissolved in MeOH (15 mL) and added to a dried flask containing Pd/C (10%, 50 mg) and H_2_ was bubbled through the suspension while stirring at r.t. for 7 h. After this time, TLC analysis (EtOAc-MeOH, 2:1) showed the complete consumption of starting material (R_f_ = 0.9) and the formation of the product (R_f_ = 0.2). The reaction mixture was filtered through celite and the filtrate was concentrated to a colourless film. Purification by flash column chromatography (EtOAc-MeOH, 3:1→2:1) was followed by elution through Diaion resin WT01S(H) (Na form) in MeOH. The resulting white solid was purified by preparative reversed-phase TLC (H_2_O-MeCN, 1:1), extracted with MeOH and concentrated to afford the desired product **9** as a white solid (37 mg, 22%). Compound **9** was isolated as a 1:1 mixture of interconverting diastereomers. ν_max_/cm^−1^ (neat) 3435 (OH, NH), 3210 (CH), 2951 (CH), 1665 (C=O), 1594 (C=O), 1473 (CH), 1436, 1369, 1269, 1195, 1165, 1084 (C-O), 1048, 768. ^1^H NMR (600 MHz, DMSO-*d*_6_): δ = 1.80 (d, *J_CH3T,6_* = 0.9 Hz, 6 H, CH_3_^T^), 1.93–2.17 (m, 8 H, H2′a, H2′b, H5′a, H’5b), 3.18 (app. t, *J* = 6.9 Hz, 1 H, H6′), 3.23 (app. t, *J* = 9.4 Hz, 1 H, H6′), 3.52 (s, 3 H, CO_2_CH_3_), 3.56 (s, 3 H, CO_2_CH_3_), 3.59–3.62 (m, 1 H, H4′), 3.65–3.68 (m, 1 H, H4′), 4.02–4.07 (m, 2 H, H3′), 6.07 (app. t, *J* = 6.8 Hz, 1 H, H1′), 6.10 (app. t, *J* = 6.9 Hz, 1 H, H1′), 7.37 (app. d, *J* = 0.9 Hz, 1 H, H6), 7.42 (app. d, *J* = 0.9 Hz, 1 H, H6), 11.27 (bs, 2 H, NH) ppm. ^13^C NMR (150 MHz, DMSO-*d*_6_): δ = 12.1 (CH_3_^T^), 33.0 (C5′), 33.3 (C5′), 38.5 (C2′), 38.6 (C2′), 50.4 (C6′), 50.8 (C6′), 51.3 (CO_2_CH_3_), 73.1 (C3′), 73.3 (C3′), 83.4 (C1′), 84.0 (C4′), 84.9 (C4′), 109.6 (C5), 109.9 (C5), 136.05 (C6), 136.10 (C6), 150.42 (C2), 150.45 (C2), 163.7 (C4), 170.1 (CO_2_Na), 170.3 (CO_2_Na), 171.5 (CO_2_CH_3_), 171.8 (CO_2_CH_3_) ppm. HRMS (ESI^+^): *m/z* calc. 373.0618 [M + H]^+^, found: 373.0618.

***C*-(5′-Deoxythymidin-5′-yl) disodium malonate (10):** KOH (20 mg, 356 µmol) was dissolved in MeOH (0.5 mL) and cooled to 0 °C. Ester **8** (30 mg, 88 µmol) was added and the reaction was slowly warmed to r.t. and stirred for 3 h. A further portion of KOH (20 mg, 356 µmol) in MeOH (0.5 mL) was added and the reaction was stirred for a further 4 h. After this time, TLC analysis (H_2_O-*i*-PrOH-EtOAc, 1:2:2) showed the consumption of starting material (R_f_ = 0.7) and the formation of a product (R_f_ = 0.6). The reaction mixture was concentrated and the residue was taken up in H_2_O and eluted through Diaion resin WT01S(H) (H form) to remove excess KOH, followed by elution through Diaion resin WT01S(H) (Na form). The white solid was purified by preparative reversed-phase TLC (H_2_O-MeCN, 1:1), extracted with H_2_O and concentrated to afford the desired product **10** as a white solid (18 mg, 55%); mp 166–170 °C dec. ν_max_/cm^−1^ (neat) 3316 (OH, NH), 2937 (CH), 2822 (CH), 1686 (C=O), 1587 (C=O), 1475 (CH), 1420, 1365, 1271, 1082 (C-O), 1024, 767. ^1^H NMR (600 MHz, D_2_O): δ = 1.92 (s, 3 H, CH_3_^T^), 2.17–2.23 (m, 1 H, H5′a), 2.29–2.41 (m, 3 H, H2′a, H2′b, H5′b), 3.32–3.40 (m, 1 H, H6′), 3.97 (bs, 1 H, H4′), 4.34–4.36 (m, 1 H, H3′), 6.24–6.26 (m, 1 H, H1′), 7.51 (s, 1 H, H6) ppm. ^13^C NMR (150 MHz, D_2_O): δ = 11.5 (CH_3_^T^), 33.2 (C5′), 37.8 (C2′), 55.2 (C6′), 73.6 (C3′), 84.8 (C1′), 85.0 (C4′), 111.5 (C5), 137.4 (C6), 151.7 (C2), 166.5 (C4), 176.5 (CO_2_), 176.6 (CO_2_) ppm. HRMS (ESI^+^): *m/z* calc. 373.0618 [M + H]^+^, found: 373.0618.

***C-(*5′-deoxythymidin-5′-yl) sodium *N*-hydroxymalonamide(11):** Hydroxylamine hydrochloride (426 mg, 6.13 mmol) was suspended in MeOH (6 mL) and KOH (430 mg, 7.66 mmol) was added. The suspension was warmed to 40 °C to aid dissolution and the precipitate was removed by filtration. Ester **8** (93 mg, 272 µmol) was dissolved in the filtrate and the reaction was stirred at r.t. for 5 h. After this time, TLC analysis showed the consumption of starting material (CH_2_Cl_2_-MeOH, 4:1, R_f_ = 0.8) and the formation of a product (H_2_O-*i*-PrOH-EtOAc, 1:2:2, R_f_ = 0.3). The pH was adjusted to pH 7 using 1 M aq. HCl to quench the reaction. The product was purified by flash column chromatography (CH_2_Cl_2_-MeOH, 4:1→MeOH followed by H_2_O-*i*-PrOH-EtOAc, 1:5:4→1:2:2), followed by elution through Diaion resin WT01S(H) (Na form). The resulting white solid was purified by preparative reversed-phase TLC (H_2_O-MeCN, 1:1), extracted with MeOH and concentrated to afford the desired product **11** as a white solid (15 mg, 15%). Compound **11** was isolated as a 1:1 mixture of interconverting diastereomers. ν_max_/cm^−1^ (neat) 3424 (OH, NH), 3213 (CH), 3045 (CH), 2936 (CH), 2818 (CH), 1655 (C=O), 1604 (C=O), 1579 (C=O), 1515, 1474 (CH), 1447, 1369 (C=O), 1272 (CO), 1126 (C-O), 1083 (C-O), 958, 770. ^1^H NMR (600 MHz, D_2_O): δ = 1.92 (s, 3 H, CH_3_^T^), 1.93 (s, 3 H, CH_3_^T^), 2.08–2.15 (m, 2 H, H5′a), 2.27–2.33 (m, 2 H, H’5b), 2.34–2.42 (m, 4 H, H2′a, H2′b), 3.19 (app. t, *J* = 7.2 Hz, 1 H, H6′), 3.23 (dd, *J* = 4.6 Hz, *J* = 10.4 Hz, 1 H, H6′), 3.82 (app. dt, *J* = 4.0 Hz, *J* = 9.6 Hz, 1 H, H4′), 3.92–3.94 (m, 1 H, H4′), 4.31–4.34 (m, 2 H, H3′), 6.22–6.27 (m, 2 H, H1′), 7.48 (s, 1 H, H6), 7.50 (s, 1 H, H6) ppm. ^13^C NMR (150 MHz, D_2_O): δ = 11.48 (CH_3_^T^), 11.52 (CH_3_^T^), 32.7 (C5′), 32.9 (C5′), 37.7 (C2′), 37.8 (C2′), 49.3 (C6′), 50.0 (C6′), 73.5 (C3′), 73.7 (C3′), 83.9 (C4′), 84.8 (C1′), 84.9 (C1′), 85.1 (C4′), 111.5 (C5), 111.7 (C5), 137.3 (C6), 137.4 (C6), 151.69 (C2), 151.71 (C2), 166.5 (C4), 166.6 (C4), 170.0 (CONHO), 170.4 (CONHO), 175.5 (CO_2_Na), 175.8 (CO_2_Na) ppm. HRMS (ESI^+^): *m/z* calc. 388.0727 [M + Na]^+^, found: 388.0731.

***N-(*5′-Deoxythymidin-5′-yl) amido methyl malonate (13):** Aminothymidine **12** (500 mg, 2.07 mmol) was dissolved in dry DMF (15 mL), under argon. EDC·HCl (437 mg, 2.28 mmol) was suspended in dry DMF (20 mL) under argon and cooled to 0 °C. Monomethyl potassium malonate (356 mg, 2.28 mmol) was added and the suspension was stirred at 0 °C for 1 h. After this time, the aminothymidine solution was added followed by 4-(dimethylamino)pyridine (25 mg, 0.21 mmol). The reaction was slowly warmed to r.t. and stirred for 20 h. After this time, TLC analysis (CH_2_Cl_2_-MeOH, 4:1) showed the consumption of amine starting material, (R_f_ = 0) and the formation of the product (R_f_ = 0.7). The reaction mixture was concentrated and purification by flash column chromatography afforded the desired product **13** as a white solid (385 mg, 54%); mp 68–72 °C dec. ν_max_/cm^−1^ (neat) 3432 (OH, NH), 3290 (CH), 3098 (CH), 2955 (CH), 1755, 1708, 1655 (C=O), 1637 (C=O), 1567, 1473 (CH), 1402, 1365, 1259, 1218, 1198, 1153, 1100 (C-O), 1060, 1042, 1014, 955, 902, 852, 782. ^1^H NMR (600 MHz, DMSO-*d*_6_): δ = 1.79 (d, *J_CH3T,6_* = 1.1 Hz, 3 H, CH_3_^T^), 2.04 (ddd, *J* = 3.3 Hz, *J_1′,2′a_* = 6.3 Hz, *J_2′a,2′b_* = 13.5 Hz, 1 H, H2′a), 2.13 (ddd, *J* = 6.5 Hz, *J_1′,2′b_* = 7.9 Hz, *J_2′a,2′b_* = 13.5 Hz, 1 H, H2′b), 3.24–3.30 (m, 3 H, α-CH_2_, H5′a), 3.40 (ddd, *J_4′,5′b_* = 4.6 Hz, *J_NH,5′b_* = 5.8 Hz, *J_5′a,5′b_* = 14.0 Hz, 1 H, H5′b), 3.60 (s, 3 H, OCH_3_), 3.73 (ddd, *J* = 3.6 Hz, *J_4′,5′b_* = 4.6 Hz, *J* = 6.9 Hz, 1 H, H4′), 4.13–4.16 (m, 1 H, H3′), 5.30 (d, *J* = 4.0 Hz, 1 H, OH), 6.14 (dd, *J_1′,2′a_* = 6.3 Hz, *J_1′,2′b_* = 7.9 Hz, 1 H, H1′), 7.46 (app. d, *J* = 1.1 Hz, 1 H, H6), 8.28 (app. t, *J* = 5.8 Hz, CONH), 11.29 (bs, 1 H, NH^T^) ppm. ^13^C NMR (150 MHz, DMSO-*d*_6_): δ = 12.0 (CH_3_^T^), 38.4 (C2′), 41.0 (C5′), 42.2 (α-CH_2_), 51.8 (OCH_3_), 71.1 (C3′), 83.7 (C1′), 84.8 (C4′), 109.8 (C5), 136.2 (C6), 150.5 (C2), 163.7 (C4), 165.5 (CONH), 168.4 (COOCH_3_) ppm. HRMS (APCI^−^): *m/z* calc. 342.1296 [M + H]^+^, found: 342.1299.

***N-(*5′-Deoxythymidin-5′-yl) amido sodium malonate (14):** KOH (164 mg, 2.92 mmol) was dissolved in MeOH (6 mL). Ester **11** (100 mg, 293 µmol) was added and the reaction was stirred for 2 h. After this time, TLC analysis (CH_2_Cl_2_-MeOH, 4:1) showed the complete consumption of starting material (R_f_ = 0.8) and the formation of a product (R_f_ = 0.0). The reaction mixture was eluted through Diaion resin WT01S(H) (H form) to remove excess KOH, followed by elution through Diaion resin WT01S(H) (Na form) and concentrated to afford the desired product **14** as a white foam (100 mg, 98%). ν_max_/cm^−1^ (neat) 3281 (OH, NH), 2932 (CH), 1648 (C=O), 1595 (C=O), 1471 (CH), 1373 (C=O), 1316, 1270, 1087 (CH), 1049, 731. ^1^H NMR (400 MHz, D_2_O): δ = 1.89 (d, *J_CH3T,6_* = 1.1 Hz, 3 H, CH_3_^T^), 2.31–2.43 (m, 2 H, H2′a, H2′b), 3.21 (s, 2 H, α-CH_2_), 3.50–3.59 (m, 2 H, H5′a, H5′b), 4.02–4.06 (m, 1 H, H4′), 4.43 (app. dt, *J* = 4.3 Hz, *J* = 6.3 Hz, 1 H, H3′), 6.28 (app. t, *J* = 6.9 Hz, 1 H, H1′), 7.45 (app. d, *J* = 1.1 Hz, 1 H, H6) ppm. ^13^C NMR (100 MHz, D_2_O): δ = 12.0 (CH_3_^T^), 37.8 (C2′), 40.6 (C5′), 45.0 (α-CH_2_), 71.2 (C3′), 85.0 (C1′), 84.1 (C4′), 111.7 (C5), 137.0 (C6), 154.6 (C2), 170.3 (C4), 171.6 (CONH), 174.9 (COONa) ppm. HRMS (ESI^+^): *m/z* calc. 372.0778 [M + Na]^+^, found: 372.0791.

***N-(*5′-Deoxythymidin-5′-yl) amido *N*-hydroxymalonamide (15):** Hydroxylamine hydrochloride (407 mg, 5.86 mmol) was suspended in MeOH (6 mL) and KOH (411 mg, 7.33 mmol) was added. The suspension was warmed to 40 °C to aid dissolution and the precipitate was removed by filtration. Ester **13** (100 mg, 293 µmol) was dissolved in the filtrate and the reaction was stirred at r.t. for 4 h. After this time, TLC analysis showed the complete consumption of starting material (CH_2_Cl_2_-MeOH, 4:1, R_f_ = 0.8) and the formation of a product (H_2_O-*i*-PrOH-EtOAc, 1:5:4, R_f_ = 0.6). The pH was adjusted to pH 7 using 1 M aq. HCl to quench the reaction. Purification by flash column chromatography (H_2_O-*i*-PrOH-EtOAc, 1:10:9) afforded the desired product **15** as a white foam (83 mg, 83%). ν_max_/cm^−1^ (neat) 3205 (OH, NH), 3068 (CH), 2934 (CH), 2826 (CH), 1638 (C=O), 1551, 1475 (CH), 1414, 1368, 1270, 1088 (C-O), 1053. ^1^H NMR (600 MHz, DMSO-*d*_6_): δ = 1.80 (d, *J_CH3T,6_* = 1.0 Hz, 3 H, CH_3_^T^), 2.02 (ddd, *J* = 3.1 Hz, *J_1′,2′a_* = 6.2 Hz, *J_2′a,2′b_* = 13.5 Hz, 1 H, H2′a), 2.15 (ddd, *J* = 6.6 Hz, *J_1′,2′b_* = 7.9 Hz, *J_2′a,2′b_* = 13.5 Hz, 1 H, H2′b), 2.93 (d, *J_αa,αb_* = 14.2 Hz, 1 H, α-CH_a_), 2.97 (d, *J_αa,αb_* = 14.2 Hz, 1 H, α-CH_b_), 3.29–3.34 (m, 2 H, H5′a, H5′b), 3.72–3.74 (m, 1 H, H4′), 4.14–4.17 (m, 1 H, H3′), 5.33 (d, *J* = 4.3 Hz, 1 H, 3′-OH), 6.14 (dd, *J_1′,2′a_* = 6.2 Hz, *J_1′,2′b_* = 7.9 Hz, 1 H, H1′), 7.54 (app. d, *J* = 1.0 Hz, 1 H, H6), 8.24 (app. t, *J* = 5.9 Hz, CONHC), 8.91 (s, 1 H, NOH), 10.60 (bs, 1 H, NHO), 11.28 (bs, 1 H, NH^T^) ppm. ^13^C NMR (150 MHz, DMSO-*d*_6_): δ = 12.1 (CH_3_^T^), 38.4 (C2′), 40.7 (α-CH_2_), 40.9 (C5′), 71.0 (C3′), 83.5 (C1′), 84.8 (C4′), 109.8 (C5), 136.3 (C6), 150.5 (C2), 163.8 (C4), 163.9 (COONa), 166.7 (CONH) ppm. HRMS (APCI^+^): *m/z* calc. 343.1248 [M + H]^+^, found: 343.1251.

**1-[5′-*O*-(4,4′-Dimethoxytrityl)-2′-deoxy-β-D-furanosyl]thymine (17):** Alcohol **17** was prepared according to a modified published procedure [32]. Protected thymidine **16** (5.00 g, 9.18 mmol) was dissolved in dry THF (40 mL) under argon, Et_3_N (3.2 mL, 22.95 mmol) was added and the solution was cooled to 0 °C. Methylsulfonate chloride (1.1 mL, 13.77 mmol) was added dropwise and the resulting suspension was gradually warmed to r.t. and stirred for 1 h. After this time, TLC analysis (EtOAc) showed the complete consumption of starting material (R_f_ = 0.5) and the formation of the intermediate (R_f_ = 0.6). EtOH (20 mL) and aq. NaOH (50 mL, 10 M) were added to the reaction mixture and it was heated to 60 °C for 17 h. After this time, TLC analysis (CH_2_Cl_2_-MeOH, 19:1) showed the complete consumption of the intermediate (R_f_ = 0.6) and the formation of the product (R_f_ = 0.1). The reaction mixture was cooled to r.t. and the organic solvents were removed under reduced pressure. The mixture was neutralised with aq. HCl and extracted with CH_2_Cl_2_ (3 × 150 mL). The combined organic extracts were washed with H_2_O (200 mL) and brine (200 mL), dried over MgSO_4_, filtered and concentrated to afford the desired product **17** as a yellow foam (4.48 g, 90%). ν_max_/cm^−1^ (neat) 3396 (OH, NH), 3171 (OH, NH), 3004 (CH), 2954 (CH), 2932 (CH), 2836, 1682 (C=O), 1607 (C=O), 1508, 1464 (CH), 1445, 1271, 1247, 1175, 1065 (C-O), 1032, 903, 828, 755, 701. ^1^H NMR (400 MHz, DMSO-*d*_6_): δ = 1.64 (s, 3 H, CH_3_^T^), 1.86 (dd, *J_1′,2′a_* = 2.0 Hz, *J_2′a,2′b_* = 14.6 Hz, 1 H, H2′a), 2.52–2.57 (m, 1 H, H2′b), 3.19 (dd, *J_4′,5′a_* = 2.8 Hz, *J_5′a,5′b_* = 10.3 Hz, 1 H, H5′a), 3.38 (dd, *J_4′,5′b_* = 8.1 Hz, *J_5′a,5′b_* = 10.3 Hz, 1 H, H5′b), 3.73 (s, 6 H, 2 × OCH_3_), 4.08 (app. dt, *J* = 2.8 Hz, *J* = 8.1 Hz, 1 H, H4′), 4.18–4.21 (m, 1 H, H3′), 5.19 (d, *J_3′,OH_* = 3.3 Hz, 1 H, OH), 6.11 (dd, *J_1′,2′a_* = 2.0 Hz, *J_1′,2′b_* = 8.1 Hz, 1 H, H1′), 6.86–6.89 (m, 4 H, H^ar,DMTr^), 7.20–7.24 (m, 1 H, H^ar,DMTr^), 7.27–7.31 (m, 6 H, H^ar,DMTr^), 7.42–7.43 (m, 2 H, H^ar,DMTr^), 7.60 (s, 1 H, H6), 11.26 (s, 1 H, NH) ppm. ^13^C NMR (100 MHz, DMSO-*d*_6_): δ = 12.4 (CH_3_^T^), 40.8 (C2′), 55.0 (OCH_3_), 62.8 (C5′), 69.0 (C3′), 83.3 (C4′), 84.2 (C1′), 85.5 (qC, DMTr), 108.3 (C5), 113.1 (DMTr), 126.6 (DMTr), 127.75 (DMTr), 127.78 (DMTr), 129.7 (DMTr), 129.8 (DMTr), 135.5 (qC, DMTr), 135.7 (qC, DMTr), 136.8 (C6), 144.9 (qC, DMTr), 150.5 (C2), 158.0 (qC, DMTr), 163.8 (C4) ppm. HRMS (APCI^−^): *m/z* calc. 543.2137 [M − H]^−^, found: 543.2141. Spectroscopic data are in agreement with the literature [32].

**1-[5′-*O*-(4,4′-dimethoxytrityl)-3′-*O*-mesyl-2′-deoxy-β-D-furanosyl]thymine (18):** Mesylated thymidine **18** was prepared according to a published procedure [33]. Inverted thymidine **17** (300 mg, 0.55 mmol) was dissolved in dry pyridine (5 mL) under argon and the solution was cooled to 0 °C. Methylsulfonate chloride (130 µL, 1.68 mmol) was added dropwise and the reaction mixture was gradually warmed to r.t. and stirred for 18 h. After this time, TLC analysis (CH_2_Cl_2_-MeOH, 19:1) showed the complete consumption of starting material (R_f_ = 0.1) and the formation of the product (R_f_ = 0.6). The reaction mixture was cooled to 0 °C and H_2_O (0.4 mL) was added. After 5 min, the reaction mixture was poured into ice-H_2_O (60 mL) with vigorous stirring. The resulting precipitate was collected by vacuum filtration and washed with H_2_O (10 mL) to afford the desired product **18** as a white solid (309 mg, 90%); mp 88–92 °C dec (lit. [40] 85.4–86.7 °C) ν_max_/cm^–1^ (neat) 3181 (OH, NH), 3058 (CH), 2955 (CH), 2930 (CH), 2856 (CH), 1689 (C=O), 1608, 1508, 1464 (CH), 1446, 1250, 1175, 1097 (C-O), 1033, 831, 779, 702. ^1^H NMR (400 MHz, acetone-*d*_6_): δ = 1.73 (d, *J_CH3T,6_* = 1.1 Hz, 3 H, CH_3_^T^), 2.45 (ddd, *J_2′a,3′_* = 1.0 Hz, *J_1′,2′a_* = 3.2 Hz, *J_2′a,2′b_* = 15.7 Hz, 1 H, H2′a), 2.97 (ddd, *J_2′b,3′_* = 5.5 Hz, *J_1′,2′b_* = 8.0 Hz, *J_2′a,2′b_* = 15.7 Hz, 1 H, H2′b), 3.02 (s, 3 H, SO_2_CH_3_), 3.39 (dd, *J_4′,5′a_* = 4.9 Hz, *J_5′a,5′b_* = 10.1 Hz, 1 H, H5′a), 3.61 (dd, *J_4′,5′b_* = 6.6 Hz, *J_5′a,5′b_* = 10.1 Hz, 1 H, H5′b), 3.79 (s, 6 H, 2 × OCH_3_), 4.43–4.46 (m, 1 H, H4′), 5.41–5.44 (m, 1 H, H3′), 6.29 (dd, *J_1′,2′a_* = 3.2 Hz, *J_1′,2′b_* = 8.0 Hz, 1 H, H1′), 6.89–6.91 (m, 4 H, H^ar,DMTr^), 7.22–7.26 (m, 1 H, H^ar,DMTr^), 7.30–7.34 (m, 2 H, H^ar,DMTr^), 7.36–7.41 (m, 5 H, H6, 4 × H^ar,DMTr^), 7.51–7.53 (m, 2 H, H^ar,DMTr^), 9.96 (bs, 1 H, NH) ppm. ^13^C NMR (100 MHz, acetone-*d*_6_): δ = 12.6 (CH_3_^T^), 38.4 (SO_2_CH_3_), 40.0 (C2′), 55.5 (OCH_3_), 62.5 (C5′), 80.4 (C3′), 82.1 (C4′), 84.6 (C1′), 87.5 (qC, DMTr), 110.8 (C5), 113.96 (DMTr), 113.99 (DMTr), 127.7 (DMTr), 128.7 (DMTr), 129.0 (DMTr), 131.01 (DMTr), 131.03 (DMTr), 136.2 (C6), 136.5 (qC, DMTr), 136.6 (qC, DMTr), 145.9 (qC, DMTr), 151.3 (C2), 159.7 (qC, DMTr), 164.2 (C4) ppm. HRMS (APCI^−^): *m/z* calc. 621.1912 [M − H]^−^, found: 621.1928. Spectroscopic data are in agreement with the literature [33]. 

**3′-Deoxy-2′,3′-didehydro-5′-*O*-(4,4′-dimethoxytrityl)-*N^3^*-methylthymidine (20):** Compound **20** was obtained from the attempted synthesis of compound **19**. NaH (60% dispersion in mineral oil, 39 mg, 964 µmol) was suspended in dry DMF (1.0 mL) and benzyl methyl malonate (175 µL, 1.96 mmol) was added dropwise. Mesylate **18** (100 mg, 161 µmol) was dissolved in dry DMF (0.5 mL) under argon and added dropwise to the mixture of NaH and benzyl methyl malonate. The reaction mixture was heated to 100 °C for 120 h. After this time, TLC analysis (petroleum ether-EtOAc, 2:1) showed the consumption of starting material (R_f_ = 0.2) and the formation of a product (R_f_ = 0.6). The reaction mixture was cooled to r.t. and H_2_O (10 mL) was added. The mixture was extracted with EtOAc (3 × 20 mL) and the combined organic extracts were washed with brine (20 mL), dried over MgSO_4_, filtered and concentrated. Purification by flash column chromatography (EtOAc-petroleum ether, 1:3→EtOAc) did not afford the desired product **19** but afforded byproduct **20** as a white solid (40 mg, 46%). ν_max_/cm^−1^ (CH_2_Cl_2_) 3057 (CH), 2955 (CH), 2929 (CH), 2871 (CH), 2839 (CH), 1702 (C=O), 1667 (C=O), 1638 (C=O), 1608 (C=C), 1508, 1467 (CH), 1446, 1294, 1248, 1176, 1089 (C-O), 1033, 991, 829, 766, 733, 700. ^1^H NMR (600 MHz, acetone-*d*_6_): δ = 1.33 (d, *J_CH3T,6_* = 0.9 Hz, 3 H, CH_3_^T^), 3.27 (s, 3 H, N-CH_3_), 3.33 (dd, *J_4′,5′a_* = 2.7 Hz, *J_5′a,5′b_* = 10.4 Hz, 1 H, H5′a), 3.39 (dd, *J_4′,5′b_* = 4.8 Hz, *J_5′a,5′b_* = 10.4 Hz, 1 H, H5′b), 3.78 (2 × s, 6 H, 2 × OCH_3_), 5.02–5.04 (m, 1 H, H4′), 6.02–6.03 (m, 1 H, H2′), 6.55 (app. dt, *J* = 1.6 Hz, *J* = 6.0 Hz, 1 H, H3′), 6.86-6.88 (m, 4 H, H^ar,DMTr^), 7.06 (app. dt, *J* = 1.6 Hz, *J* = 3.5 Hz, 1 H, H1′), 7.23–7.25 (m, 1 H, H^ar,DMTr^), 7.29–7.31 (m, 2 H, H^ar,DMTr^), 7.33–7.34 (m, 4 H, H^ar,DMTr^), 7.43 (d, *J_CH3T,6_* = 0.9 Hz, 1 H, H6), 7.47–7.48 (m, 2 H, H^ar,DMTr^) ppm. ^13^C NMR (150 MHz, acetone-*d*_6_): δ = 12.5 (CH_3_^T^), 27.8 (N-CH_3_), 55.5 (OCH_3_), 66.1 (C5′), 86.5 (C4′), 87.0 (qC, DMTr), 91.2 (C1′), 110.0 (C5), 113.89 (DMTr), 113.90 (DMTr), 127.1 (C2′), 127.7 (DMTr), 128.6 (DMTr), 129.1 (DMTr), 131.02 (DMTr), 131.05 (DMTr), 135.0 (C6), 135.4 (C3′), 136.4 (qC, DMTr), 136.5 (qC, DMTr), 145.8 (qC, DMTr), 152.2 (C2), 159.70 (qC, DMTr), 159.73 (qC, DMTr), 163.9 (C4) ppm. HRMS (APCI^+^): *m/z* calc. 563.2153 [M + Na]^+^, found: 563.2151 

**5′-*O*-(4,4′-Dimethoxytrityl)-*N^3^*-methyl thymidine (23):** Compound **23** was obtained from the attempted synthesis of compound **22**. Anhydrosugar **21** (60 mg, 114 µmol) and 2,6-di-*tert*-butyl-4-methylpyridine (6 mg, 29 µmol) were dissolved in dry THF (2 mL) under argon and cooled to 10 °C. Methyl triflate (13 µL, 115 µmol) was added dropwise and the reaction was stirred for 10 min. NaH (60% dispersion in mineral oil, 6.7 mg, 168 µmol) was suspended in dry THF (0.5 mL) and benzyl methyl malonate (41 µL, 226 µmol) was added dropwise. The malonate solution was added dropwise to the reaction and it was stirred for 2.5 h. After this time, TLC analysis (EtOAc-MeOH, 19:1) showed the complete consumption of starting material (R_f_ = 0.2) and the formation of a product (R_f_ = 0.7). The reaction mixture was diluted with EtOAc (20 mL) and washed with brine (2 × 10 mL), dried over MgSO_4_, filtered and concentrated to an oil. Purification by flash column chromatography (EtOAc-petroleum ether, 1:1→2:1) did not afford the desired product **22** but afforded byproduct **23** as a white solid (20 mg, 31%). ν_max_/cm^−1^ (neat) 3429 (OH), 3058 (CH), 2952 (CH), 2933 (CH), 2837, 1694 (C=O), 1625 (C=O), 1609 (C=O), 1508, 1475, 1445, 1296 (CH), 1247, 1175, 1157, 1105 (C-O), 1072 (C-O), 1031, 828, 766, 754, 734, 701. ^1^H NMR (400 MHz, acetone-*d*_6_): δ = 1.75 (d, *J_CH3T,6_* = 1.1 Hz, 3 H, CH_3_^T^), 2.02–2.07 (m, 1 H, H2′a), 2.68 (dd, *J* = 5.2 Hz, *J_1′,2′b_* = 8.2 Hz, *J* = 13.4 Hz, 1 H, H2′b), 3.22 (s, 3 H, N-CH_3_), 3.39 (dd, *J* = 3.6 Hz, *J_5′a,5′b_* = 10.2 Hz, 1 H, H5′a), 3.61 (dd, *J* = 7.5 Hz, *J_5′a,5′b_* = 10.2 Hz, 1 H, H5′b), 3.78 (s, 6 H, 2 × OCH_3_), 4.19–4.22 (m, 1 H, H4′), 4.40–4.45 (m, 2 H, H3′, OH), 6.23 (dd, *J* = 2.3 Hz, *J_1′,2′b_* = 8.2 Hz, 1 H, H1′), 6.86–6.89 (m, 4 H, H^ar,DMTr^), 7.20–7.24 (m, 1 H, H^ar,DMTr^), 7.28–7.32 (m, 2 H, H^ar,DMTr^), 7.37–7.42 (m, 4 H, H^ar,DMTr^), 7.52–7.54 (m, 2 H, H^ar,DMTr^), 7.73 (d, *J_CH3T,6_* = 1.1 Hz, 1 H, H6) ppm. ^13^C NMR (100 MHz, acetone-*d*_6_): δ = 13.5 (CH_3_^T^), 27.6 (N-CH_3_), 42.2 (C2′), 55.5 (OCH_3_), 63.7 (C5′), 70.8 (C3′), 84.7 (C4′), 86.7 (C1′), 87.0 (qC, DMTr), 108.7 (C5), 113.8 (DMTr), 113.9 (DMTr), 127.5 (DMTr), 128.5 (DMTr), 129.0 (DMTr), 130.98 (DMTr), 131.01 (DMTr), 136.1 (C6), 136.8 (qC, DMTr), 137.0 (qC, DMTr), 146.2 (qC, DMTr), 151.9 (C2), 159.6 (qC, DMTr), 164.0 (C4) ppm. HRMS (APCI^+^): *m/z* calc. 559.2439 [M + H]^+^, found: 559.2435.

**3′-Amino-3′-deoxythymidine (25):** Amine **25** was prepared according to a published procedure [41]. AZT (**24**) (1.00 g, 3.74 mmol) was dissolved in MeOH (35 mL) and added to a dried flask containing Pd/C (10%, 150 mg) and H_2_ was bubbled through the suspension while stirring at r.t. for 2 h. After this time, TLC analysis (EtOAc-MeOH, 4:1) showed the complete consumption of starting material, (R_f_ = 0.8) and the formation of the product (R_f_ = 0.1). The reaction mixture was filtered through celite and the filtrate was concentrated to afford the desired product **25** as a white foam (893 mg, 99%). ν_max_/cm^−1^ (neat) 3363 (OH, NH), 3338 (OH, NH), 3290 (OH, NH), 2955 (CH), 2902 (CH), 2760 (CH), 1706 (C=O), 1663 (C=O), 1607 (C=O), 1477 (CH), 1441, 1421, 1370, 1360, 1331, 1295, 1245, 1207, 1092 (C-O), 1048, 1029, 955, 916, 869, 762. ^1^H NMR (400 MHz, DMSO-*d*_6_): δ = 1.76 (d, *J* = 1.1 Hz, 3 H, CH_3_^T^), 1.98 (dt, *J_1′,2′a_* = 6.7 Hz, *J_2′a,3′_* = 6.7 Hz, *J_2′a,2′b_* = 13.1 Hz, 1 H, H2′a), 2.08 (ddd, *J_1′,2′b_* = 5.2 Hz, *J_2′b,3′_* = 7.1 Hz, *J_2′a,2′b_* = 13.1 Hz, 1 H, H2′b), 3.37–3.42 (m, 1 H, H3′), 3.50–3.53 (m, 1 H, H4′), 3.56 (dd, *J_4′,5′a_* = 3.8 Hz, *J_5′a,5′b_* = 11.8 Hz, 1 H, H5′a), 3.65 (dd, *J_4′,5′b_* = 3.1 Hz, *J_5′a,5′b_* = 11.8 Hz, 1 H, H5′b), 4.98 (bs, 1 H, OH), 6.08 (dd, *J_1′,2′b_* = 5.2 Hz, *J_1′,2′a_* = 6.7 Hz, 1 H, H1′), 7.75 (app. d, *J* = 1.1 Hz, 1 H, H6) ppm. ^13^C NMR (100 MHz, DMSO-*d*_6_): δ = 12.3 (CH_3_^T^), 40.7 (C2′), 50.8 (C3′), 60.8 (C5′), 83.5 (C1′), 87.6 (C4′), 108.9 (C5), 136.3 (C6), 150.4 (C2), 163.8 (C4) ppm. HRMS (ESI^+^): *m/z* calc. 264.0955 [M + Na]^+^, found: 264.0956. Spectroscopic data are in agreement with the literature [42].

***N-(*3′-Deoxythymidin-3′-yl) amido methyl malonamide (26):** EDC·HCl (318 mg, 1.66 mmol) was suspended in dry DMF (8 mL) under argon and cooled to 0 °C. Monomethyl potassium malonate (285 mg, 1.66 mmol) was added and the suspension was stirred at 0 °C for 1 h. After this time, aminothymidine **25** (400 mg, 1.66 mmol) was added followed by 4-(dimethylamino)pyridine (20 mg, 0.16 mmol) the reaction was slowly warmed to r.t. and stirred for 26 h. After this time, TLC analysis (CH_2_Cl_2_-MeOH, 4:1) showed the consumption of amine starting material, (R_f_ = 0.1) and the formation of the product (R_f_ = 0.5). The reaction mixture was concentrated to an oil and purification by flash column chromatography afforded the desired product **26** as a colourless solid (190 mg, 34%); mp 153–154 °C. ν_max_/cm^−1^ (neat) 3316 (OH, NH), 3247 (OH, NH), 3059 (CH), 2933 (CH), 1702 (C=O), 1655 (C=O), 1643 (C=O), 1551, 1476 (CH), 1438, 1272, 1101 (C-O), 1065 (C-O), 969, 768. ^1^H NMR (400 MHz, DMSO-*d*_6_): δ = 1.78 (d, *J* = 1.0 Hz, 3 H, CH_3_^T^), 2.05–2.11 (m, 1 H, H2′a), 2.19–2.27 (m, 1 H, H2′b), 3.25 (s, 2 H, COCH_2_CO), 3.52–3.57 (m, 1 H, H5′a), 3.61–3.66 (m, 4 H, H5′b, CO_2_CH_3_), 3.75–3.78 (m, 1 H, H4′), 4.28–4.34 (m, 1 H, H3′), 5.09 (app. t, *J* = 5.2 Hz, 1 H, OH), 6.19 (app. t, *J* = 6.7 Hz, 1 H, H1′), 7.76 (app. d, *J* = 1.0 Hz, 1 H, H6), 8.57 (d, *J* = 7.2 Hz, 1 H, CONH), 11.29 (s, 1 H, NH^T^) ppm. ^13^C NMR (100 MHz, DMSO-*d*_6_): δ = 12.3 (CH_3_^T^), 36.8 (C2′), 42.2 (COCH_2_CO), 49.5 (C3′), 51.9 (OCH_3_), 61.4 (C5′), 83.5 (C1′), 85.1 (C4′), 109.5 (C5), 136.1 (C6), 150.5 (C2), 163.8 (C4), 165.1 (CONH), 168.2 (CO_2_CH_3_) ppm. HRMS (ESI^+^): *m/z* calc. 364.1115 [M + Na]^+^, found: 364.1121.

***N-(*3′-Deoxythymidin-3′-yl) amido sodium malonamide (27):** KOH (25 mg, 446 µmol) was dissolved in MeOH (1 mL) and cooled to 0 °C. Ester **26** (50 mg, 147 µmol) was added, the solution was slowly warmed to r.t. and stirred for 5.5 h. After this time, TLC analysis (CH_2_Cl_2_-MeOH, 4:1) showed the consumption of starting material (R_f_ = 0.8) and the formation of a product (R_f_ = 0.0). The reaction mixture was eluted through Diaion resin WT01S(H) (H form) to remove excess KOH, then eluted through Diaion resin WT01S(H) (Na form) and concentrated to a white solid. After purification by flash column chromatography (CH_2_Cl_2_-MeOH, 4:1→MeOH), the residue was taken up in MeOH and eluted through Diaion resin WT01S(H) (Na form). The desired product **27** was obtained as a white foam (42 mg, 82%); mp 202–205 °C dec. ν_max_/cm^−1^ (neat) 3431 (OH, NH), 3263 (OH, NH), 3071 (CH), 2954 (CH), 2926 (CH), 1642 (C=O), 1593 (C=O), 1473 (CH), 1441, 1379 (C=O), 1318, 1302, 1276, 1233, 1102 (C-O), 1073, 978, 927, 886, 785. ^1^H NMR (600 MHz, DMSO-*d*_6_): δ = 1.75 (d, *J* = 0.7 Hz, 3 H, CH_3_^T^), 2.05–2.09 (m, 1 H, H2′a), 2.19 (ddd, *J_1′,2′b_* = 6.5 Hz, *J* = 8.0 Hz, *J* = 13.3 Hz, 1 H, H2′b), 2.70 (s, 2 H, COCH_2_CO), 3.53 (dd, *J* = 4.2 Hz, *J_5′a,5′b_* = 11.9 Hz, 1 H, H5′a), 3.62 (dd, *J* = 2.9 Hz, *J_5′a,5′b_* = 11.9 Hz, 1 H, H5′b), 3.70–3.73 (m, 1 H, H4′), 4.27–4.31 (m, 1 H, H3′), 5.06 (bs, 1 H, 5′-OH), 6.15 (app. t, *J* = 6.5 Hz, 1 H, H1′), 7.63 (app. s, 1 H, H6), 9.95–9.97 (m, 1 H, CONH) ppm. ^13^C NMR (150 MHz, DMSO-*d*_6_): δ = 12.7 (CH_3_^T^), 37.4 (C2′), 44.8 (COCH_2_CO), 48.5 (C3′), 61.4 (C5′), 83.5 (C1′), 85.0 (C4′), 109.3 (C5), 135.6 (C6), 152.4 (C2), 166.4 (C4), 170.0 (CONH), 170.4 (CO_2_Na) ppm. HRMS (ESI^+^): *m/z* calc. 350.0959 [M + H]^+^, found: 350.0967.

***N-(*3′-Deoxythymidin-3′-yl) amido *N*-hydroxymalonamide (28):** Hydroxylamine hydrochloride (407 mg, 5.86 mmol) was suspended in MeOH (6 mL) and KOH (411 mg, 7.33 mmol) was added. The suspension was warmed to 40 °C to aid dissolution and the precipitate was removed by filtration. Ester **26** (100 mg, 293 µmol) was dissolved in the filtrate and the reaction was stirred at r.t. for 2.5 h, during which a white precipitate formed. After this time, TLC analysis (CH_2_Cl_2_-MeOH, 4:1) showed the complete consumption of starting material (R_f_ = 0.7) and the formation of the product (R_f_ = 0.1). The reaction mixture was cooled to 0 °C and neutralised to pH 7 using aq. HCl (1 M). The white precipitate was collected by vacuum filtration to afford the desired product **28** as a white amorphous solid (70 mg, 70%); mp 166–170 °C dec. ν_max_/cm^−1^ (neat) 3253 (OH, NH), 3065 (CH), 2955 (CH), 2826 (CH), 1643 (C=O), 1599 (C=O),1552, 1431, 1274, 1101, 1072, 971, 883, 762. ^1^H NMR (600 MHz, DMSO-*d*_6_): δ = 1.77 (s, 3 H, CH_3_^T^), 2.08–2.12 (m, 1 H, H2′a), 2.18–2.23 (m, 1 H, H2′b), 2.83 (s, 2 H, COCH_2_CO), 3.54 (dd, *J* = 3.9 Hz, *J_5′a,5′b_* = 12.0 Hz, 1 H, H5′a), 3.62 (dd, *J* = 2.4 Hz, *J_5′a,5′b_* = 12.0 Hz, 1 H, H5′b), 3.75–3.77 (m, 1 H, H4′), 4.28–4.32 (m, 1 H, H3′), 6.18 (app. t, *J* = 6.6 Hz, 1 H, H1′), 7.74 (s, 1 H, H6), 8.78 (bs, 1 H, NHO-C) ppm. ^13^C NMR (150 MHz, DMSO-*d*_6_): δ = 12.3 (CH_3_^T^), 37.0 (C2′), 40.8 (COCH_2_CO), 49.1 (C3′), 61.4 (C5′), 83.5 (C1′), 85.0 (C4′), 109.4 (C5), 136.1 (C6), 150.8 (C2), 163.1 (CONHOH), 164.3 (C4), 167.6 (CONHC) ppm. HRMS (ESI^−^): *m/z* calc. 341.1103 [M − H]^−^, found: 341.1109.

***C*-(3′-*O*-(*tert*-Butyldimethylsilyl-5′-deoxy-*N^3^*-hydroxymethyl)thymidin-5′-yl) malonic acid monomethyl ester (29):** Malonate ester **6** (550 mg, 825 µmol) was dissolved in MeOH (20 mL) and added to a dried flask containing Pd/C (10%, 104 mg) and H_2_ was bubbled through the suspension while stirring at r.t. for 1.5 h. After this time, TLC analysis showed the complete consumption of starting material (CH_2_Cl_2_-EtOAc, 4:1, R_f_ = 0.8) and the formation of the product (CH_2_Cl_2_-MeOH, 4:1, R_f_ = 0.5). The reaction mixture was filtered through celite and the filtrate was concentrated to afford the desired product **29** as a yellow foam (380 mg, 95%). Compound **29** was isolated as a 1:1 mixture of interconverting diastereoisomers. ν_max_/cm^−1^ (neat) 3188 (OH), 2955 (CH), 2929 (CH), 2858 (CH), 1705 (C=O), 1473 (CH), 1437, 1275, 1048 (C-O), 837, 779. ^1^H NMR (600 MHz, acetone-*d*_6_): δ = 0.13–0.14 (m, 12 H, 4 × CH_3_^TBDMS^), 0.918–0.922 (2 × s, 18 H, *t*-Bu^TBDMS^), 1.86 (s, 3 H, CH_3_^T^), 1.88 (s, 3 H, CH_3_^T^), 2.15–2.25 (m, 4 H, H2′a, H5′a), 2.33–2.41 (m, 4 H, H2′b, H5′a), 3.60–3.63 (m, 2 H, H6′), 3.67 (s, 3 H, CO_2_CH_3_), 3.70 (s, 3 H, CO_2_CH_3_), 3.83–3.86 (m, 1 H, H4′), 3.88–3.91 (m, 1 H, H4′), 4.38 (m, 2 H, H3′), 5.38 (s, 4 H, N-CH_2_-O), 6.27–6.30 (m, 2 H, H1′), 7.47 (s, 1 H, H6), 7.49 (s, 1 H, H6) ppm. ^13^C NMR (151 MHz, acetone-*d*_6_): δ = −4.7 (CH_3_^TBDMS^), −4.5 (CH_3_^TBDMS^), 13.0 (CH_3_^T^), 18.48 (qC, *t*-Bu^TBDMS^), 18.49 (qC, *t*-Bu^TBDMS^), 26.1 (*t*-Bu^TBDMS^), 33.3 (C5′), 33.4 (C5′), 40.3 (C2′), 40.4 (C2′), 49.3 (C6′), 49.5 (C6′), 52.60 (CO_2_CH_3_), 52.64 (CO_2_CH_3_), 65.4 (N-CH_2_-O), 76.0 (C3′), 76.1 (C3′), 85.1 (C4′), 85.4 (C4′), 86.1 (C1′), 110.5 (C5), 110.6 (C5), 135.9 (C6), 151.5 (C2), 163.6 (C4), 170.3 (CO_2_CH_3_), 170.4 (CO_2_H), 170.90 (CO_2_CH_3_), 170.93 (CO_2_H) ppm. HRMS (ESI^+^): *m/z* calc. 509.1926 [M + Na]^+^, found: 509.1928.

**3′-Amino-3′-deoxy-5′-*O*-(4,4′-dimethoxytrityl)thymidine (31):** Amine **31** was prepared according to a published procedure [36]. DMT-protected AZT **30** (3.00 g, 5.27 mmol) was suspended in *i*-PrOH (100 mL) and NaBH_4_ (1.00 g, 26.43 mmol) was added. The suspension was heated to reflux and stirred for 17 h. After this time, TLC analysis (CH_2_Cl_2_-MeOH, 9:1) showed the complete consumption of starting material (R_f_ = 0.7) and the formation of the product (R_f_ = 0.4). The reaction mixture was diluted with CH_2_Cl_2_ (200 mL), washed with H_2_O (200 mL) and brine (200 mL), dried over MgSO_4_, filtered and concentrated to afford the desired product **31** as a white foam (2.73 g, 95%) which was used without further purification. ν_max_/cm^−1^ (neat) 3164 (NH), 3057 (CH), 2930 (CH), 2836, 1682 (C=O), 1607 (C=O), 1508, 1463 (CH), 1445, 1299, 1247, 1175, 1031 (C-O), 826, 771, 756, 727, 701. ^1^H NMR (400 MHz, acetone-*d*_6_): δ = 1.55 (s, 3 H, CH_3_^T^), 2.29 (app. dt, *J* = 6.7 Hz, *J* = 13.2 Hz, 1 H, H2′a), 2.42 (ddd, *J_1′,2′b_* = 5.5 Hz, *J_2′b,3′_* = 7.5 Hz, *J_2′a,2′b_* = 13.2 Hz, 1 H, 2′-b), 3.24 (dd, *J_4′,5′a_* = 3.7 Hz, *J_5′a,5′b_* = 10.6 Hz, 1 H, H5′a), 3.43 (dd, *J_4′,5′b_* = 2.9 Hz, *J_5′a,5′b_* = 10.6 Hz, 1 H, H5′b), 3.79 (s, 6 H, 2 × OCH_3_), 4.03–4.08 (m, 1 H, H4′), 4.40–4.45 (m, 1 H, H3′), 6.31 (dd, *J_1′,2′b_* = 5.5 Hz, *J_1′,2′a_* = 6.7 Hz, 1 H, H1′), 6.89–6.91 (m, 4 H, H^ar,DMTr^), 7.22–7.26 (m, 1 H, H^ar,DMTr^), 7.31–7.39 (m, 6 H, H^ar,DMTr^), 7.49–7.51 (m, 2 H, H^ar,DMTr^), 7.74 (s, 1 H, H6), 10.02 (bs, 1 H, NH) ppm. ^13^C NMR (151 MHz, acetone-*d*_6_): δ = 12.4 (CH_3_^T^), 39.8 (C2′), 55.5 (OCH_3_), 60.0 (C3′), 64.0 (C5′), 85.7 (C1′), 85.9 (C4′), 87.3 (qC, DMTr), 110.6 (C5), 113.97 (DMTr), 114.03 (DMTr), 127.7 (DMTr), 128.7 (DMTr), 129.0 (DMTr), 130.97 (DMTr), 130.99 (DMTr), 131.01 (DMTr), 136.6 (C6), 136.7 (qC, DMTr), 146.0 (qC, DMTr), 151.2 (C2), 159.7 (qC, DMTr), 164.3 (C4) ppm. HRMS (ESI^−^): *m/z* calc. 542.2297 [M − H]^−^, found: 542.2305. Spectroscopic data are in agreement with the literature [43].

***C*-(3′-*O*-(*tert*-Butyldimethylsilyl)-5′-deoxythymidin-5′-yl)-*N*-(3′-deoxy-5′-*O*-(4,4′-dimethoxytrityl)thymidin-3′-yl) amido methyl malonate (32):** Carboxylic acid **29** (375 mg, 0.82 mmol) was dissolved in dry DMF (15 mL) under argon and cooled to 0 °C. HOAt (168 mg, 1.23 mmol) and EDC·HCl (236 mg, 1.23 mmol) were added, followed by the addition of aminothymidine **31** (469 mg, 0.86 mmol). The reaction mixture was warmed to r.t and stirred at r.t. for 18 h. After this time, TLC analysis (CH_2_Cl_2_-MeOH, 9:1) showed the consumption of starting materials (R_f_ = 0.2 and R_f_ = 0.5) and the formation of the product (R_f_ = 0.8). The reaction was diluted with EtOAc (100 mL) and washed with H_2_O (50 mL), sat. aq. NaHCO_3_ (50 mL) and brine (50 mL). The combined organic extracts were dried over MgSO_4_, filtered and concentrated. Purification by flash column chromatography (EtOAc-petroleum ether, 9:1) afforded the desired product **32** as a white solid (614 mg, 76%). Compound **32** was isolated as 4:3 mixture of interconverting diastereoisomers. ν_max_/cm^−1^ (neat) 2950 (CH), 2930 (CH), 2855 (CH), 1685 (C=O), 1608 (C=O), 1508, 1465 (CH), 1249, 1175, 1032 (C-O), 831, 777. Major diastereoisomer: ^1^H NMR (600 MHz, acetone-*d*_6_): δ = 0.13–0.14 (2 × s, 6 H, CH_3_^TBDMS^), 0.92 (s, 9 H, *t*-Bu^TBDMS^), 1.43 (s, 3 H, 5′-CH_3_^T^), 1.84 (s, 3 H, 3′-CH_3_^T^), 2.09–2.20 (m, 2 H, 3′-H2′a, 3′-H5′a), 2.30–2.41 (m, 4 H, 5′-H2′a, 5′-H2′b, 3′-H2′b, 3′-H5′b), 3.37–3.45 (m, 2 H, 5′-H5′a, 5′-H5′b), 3.50–3.54 (m, 1 H, 3′-H6′), 3.56 (s, 3 H, CO_2_CH_3_), 3.79 (s, 6 H, 2 × OCH_3_), 3.88–3.91 (m, 1 H, 3′-H4′), 4.04–4.05 (m, 1 H, 5′-H4′), 4.33–4.36 (m, 1 H, 3′-H3′), 4.74–4.82 (m, 1 H, 5′-H3′), 6.18 (app. t, *J* = 7.0 Hz, 1 H, 3′-H1′), 6.27 (app. t, *J* = 6.4 Hz, 1 H, 5′-H1′), 6.88–6.91 (m, 4 H, H^ar^), 7.23–7.26 (m, 1 H, H^ar^), 7.31–7.39 (m, 6 H, H^ar^), 7.45 (s, 1 H, 3′-H6), 7.49–7.52 (m, 2 H, H^ar^), 7.61 (s, 1 H, 5′-H6), 7.86 (d, *J_3′,NH_* = 7.8 Hz, 1 H, CONH), 9.95–9.98 (m, 2 H, 5′-NH^T^, 3′-NH^T^) ppm. ^13^C NMR (151 MHz, acetone-*d*_6_): δ = −4.63 (CH_3_^TBDMS^), −4.55 (CH_3_^TBDMS^), 12.15 (5′-CH_3_^T^), 12.54 (3′-CH_3_^T^), 18.54 (qC, *t*-Bu^TBDMS^), 26.16 (*t*-Bu^TBDMS^), 33.4 (3′-C5′), 38.30 (5′-C2′), 39.8 (3′-C2′), 50.6 (5′-C3′), 50.7 (3′-C6′), 52.5 (CO_2_CH_3_), 55.53 (OCH_3_^DMT^), 64.1 (5′-C5′), 76.6 (3′-C3′), 84.5 (5′-C4′), 84.8 (5′-C1′), 86.3 (3′-C4′), 86.6 (3′-C1′), 87.37 (qC, DMTr), 110.9 (3′-C5), 111.12 (5′-C5), 113.99 (CH, DMTr), 114.00 (CH, DMTr), 127.69 (CH, DMTr), 128.72 (CH, DMTr), 129.1 (CH, DMTr), 131.09 (CH, DMTr), 131.11 (CH, DMTr), 136.2 (5′-C6, 136.5 (qC, DMTr), 136.7 (qC, DMTr), 137.6 (3′-C6), 145.95 (qC, DMTr), 151.3 (5′-C2, 3′-C2), 159.70 (qC, DMTr), 159.73 (qC, DMTr), 164.17 (5′-C4), 164.25 (3′-C4), 169.3 (CONH), 170.6 (CO_2_) ppm. Minor diastereoisomer: ^1^H NMR (600 MHz, acetone-*d*_6_): δ = 0.09 (s, 3 H, CH_3_^TBDMS^), 0.11 (s, 3 H, CH_3_^TBDMS^), 0.88 (s, 9 H, *t*-Bu^TBDMS^), 1.44 (s, 3 H, 5′-CH_3_^T^), 1.82 (s, 3 H, 3′-CH_3_^T^), 2.09–2.20 (m, 2 H, 3′-H2′a, 3′-H5′a), 2.30–2.41 (m, 3 H, 5′-H2′a, 3′-H2′b, 3′-H5′b), 2.48–2.53 (m, 1 H, 5′-H2′b), 3.37–3.45 (m, 2 H, 5′-H5′a, 5′-H5′b), 3.50–3.54 (m, 1 H, 3′-H6′), 3.66 (s, 3 H, CO_2_CH_3_), 3.74–3.76 (m, 1 H, 3′-H4′), 3.79 (s, 6 H, 2 × OCH_3_), 4.06–4.08 (m, 1 H, 5′-H4′), 4.33–4.36 (m, 1 H, 3′-H3′), 4.74–4.82 (m, 1 H, 5′-H3′), 6.22 (app. t, *J* = 6.9 Hz, 1 H, 3′-H1′), 6.32 (app. t, *J* = 6.8 Hz, 1 H, 5′-H1′), 6.88–6.91 (m, 4 H, H^ar^), 7.23–7.26 (m, 1 H, H^ar^), 7.31–7.39 (m, 6 H, H^ar^), 7.42 (s, 1 H, 3′-H6), 7.49–7.52 (m, 2 H, H^ar^), 7.65 (s, 1 H, 5′-H6), 7.91 (d, *J_3′,NH_* = 7.5 Hz, 1 H, CONH), 9.95–9.98 (m, 2 H, 5′-NH^T^, 3′-NH^T^) ppm. ^13^C NMR (151 MHz, acetone-*d*_6_): δ = −4.58 (CH_3_^TBDMS^), −4.51 (CH_3_^TBDMS^), 12.13 (5′-CH_3_^T^), 12.46 (3′-CH_3_^T^), 18.53 (qC, *t*-Bu^TBDMS^), 26.17 (*t*-Bu^TBDMS^), 33.3 (3′-C5′), 38.32 (5′-C2′), 40.1 (3′-C2′), 50.0 (3′-C6′), 51.3 (5′-C3′), 52.6 (CO_2_CH_3_), 55.55 (OCH_3_^DMT^), 64.5 (5′-C5′), 76.3 (3′-C3′), 84.6 (5′-C4′), 84.9 (5′-C1′), 85.0 (3′-C4′), 85.5 (3′-C1′), 87.42 (qC, DMTr), 111.08 (3′-C5, 5′-C5), 114.01 (CH, DMTr), 114.02 (CH, DMTr), 127.73 (CH, DMTr), 128.74 (CH, DMTr), 129.1 (CH, DMTr), 131.06 (CH, DMTr), 131.07 (CH, DMTr), 136.3 (5′-C6), 136.6 (qC, DMTr), 136.8 (qC, DMTr), 136.9 (3′-C6), 145.91 (qC, DMTr), 151.3 (3′-C2, 5′-C2), 159.72 (qC, DMTr), 159.74 (qC, DMTr), 164.18 (3′-C4), 164.20 (5′-C4), 168.4 (CONH), 171.2 (CO_2_) ppm. HRMS (APCI^+^): *m/z* calc. 1004.4084 [M + Na]^+^, found: 1004.4068.

***N*-(3′-Deoxy-5′-*O*-(4,4′-dimethoxytrityl)thymidin-3′-yl)-*C*-(5′-deoxythymidin-5′-yl) amido methyl malonate (33):** Protected dimer **32** (580 mg, 0.59 mmol) was dissolved in THF (8 mL). Tetra-*n*-butylammonium fluoride trihydrate (316 mg, 1.06 mmol) was added and the solution was stirred at r.t. for 24 h. After this time, TLC analysis (EtOAc-MeOH, 9:1) showed the complete consumption of the starting material (R_f_ = 0.7) and product formation as a mixture of diastereomers (R_f_ = 0.3 and R_f_ = 0.4). The suspension was concentrated and purification by flash column chromatography (EtOAc-MeOH, 19:1→9:1) afforded the desired product **33** as a white solid (472 mg, 92%). Compound **33** was isolated as a 3:1 mixture of interconverting diastereoisomers. ν_max_/cm^−1^ (neat) 3310 (OH, NH), 3073 (CH), 2950 (CH), 2930 (CH), 2839 (CH), 1655 (C=O), 1607 (C=O), 1508, 1466 (CH), 1248, 1175, 1031 (C-O), 828. Major diastereoisomer: ^1^H NMR (600 MHz, acetone-*d*_6_): δ = 1.43 (d, *J_CH3T,6_* = 1.0 Hz, 3 H, 5′-CH_3_^T^), 1.86 (d, *J_CH3T,6_* = 1.0 Hz, 3 H, 3′-CH_3_^T^), 2.10–2.23 (m, 2 H, 3′H2′a, 3′-H5′a), 2.27–2.38 (m, 4 H, 5′-H2′a, 5′-H2′b, 3′-H2′b, 3′-H5′b), 3.37–3.46 (m, 2 H, 5′-H5′a, 5′-H5′b), 3.52–3.56 (m, 4 H, 3′-H6′, CO_2_CH_3_), 3.78 (s, 6 H, 2 × OCH_3_), 3.90 (app. dt, *J* = 3.6 Hz, *J* = 10.2 Hz, 1 H, 3′-H4′), 4.05–4.07 (m, 1 H, 5′-H4′), 4.25–4.28 (m, 1 H, 3′-H3′), 4.57 (d, *J_3′,OH_* = 3.3 Hz, 1 H, OH), 4.75–4.80 (m, 1 H, 5′-H3′), 6.21 (dd, *J* = 6.2 Hz, *J* = 7.6 Hz, 1 H, 3′-H1′), 6.27 (app. t, *J* = 6.3 Hz, 1 H, 5′-H1′), 6.88–6.91 (m, 4 H, H^ar^), 7.22–7.25 (m, 1 H, H^ar^), 7.30–7.38 (m, 6 H, H^ar^), 7.46 (app. d, *J* = 1.0 Hz, 1 H, 3′-H6), 7.49–7.52 (m, 2 H, H^ar^), 7.63 (app. d, *J* = 1.0 Hz, 1 H, 5′-H6), 7.90 (d, *J_3′,NH_* = 7.9 Hz, 1 H, CONH), 10.09–10.13 (m, 2 H, 5′-NH^T^, 3′-NH^T^) ppm. ^13^C NMR (151 MHz, acetone-*d*_6_): δ = 12.2 (5′-CH_3_^T^), 12.54 (3′-CH_3_^T^), 33.6 (3′-C5′), 38.4 (5′-C2′), 39.5 (3′-C2′), 50.5 (5′-C3′), 50.7 (3′-C6′), 52.5 (CO_2_CH_3_), 55.53 (OCH_3_^DMT^), 64.0 (5′-C5′), 75.3 (3′-C3′), 84.4 (5′-C4′), 84.93 (5′-C1′), 86.0 (3′-C4′), 86.2 (3′-C1′), 87.36 (qC, DMTr), 111.0 (3′-C5), 111.2 (5′-C5), 113.98 (CH, DMTr), 114.00 (CH, DMTr), 114.02 (CH, DMTr), 127.68 (CH, DMTr), 128.71 (CH, DMTr), 129.1 (CH, DMTr), 131.07 (CH, DMTr), 131.09 (CH, DMTr), 136.3 (5′-C6), 136.5 (qC, DMTr), 136.63 (qC, DMTr), 137.6 (3′-C6), 145.92 (qC, DMTr), 151.40 (5′-C2), 151.41 (3′-C2), 159.66 (qC, DMTr), 159.70 (qC, DMTr), 164.4 (5′-C4), 164.5 (3′-C4), 169.5 (CONH), 170.7 (CO_2_) ppm. Minor diastereoisomer: ^1^H NMR (600 MHz, acetone-*d*_6_): δ = 1.44 (d, *J_CH3T,6_* = 1.0 Hz, 3 H, 5′-CH_3_^T^), 1.83 (d, *J_CH3T,6_* = 1.0 Hz, 3 H, 3′-CH_3_^T^), 2.10–2.23 (m, 2 H, 3′-H2′a, 3′-H5′a), 2.27–2.33 (m, 2 H, 3′-H2′b, 3′-H5′b), 2.39–2.42 (m, 1 H, 5′-H2′a), 2.49–2.54 (m, 1 H, 5′-H2′b), 3.37–3.46 (m, 2 H, 5′-H5′a, 5′-H5′b), 3.52–3.56 (m, 1 H, 3′-H6′), 3.65 (s, 3 H, CO_2_CH_3_), 3.76–3.78 (m, 7 H, 3′-H4′, 2 × OCH_3_), 4.08–4.10 (m, 1 H, 5′-H4′), 4.25–4.28 (m, 1 H, 3′-H3′), 4.51 (d, *J_3′,OH_* = 4.2 Hz, 1 H, OH), 4.75–4.80 (m, 1 H, 5′-H3′), 6.24 (app. t, *J* = 6.9 Hz, 1 H, 3′-H1′), 6.33 (app. t, *J* = 6.6 Hz, 1 H, 5′-H1′), 6.88–6.91 (m, 4 H, H^ar^), 7.22–7.25 (m, 1 H, H^ar^), 7.30–7.38 (m, 6 H, H^ar^), 7.42 (app. d, *J* = 1.0 Hz, 1 H, 3′-H6), 7.49–7.52 (m, 2 H, H^ar^), 7.66 (app. d, *J* = 1.0 Hz, 1 H, 5′-H6), 7.98 (d, *J_3′,NH_* = 7.5 Hz, 1 H, CONH), 10.09–10.13 (m, 2 H, 5′-NH^T^, 3′-NH^T^) ppm. ^13^C NMR (151 MHz, acetone-*d*_6_): δ = 12.1 (5′-CH_3_^T^), 12.47 (3′-CH_3_^T^), 33.6 (3′-C5′), 38.3 (5′-C2′), 39.7 (3′-C2′), 50.0 (3′-C6′), 51.2 (5′-C3′), 52.6 (CO_2_CH_3_), 55.55 (OCH_3_^DMT^), 64.4 (5′-C5′), 75.1 (3′-C3′), 84.6 (5′-C4′), 84.87 (3′-C4′), 84.94 (5′-C1′), 85.3 (3′-C1′), 87.40 (qC, DMTr), 111.1 (5′-C5, 3′-C5), 113.6 (CH, DMTr), 113.98 (CH, DMTr), 114.00 (CH, DMTr), 114.02 (CH, DMTr), 127.72 (CH, DMTr), 128.73 (CH, DMTr), 129.1 (CH, DMTr), 131.1 (CH, DMTr), 136.44 (5′-C6), 136.55 (qC, DMTr), 136.66 (qC, DMTr), 136.9 (3′-C6), 145.93 (qC, DMTr), 151.37 (3′-C2), 151.40 (5′-C2), 159.68 (qC, DMTr), 159.71 (qC, DMTr), 164.4 (5′-C4, 3′-C4), 168.7 (CONH), 171.2 (CO_2_) ppm. HRMS (APCI^−^): *m/z* calc. 866.3254 [M − H]^−^, found: 866.3262.

***N*-(3′-Deoxythymidin-3′-yl)-*C*-(5′-deoxythymidin-5′-yl) amido methyl malonate (34):** Protected dimer **33** (100 mg, 115 µmol) was dissolved in CH_2_Cl_2_ (980 µL). Triethylsilane (184 µL, 1.15 mmol) and trifluoroacetic acid (20 µL, 261 µmol) were added and the solution was stirred at r.t. The solution turned bright red, followed by the formation of a white precipitate and loss of the red colour after 10 min. After this time, TLC analysis (EtOAc-MeOH, 4:1) showed the complete consumption of starting material (R_f_ = 0.6) and the formation of the product (R_f_ = 0.4). The supernatant was removed and the precipitated product was washed with toluene (2 × 1 mL). The washings and supernatant were combined and concentrated, and the resulting white solid was washed with CH_2_Cl_2_ (3 × 5 mL) to remove impurities. The precipitates were combined to afford the desired product **34** as a white amorphous solid (60 mg, 92%). The product **34** was isolated as a 3:1 mixture of interconverting diastereomers. ν_max_/cm^−1^ (neat) 3398 (OH, NH), 3261 (OH, NH), 3174 (OH, NH), 3050 (CH), 2955 (CH), 2819 (CH), 1742 (C=O), 1677 (C=O), 1655 (C=O), 1638 (C=O), 1547, 1473 (CH), 1369, 1273, 1203, 1097 (C-O), 1047 (C-O), 988. Major diastereoisomer: ^1^H NMR (400 MHz, DMSO-*d*_6_): δ = 1.77 (bs, 3 H, 5′-CH_3_^T^), 1.79 (bs, 3 H, 3′-CH_3_^T^), 1.88–1.96 (m, 1 H, 3′-H5′a), 1.99–2.23 (m, 5 H, 5′-H2′a, 5′-H2′b, 3′-H2′a, 3′-H2′b, 3′-H5′b), 3.42–3.45 (m, 1 H, 3′-H6′), 3.49–3.53 (m, 1 H, 5′-H5′a), 3.59–3.60 (m, 1 H, 5′-H5′b), 3.63 (s, 3 H, CO_2_CH_3_), 3.66–3.71 (m, 1 H, 3′-H4′), 3.73–3.76 (m, 1 H, 5′-H4′), 4.04–4.09 (m, 1 H, 3′-H3′), 4.23–4.29 (m, 1 H, 5′-H3′), 5.29 (bs, 2 H, 5′-OH, 3′-OH), 6.10 (app t, *J* = 7.0 Hz, 1 H, 3′-H1′), 6.19 (app. t, *J* = 6.7 Hz, 1 H, 5′-H1′), 7.38 (app. d, *J* = 0.9 Hz, 1 H, 3′-H6), 7.73 (app. d, *J* = 0.9 Hz, 1 H, 5′-H6), 8.63 (d, *J_3′,NH_* = 7.4 Hz, 1 H, CONH), 11.25–11.27 (2 × s, 2 H, 5′-NH^T^, 3′-NH^T^) ppm. ^13^C NMR (100 MHz, DMSO-*d*_6_): δ = 12.1 (3′-CH_3_^T^), 12.3 (5′-CH_3_^T^), 32.37 (3′-C5′), 36.5 (5′-C2′), 38.2 (3′-C2′), 49.0 (3′-C6′), 49.5 (5′-C3′), 52.1 (CO_2_CH_3_), 61.3 (5′-C5′), 73.3 (3′-C3′), 83.49 (5′-C1′), 83.69 (3′-C1′), 84.1 (3′-C4′), 84.9 (5′-C4′), 109.5 (5′-C5), 109.7 (3′-C5), 136.1 (5′-C6), 136.3 (3′-C6), 150.4 (5′-C2, 3′-C2), 163.7 (5′-C4, 3′-C4), 168.0 (CONH), 169.9 (CO_2_) ppm. Minor diastereoisomer: ^1^H NMR (400 MHz, DMSO-*d*_6_): δ = 1.78 (d, *J_CH3T,6_* = 1.1 Hz, 3 H, 5′-CH_3_^T^), 1.80 (d, *J_CH3T,6_* = 1.1 Hz, 3 H, 3′-CH_3_^T^), 1.97–2.25 (m, 6 H, 5′-H2′a, 5′-H2′b, 3′-H2′a, 3′-H2′b, 3′-H5′a, 3′-H5′b), 3.41–3.45 (m, 1 H, 3′-H6′), 3.52–3.58 (m, 2 H, 5′-H5′a, 3′-H4′), 3.61–3.66 (m, 4 H, 5′-H5′b, CO_2_CH_3_), 3.76–3.78 (m, 1 H, 5′-H4′), 4.04–4.09 (m, 1 H, 3′-H3′), 4.30–4.35 (m, 1 H, 5′-H3′), 5.29 (bs, 2 H, 5′-OH, 3′-OH), 6.15 (app t, *J* = 7.1 Hz, 1 H, 3′-H1′), 6.20 (app. t, *J* = 6.7 Hz, 1 H, 5′-H1′), 7.42 (app. d, *J* = 1.1 Hz, 1 H, 3′-H6), 7.76 (app. d, *J* = 1.1 Hz, 1 H, 5′-H6), 8.71 (d, *J_3′,NH_* = 7.3 Hz, 1 H, CONH), 11.29–11.30 (2 × s, 2 H, 5′-NH^T^, 3′-NH^T^) ppm. ^13^C NMR (100 MHz, DMSO-*d*_6_): δ = 12.1 (3′-CH_3_^T^), 12.3 (5′-CH_3_^T^), 32.42 (3′-C5′), 36.7 (5′-C2′), 38.0 (3′-C2′), 48.5 (3′-C6′), 49.7 (5′-C3′), 52.1 (CO_2_CH_3_), 61.5 (5′-C5′), 73.3 (3′-C3′), 83.47 (3′-C4′), 83.53 (5′-C1′), 83.7 (3′-C1′), 85.0 (5′-C4′), 109.5 (5′-C5), 109.8 (3′-C5), 136.1 (5′-C6), 136.2 (3′-C6), 150.4 (5′-C2), 150.5 (3′-C2), 163.7 (5′-C4, 3′-C4), 167.5 (CONH), 170.3 (CO_2_) ppm. HRMS (ESI^+^): *m/z* calc. 588.1912 [M + Na]^+^, found: 588.1922.

***N*-(3′-Deoxythymidin-3′-yl)-*C*-(5′-deoxythymidin-5′-yl) amido sodium malonate (35):** KOH (18 mg, 320 µmol) was dissolved in MeOH (1 mL) and cooled to 0 °C. Ester **34** (30 mg, 53 µmol) was added, the solution was slowly warmed to r.t. and stirred for 7 h. After this time, TLC analysis (H_2_O-*i*-PrOH-EtOAc, 1:5:4) showed the consumption of starting material (R_f_ = 0.8) and the formation of product (R_f_ = 0.3). The reaction mixture was concentrated and eluted through Diaion resin WT01S(H) (H form) to remove excess KOH, then eluted through Diaion resin WT01S(H) (Na form) and concentrated to afford the desired product **35** as a white solid (27 mg, 89%). Compound **35** was isolated as a 1:1 mixture of interconverting diastereomers. ν_max_/cm^−1^ (neat) 3259 (OH, NH), 3063 (CH), 2925 (CH), 2820 (CH), 1655 (C=O), 1587 (C=O), 1474 (CH), 1366, 1270, 1090 (C-O), 1050 (C-O), 962, 766. ^1^H NMR (600 MHz, CD_3_OD): δ = 1.89–1.91 (m, 12 H, 4 × CH_3_^T^), 2.05–2.41 (m, 12 H, 3′-H2′a, 3′-H2′b, 3′-H5′a, 3′-H5′b, 5′-H2′a, 5′-H5′b), 3.23 (dd, *J* = 5.5 Hz, *J* = 8.0 Hz, 1 H, 3′-H6′), 3.31–3.35 (m, 1 H, 3′-H6′), 3.73–3.91 (m, 8 H, 3′-H4′, 5′-H4′, 5′-H5′a, 5′-H5′b), 4.14–4.19 (m, 2 H, 3′-H3′), 4.46 (app. q, *J* = 7.2 Hz, 1 H, 5′-H3′), 4.51 (app. q, *J* = 7.2 Hz, 1 H, 5′-H3′), 6.15–6.25 (m, 4 H, 3′-H1′, 5′-H1′), 7.48–7.50 (2 × s, 2 H, H6), 7.88–7.90 (2 × s, 2 H, H6) ppm. ^13^C NMR (151 MHz, CD_3_OD): δ = 12.45 (CH_3_^T^), 12.51 (CH_3_^T^), 12.53 (CH_3_^T^), 35.4 (3′-C5′), 35.5 (3′-C5′), 38.5 (5′-C2′), 38.8 (5′-C2′), 39.9 (3′-C2′), 40.1 (3′-C2′), 49.7 (5′-C3′), 50.0 (5′-C3′), 53.6 (3′-C6′), 55.1 (3′-C6′), 62.0 (5′-C5′), 62.3 (5′-C5′), 75.4 (3′-C3′), 75.3 (3′-C3′), 85.9 (3′-C1′, 5′-C1′), 86.0 (3′-C1′, 5′-C1′), 86.15 (3′-C4′), 86.19 (5′-C4′), 86.6 (5′-C4′), 87.5 (3′-C4′), 111.3 (C5), 111.4 (C5), 111.7 (C5), 112.0 (C5), 137.8 (C6), 138.1 (C6), 138.2 (C6), 152.39 (C2), 152.43 (C2), 152.45 (C2), 152.54 (C2), 166.5 (C4), 166.6 (C4), 166.68 (C4), 166.70 (C4), 174.3 (CONH), 174.8 (CONH), 176.8 (CO_2_), 177.1 (CO_2_) ppm. HRMS (ESI^+^): *m/z* calc. 596.1575 [M + Na]^+^, found: 596.1594.

***N*-(3′-Deoxythymidin-3′-yl)-*C*-(5′-deoxythymidin-5′-yl) amido *N*-hydroxy malonamide (36):** Hydroxylamine hydrochloride (123 mg, 1.77 mmol) was suspended in MeOH (2 mL) and KOH (124 mg, 2.21 mmol) was added. The suspension was warmed to 40 °C to aid dissolution and the precipitate was removed by filtration. Ester **34** (50 mg, 88 µmol) was dissolved in the filtrate and the reaction was stirred at r.t. for 48 h, during which a white precipitate formed. After this time, TLC analysis (H_2_O-*i*-PrOH-EtOAc, 1:5:4) showed the consumption of starting material (R_f_ = 0.8) and the formation of the product (R_f_ = 0.4). The reaction mixture was neutralised to pH 7 using aq. HCl (1 M) and concentrated. Purification by flash column chromatography (H_2_O-*i*-PrOH-EtOAc, 1:5:4) afforded the desired product **36** as a white amorphous solid (37 mg, 74%). Compound **36** was isolated as a 5:4 mixture of interconverting diastereomers. ν_max_/cm^−1^ (neat) 3409 (OH, NH), 3162 (OH, NH), 2994 (CH), 2923 (CH), 1664 (C=O), 1543, 1474 (CH), 1364, 1271, 1089 (C-O), 1049 (C-O), 1019, 855, 765. Major diastereomer: ^1^H NMR (600 MHz, DMSO-*d*_6_): δ = 1.77–1.78 (m, 3 H, 5′-CH_3_^T^), 1.80 (d, *J_CH3T,6_* = 1.0 Hz, 3 H, 3′-CH_3_^T^), 1.85–1.94 (m, 1 H, 3′-H5′a), 1.99–2.04 (m, 1 H, 3′-H2′a), 2.08–2.22 (m, 4 H, 5′-H’2a, 5′-H2′b, 3′-H2′b, 3′-H5′b), 3.18 (dd, *J* = 6.0 Hz, *J* = 8.5 Hz, 1 H, 3′-H6′), 3.50–3.54 (m, 1 H, 5′-H5′a), 3.55–3.58 (m, 1 H, 3′-H4′), 3.59–3.64 (m, 1 H, 5′-H5′b), 3.77–3.80 (m, 1 H, 5′-H4′), 4.02–4.05 (m, 1 H, 3′-H3′), 4.26–4.33 (m, 1 H, 5′-H3′), 5.08–5.11 (m, 1 H, 5′-OH), 5.32 (d, *J* = 4.4 Hz, 1 H, 3′-OH), 6.08–6.11 (m, 1 H, 3′-H1′), 6.17–6.19 (m, 1 H, 5′-H1′), 7.44 (bs, 1 H, 5′-H6), 7.75 (app. d, *J* = 1.0 Hz, 1 H, 3′-H6), 8.37 (d, *J_3′,NH_* = 7.3 Hz, 1 H, CONH), 8.96 (bs, 1 H, CONHO), 10.59 (bs, 1 H, NH^T^ or NHOH), 11.27 (bs, 1 H, NH^T^ or NHOH) ppm. ^13^C NMR (151 MHz, DMSO-*d*_6_): δ = 12.08 (3′-CH_3_^T^), 12.3 (5′-CH_3_^T^), 32.4 (3′-C5′), 36.9 (5′-C2′), 38.4 (53-C2′), 47.2 (3′-C6′), 49.3 (5′-C3′), 61.2 (5′-C5′), 73.2 (3′-C3′), 83.5 (5′-C1′), 83.6 (3′-C1′), 83.9 (3′-C4′), 84.8 (5′-C4′), 109.4 (5′-C5), 109.77 (3′-C5), 136.19 (3′-C6), 136.21 (5′-C6), 150.39 (3′-C2), 150.44 (5′-C2), 163.7 (5′-C4, 3′-C4), 165.6 (CONHOH), 168.7 (CONHC) ppm. Minor diastereomer: ^1^H NMR (600 MHz, DMSO-*d*_6_): δ = 1.77–1.78 (m, 3 H, 5′-CH_3_^T^), 1.81 (d, *J_CH3T,6_* = 0.9 Hz, 3 H, 3′-CH_3_^T^), 1.85–1.94 (m, 1 H, 3′-H5′a), 1.99–2.04 (m, 1 H, 3′-H2′a), 2.08–2.22 (m, 4 H, 5′-H’2a, 5′-H2′b, 3′-H2′b, 3′-H5′b), 3.21 (dd, *J* = 5.6 Hz, *J* = 8.6 Hz, 1 H, 3′-H6′), 3.50–3.54 (m, 1 H, 5′-H5′a), 3.55–3.58 (m, 1 H, 3′-H4′), 3.59–3.64 (m, 1 H, 5′-H5′b), 3.77–3.80 (m, 1 H, 5′-H4′), 4.02–4.05 (m, 1 H, 3′-H3′), 4.26–4.33 (m, 1 H, 5′-H3′), 5.08–5.11 (m, 1 H, 5′-OH), 5.32 (d, *J* = 4.4 Hz, 1 H, 3′-OH), 6.08–6.11 (m, 1 H, 3′-H1′), 6.17–6.19 (m, 1 H, 5′-H1′), 7.44 (bs, 1 H, 5′-H6), 7.77 (app. d, *J* = 1.0 Hz, 1 H, 3′-H6), 8.45 (d, *J_3′,NH_* = 7.3 Hz, 1 H, CONH), 8.91 (bs, 1 H, CONHO), 10.59 (bs, 1 H, NH^T^ or NHOH), 11.27 (bs, 1 H, NH^T^ or NHOH) ppm. ^13^C NMR (151 MHz, DMSO-*d*_6_): δ = 12.12 (3′-CH_3_^T^), 12.3 (5′-CH_3_^T^), 32.5 (3′-C5′), 36.7 (5′-C2′), 38.3 (53-C2′), 46.9 (3′-C6′), 49.4 (5′-C3′), 61.3 (5′-C5′), 73.2 (3′-C3′), 83.5 (5′-C1′), 83.7 (3′-C1′), 83.9 (3′-C4′), 85.1 (5′-C4′), 109.4 (5′-C5), 109.80 (3′-C5), 136.16 (5′-C6), 136.24 (3′-C6), 150.41 (3′-C2), 150.44 (5′-C2), 163.7 (5′-C4, 3′-C4), 166.0 (CONHOH), 168.3 (CONHC) ppm. HRMS (ESI^−^): *m/z* calc. 565.1900 [M − H]^−^, found: 565.1906.

### 3.3. Biological Evaluation

SNM1A (698-1040) was expressed and purified as described by Allerston et al. [8].

SNM1A (698-1040) was stored as a 1.0 µM solution in reaction buffer (20 mM HEPES-KOH, pH 7.5, 50 mM KCl, 10 mM MgCl_2_, 0.05% Triton-X, 0.1 mg/mL BSA, 5% glycerol, 0.5 mM DTT). An oligonucleotide of the sequence 5′-TTA GCA GTC AGT CAG TCA TCG-Cy3-3′ was phosphorylated using T4 PNK (New England Biolabs) [44] and used as the substrate. Modified nucleosides were added to the assay in 40% DMSO solution (10 mM or as specified). Fluorescent oligonucleotides were protected from light during incubations and gel electrophoresis.

Modified nucleosides (10 nmol or as specified) were mixed with SNM1A (698–1040) (25 fmol) in reaction buffer containing 4% DMSO (10 µL) on ice and incubated at 37 °C for 5 min. Phosphorylated oligonucleotide (0.8 pmol) was added and the reaction was incubated at 37 °C for 60 min. The reaction was stopped by the addition of stop solution (2 µL, 95% formamide, 10 mM EDTA) followed by heating to 95 °C for 3 min. The products of the reaction were analysed using gel electrophoresis; 3 µL of each reaction was used.

Oligonucleotides were separated on a 15% acrylamide 6.5 M urea gel (2.9 g urea, 2.7 mL 40% acrylamide-bisacrylamide 25:1, 1.4 mL 5× TBE (0.45 M Tris, 0.45 M boric acid, 0.01 M EDTA pH 8.0), 0.6 mL H_2_O) in 1X TBE at 150 V for 90 min alongside bromophenol blue and xylene cyanol as markers for 8 and 28 nt, respectively, and imaged using Typhoon FLA 9500.

## Figures and Tables

**Table 1:** Lowest concentrations at which inhibition was observed.

Entry	Compound	Compound Family	Substituent	Lowest Inhibitory Concentration ^1^ (µM)
1	**9**	5′-*C* Malonate	ONa, OMe	333
2	**10**	5′-*C* Malonate	ONa, ONa	333
3	**11**	5′-*C* Malonate	ONa, NHOH	33
4	**13**	5′-*N*-Malonamide	OMe	-
5	**14**	5′-*N*-Malonamide	ONa	-
6	**15**	5′-*N*-Malonamide	NHOH	333
7	**26**	3′-*N*-Malonamide	OMe	-
8	**27**	3′-*N*-Malonamide	ONa	333
9	**28**	3′-*N*-Malonamide	NHOH	333
10	**34**	Dinucleoside	OMe	1000
11	**35**	Dinucleoside	ONa	333
12	**36**	Dinucleoside	NHOH	333

^1^ As observed in concentration gradient experiments, using approximately 3-fold dilutions.

## Data Availability

The data presented in this study are available in the Supplementary Material.

## Supplementary Materials

The following are available online, Figures S1–S4: Concentration-dependence studies of compounds **9**, **10**, **15**, **27**, **28**, **34**–**36;** ^1^H and ^13^C NMR spectra.

## Sample Availability

Samples of the compounds are not available from the authors.
