# Supplementary material for: Probing the Binding Requirements of Modified Nucleosides with the DNA Nuclease SNM1A
Source: Molecules. 2021 Jan 9;26(2):320. doi: 10.3390/molecules26020320 (PMC7827217; doi:10.3390/molecules26020320)

Supporting information for

Probing the binding requirements of modified nucleosides with the DNA nuclease SNM1A

Eva-Maria Dürr [1] and Joanna F. McGouran [1,*]

[1] School of Chemistry and Trinity Biomedical Sciences Institute, Trinity College Dublin, 152-160 Pearse St., Dublin 2, Ireland.

1. Additional concentration-dependence studies 2
2. ^{1}H and ^{13}C NMR spectra 5

1. Additional concentration-dependence studies

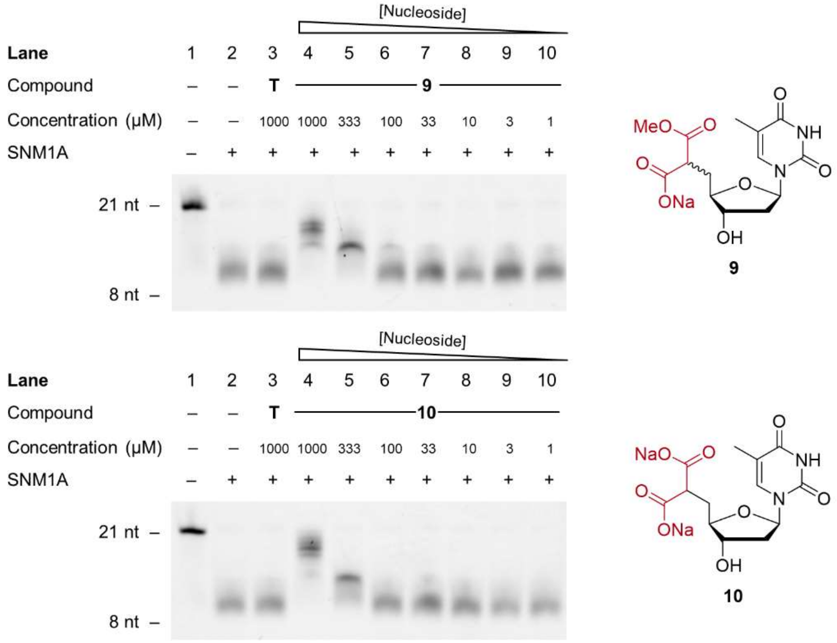

Figure S1: Concentration-dependence studies of 5′-*C*-linked malonates **9** and **10**. Digestion of the phosphorylated oligonucleotide (0.8 pmol, 80 nM) after incubation with SNM1A (25 fmol, 2.5 nM) and nucleosides **9** or **10** (1000-1 µM) or thymidine (**T**) (1 mM) for 60 min at 37 °C, analysed by denaturing PAGE. SNM1A was preincubated with the modified nucleoside for 5 min at 37 °C prior to the addition of the substrate. nt = nucleotides.

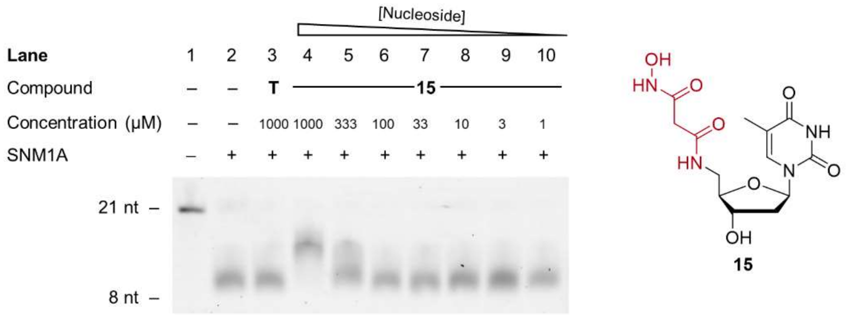

Figure S2: Concentration-dependence studies of 5′-*N*-linked malonate **15**. Digestion of the phosphorylated oligonucleotide (0.8 pmol, 80 nM) after incubation with SNM1A (25 fmol, 2.5 nM) and nucleosides **15** (1000-1 µM) or thymidine (**T**) (1 mM) for 60 min at 37 °C, analysed by denaturing PAGE. SNM1A was preincubated with the modified nucleoside for 5 min at 37 °C prior to the addition of the substrate. nt = nucleotides.

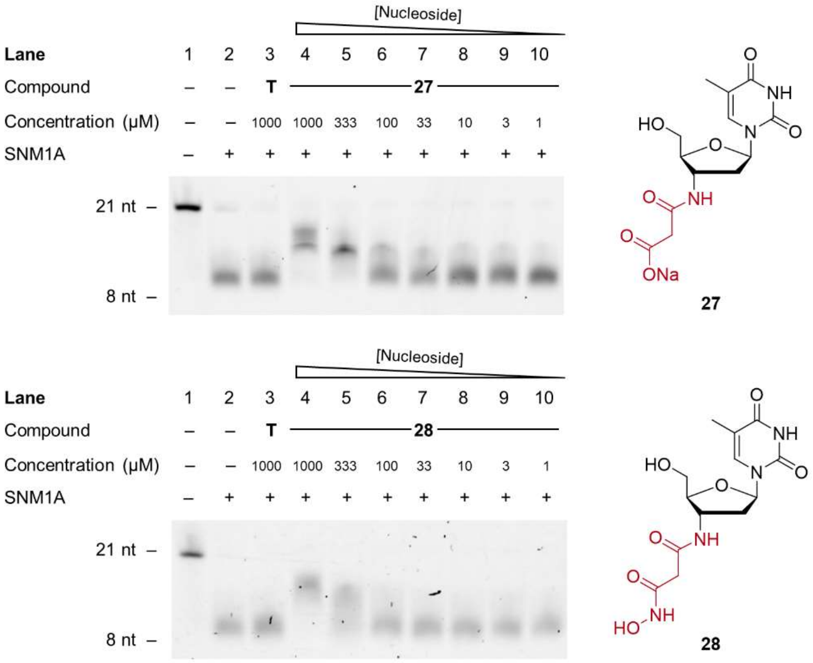

Figure S3: Concentration-dependence studies of 3′-*N*-linked malonates **27** and **28**. Digestion of the phosphorylated oligonucleotide (0.8 pmol, 80 nM) after incubation with SNM1A (25 fmol, 2.5 nM) and nucleosides **27** or **28** (1000-1 μM) or thymidine (**T**) (1 mM) for 60 min at 37 °C, analysed by denaturing PAGE. SNM1A was preincubated with the modified nucleoside for 5 min at 37 °C prior to the addition of the substrate. nt = nucleotides.

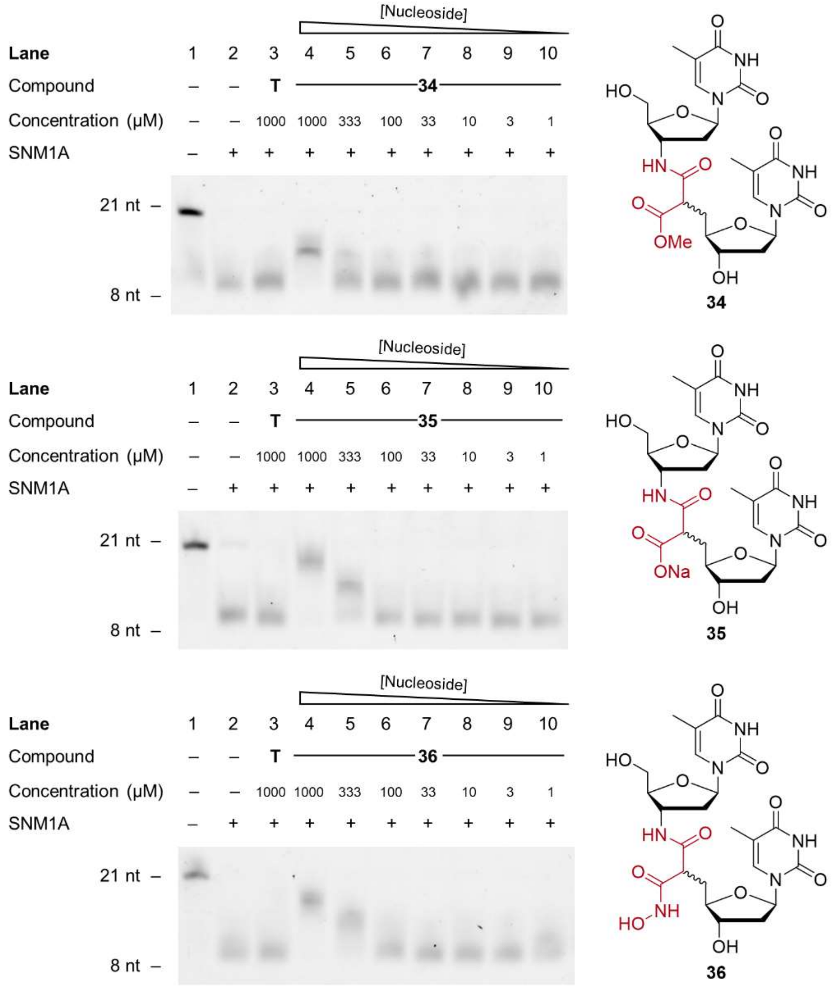

Figure S4: Concentration-dependence of dinucleosides **34-36**. Digestion of the phosphorylated oligonucleotide (0.8 pmol, 80 nM) after incubation with SNM1A (25 fmol, 2.5 nM) and nucleosides **34**-**36** (1000-1 μM) or thymidine (**T**) (1 mM) for 60 min at 37 °C, analysed by denaturing PAGE. SNM1A was preincubated with the modified nucleoside for 5 min at 37 °C prior to the addition of the substrate. nt = nucleotides.

2. ^{1}H and ^{13}C NMR spectra

3′-*O*-(*tert*-Butyldimethylsilyl)-5′-deoxy-5′-iodothymidine (4)

^{1}H NMR (400 MHz, $CDCl_3$)

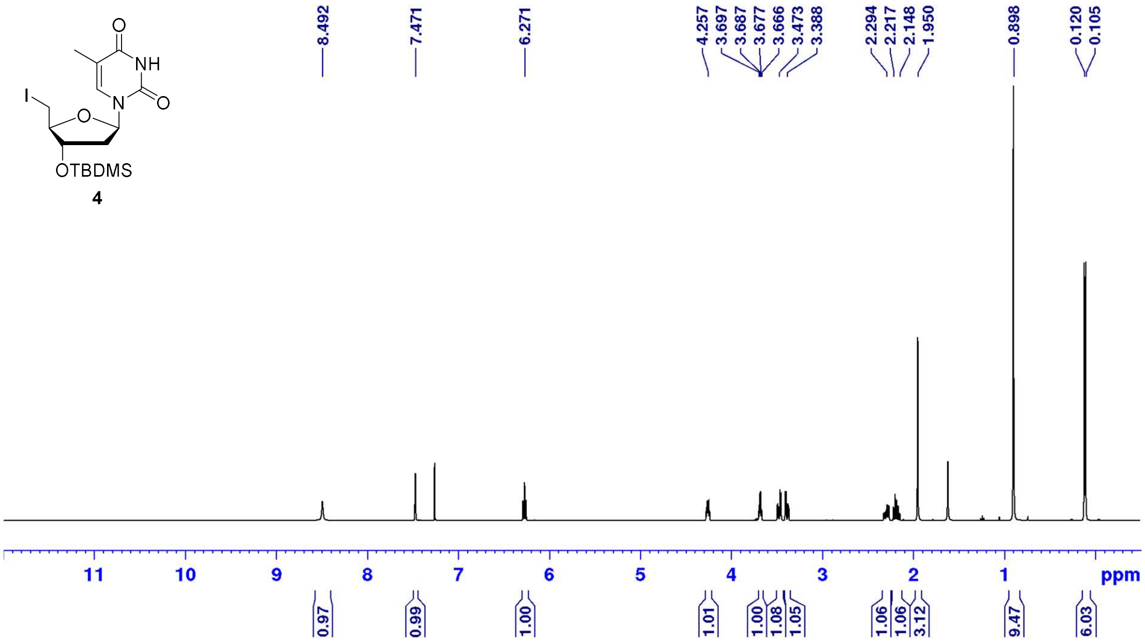

^{13}C NMR (100 MHz, $CDCl_3$)

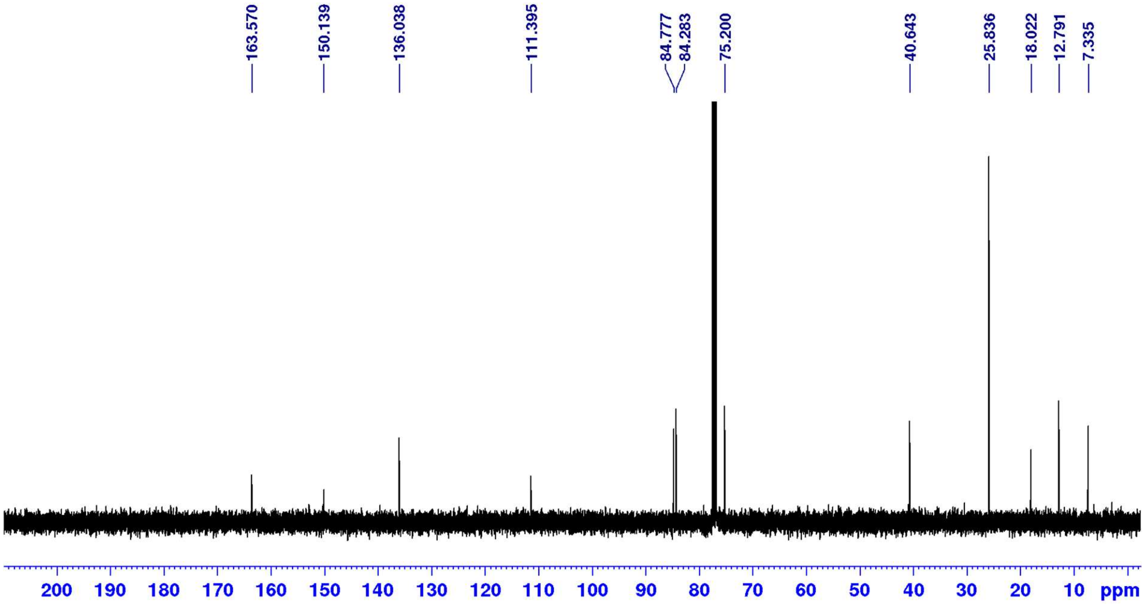

_N_-Benzyloxymethyl-3′-_O_-(_tert_-butyldimethylsilyl)-5′-deoxy-5′-iodothymidine (5)

^{1}H NMR (400 MHz, $CDCl_3$)

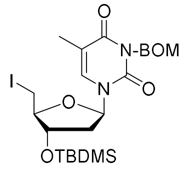

5

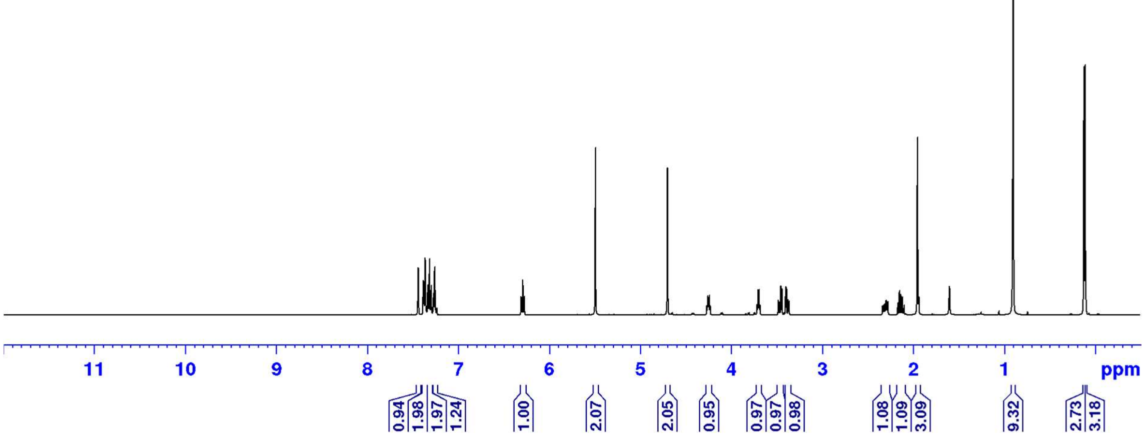

^{13}C NMR (100 MHz, $CDCl_3$)

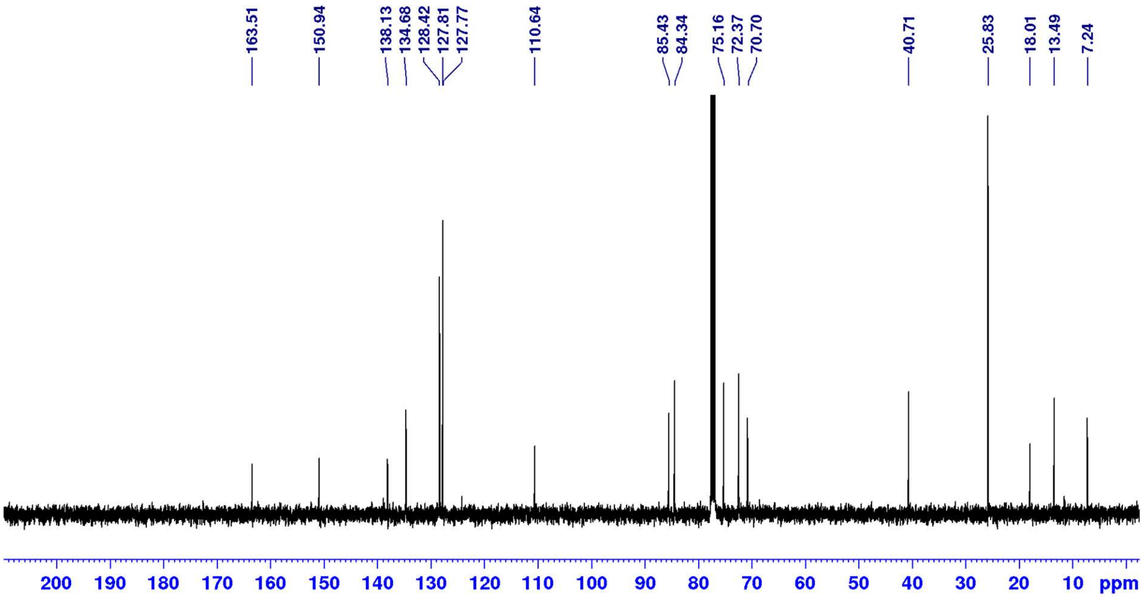

_C_-(_N_[3]-Benzyloxymethyl-3′-_O_-(_tert_-butyldimethylsilyl)-5′-deoxythymidin-5′-yl) benzyl methyl malonate (6):

^{1}H NMR (600 MHz, $CDCl_3$)

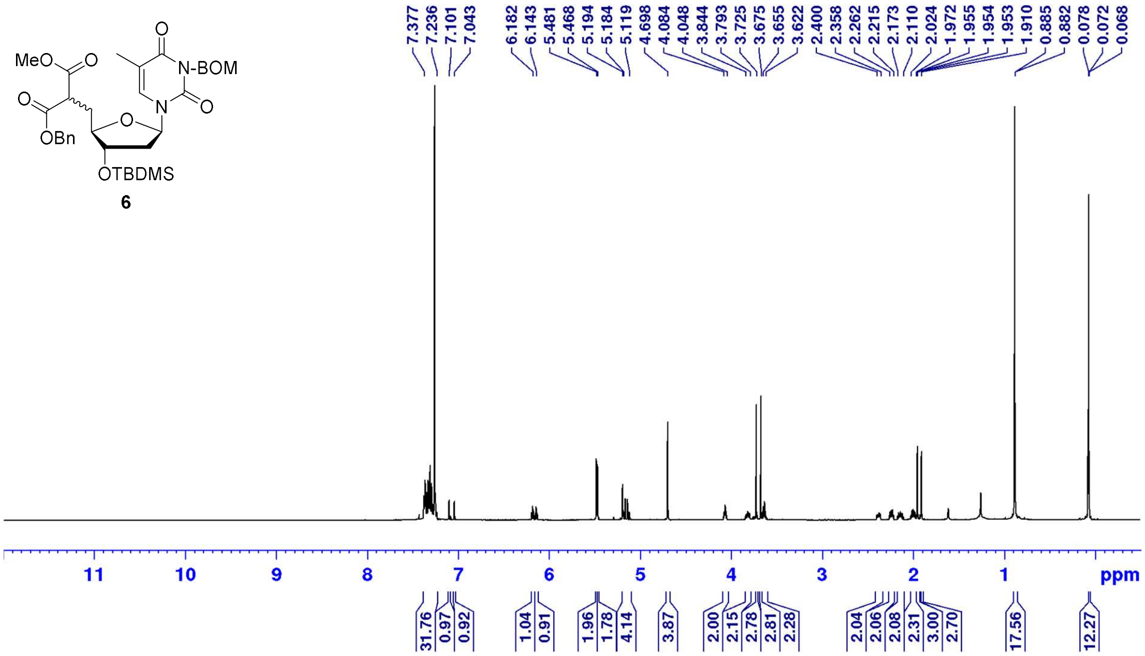

^{13}C NMR (151 MHz, $CDCl_3$)

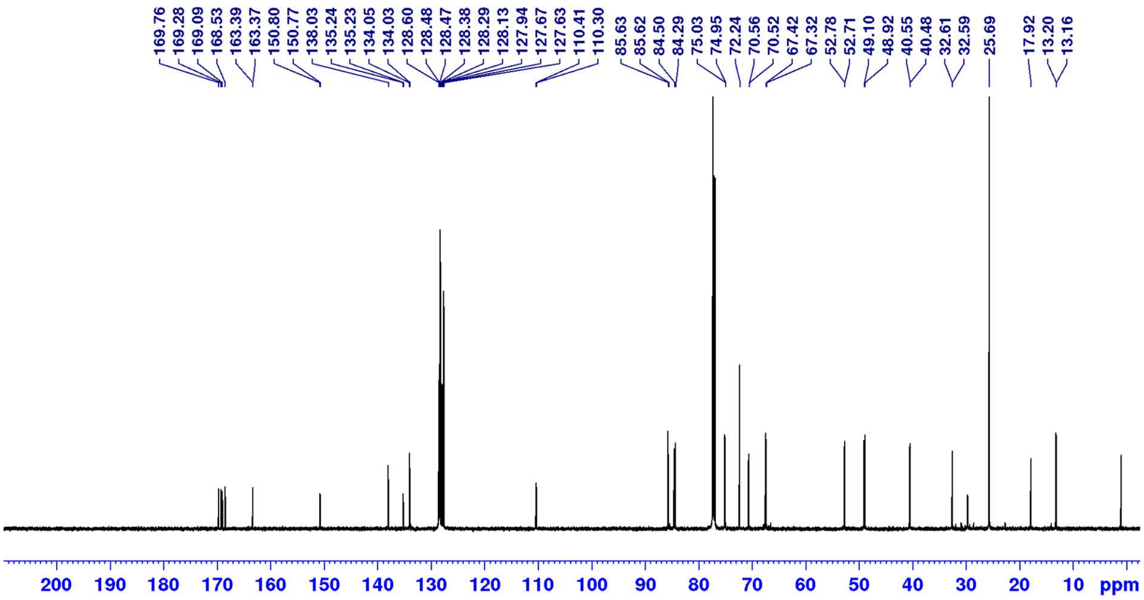

***C*-(*N*³-Benzyloxymethyl-5′-deoxythymidin-5′-yl) benzyl methyl malonate (7):**

^{1}H NMR (400 MHz, acetone-d$_6$)

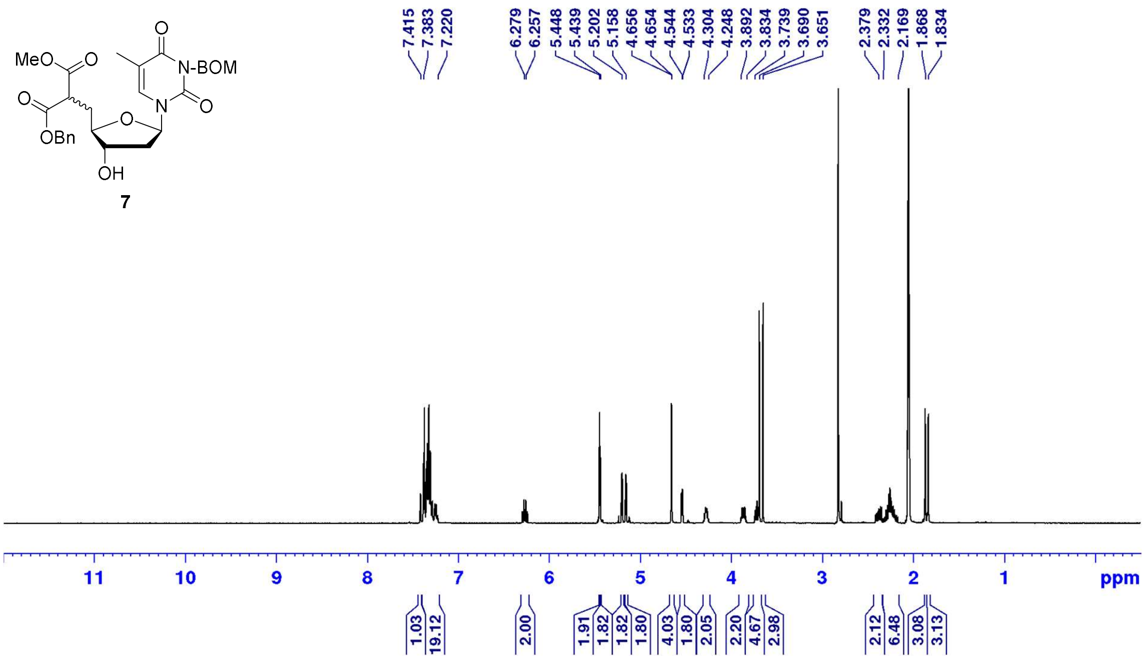

^{13}C NMR (100 MHz, acetone-d$_6$)

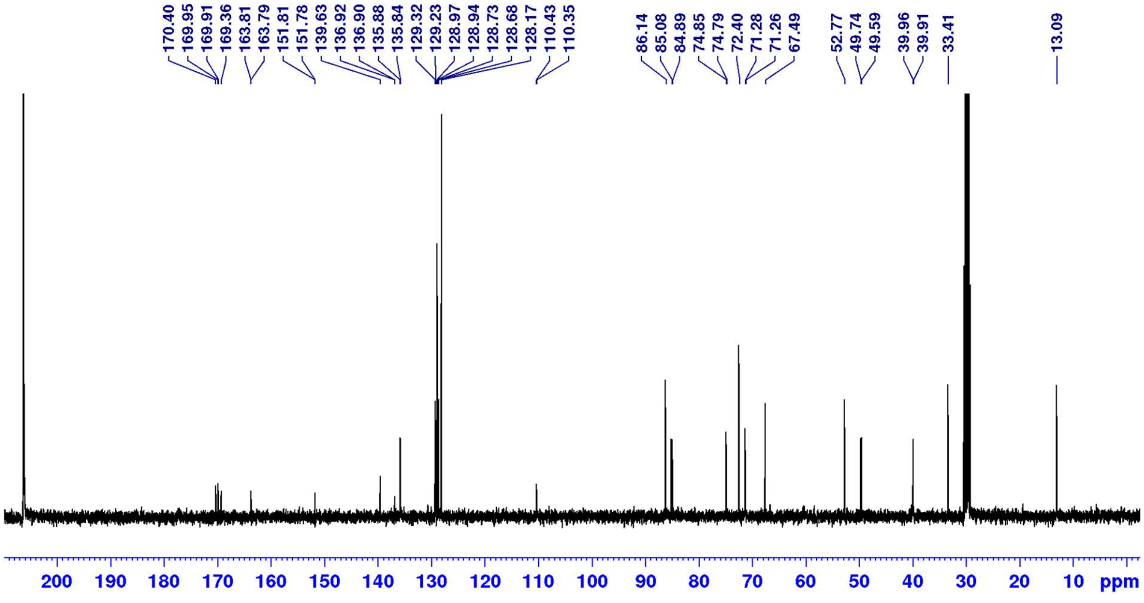

_C_-(5′-Deoxythymidin-5′-yl) malonic acid monomethyl ester (8):

^{1}H NMR (600 MHz, DMSO-d$_6$)

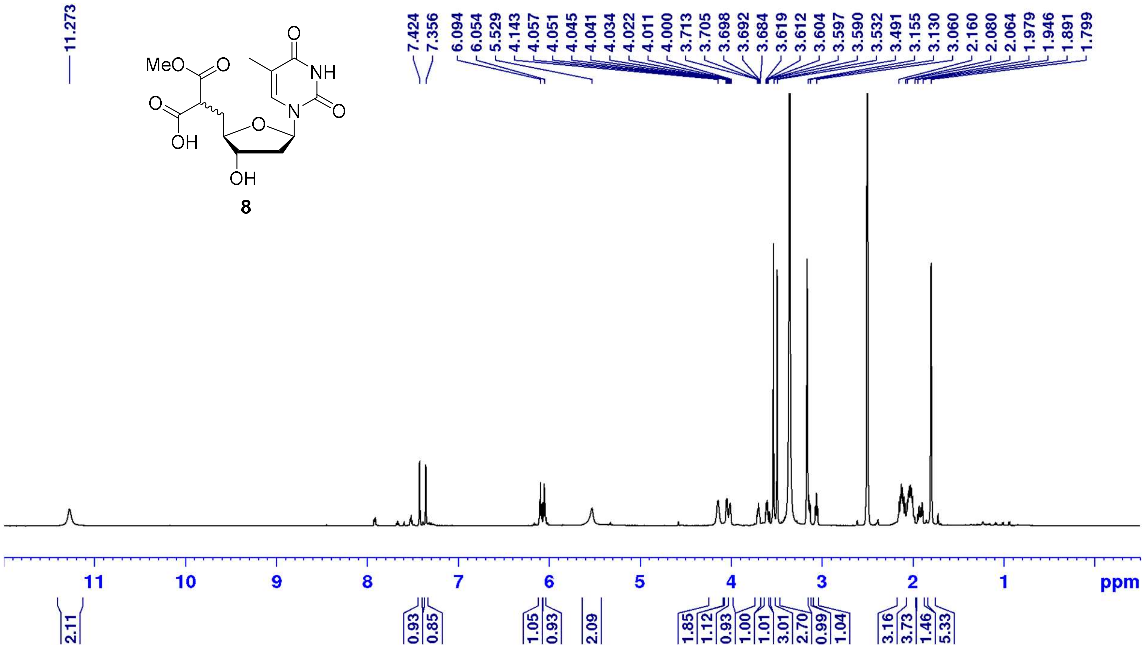

^{13}C NMR (150 MHz, DMSO-d$_6$)

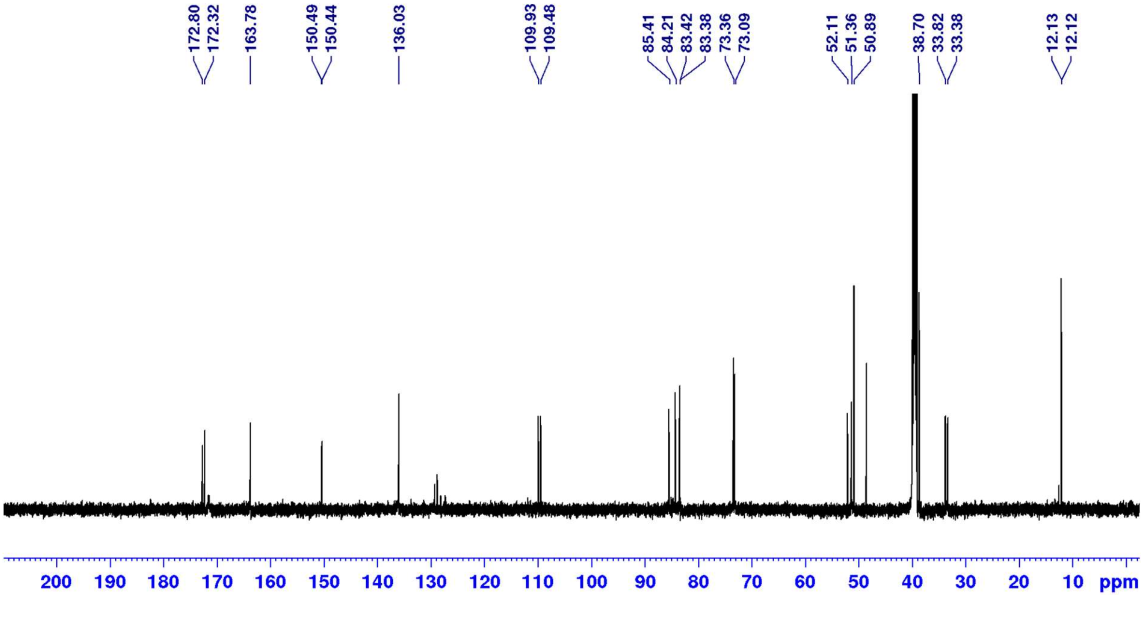

C-(5′-Deoxythymidin-5′-yl) monomethyl sodium malonate (9):

^{1}H NMR (600 MHz, DMSO-d$_6$)

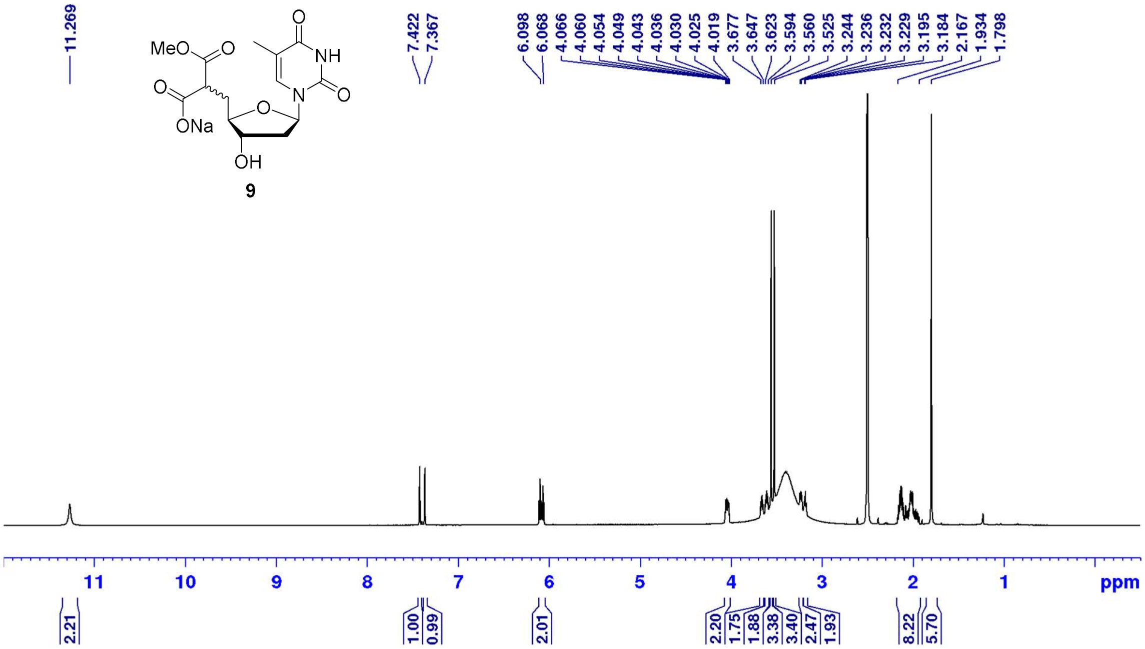

^{13}C NMR (150 MHz, DMSO-d$_6$)

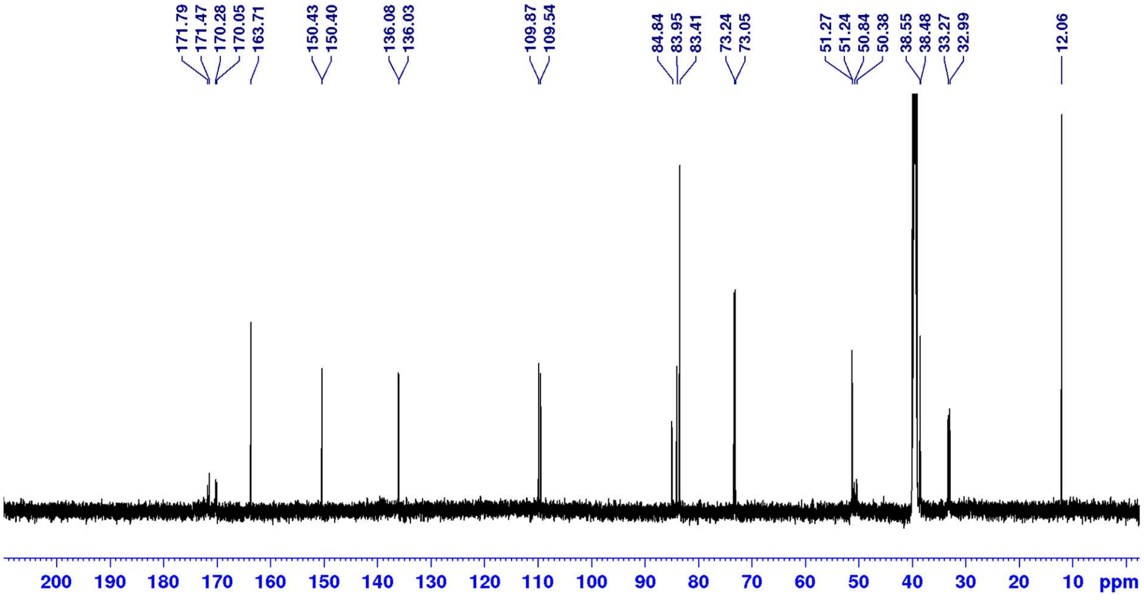

***C*-(5′-Deoxythymidin-5′-yl) disodium malonate (10):**

¹H NMR (600 MHz, D_2O)

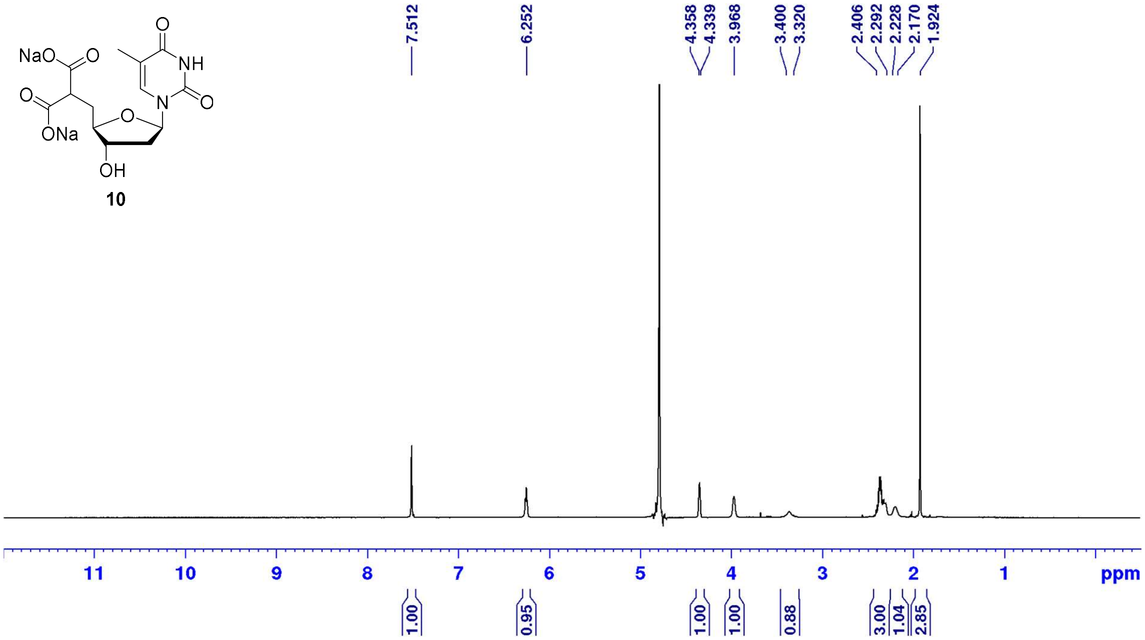

¹³C NMR (150 MHz, D_2O)

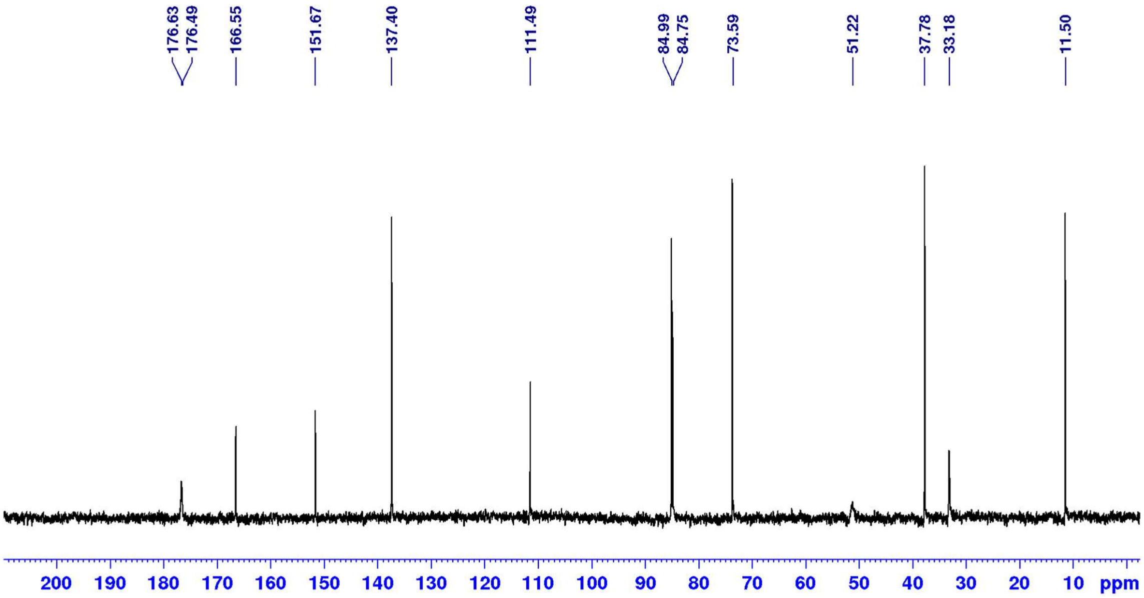

C-(5′-deoxythymidin-5′-yl) sodium N-hydroxymalonamide (11):

^{1}H NMR (600 MHz, D_2O)

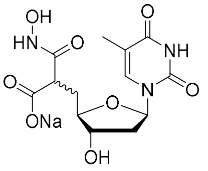

11

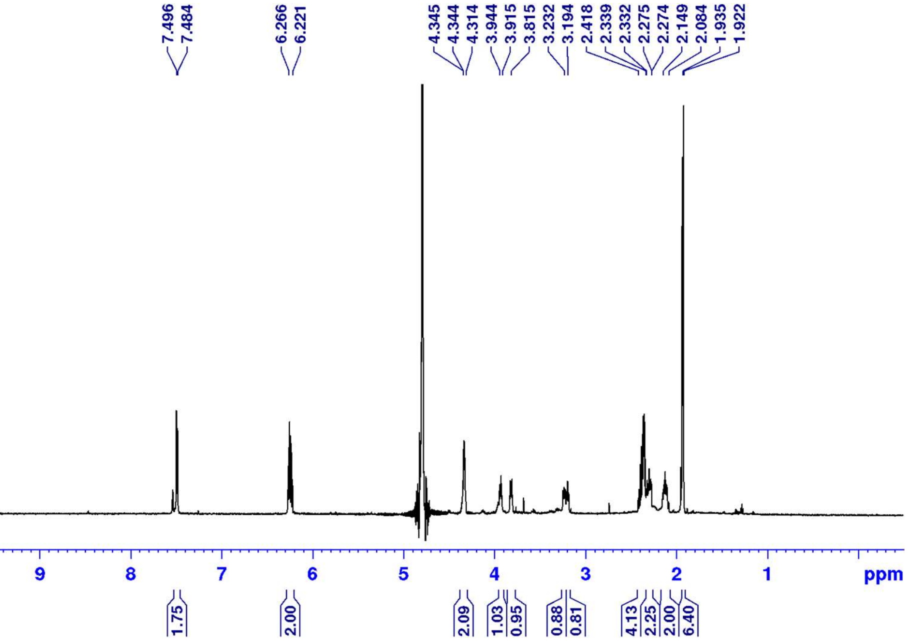

^{13}C NMR (150 MHz, D_2O)

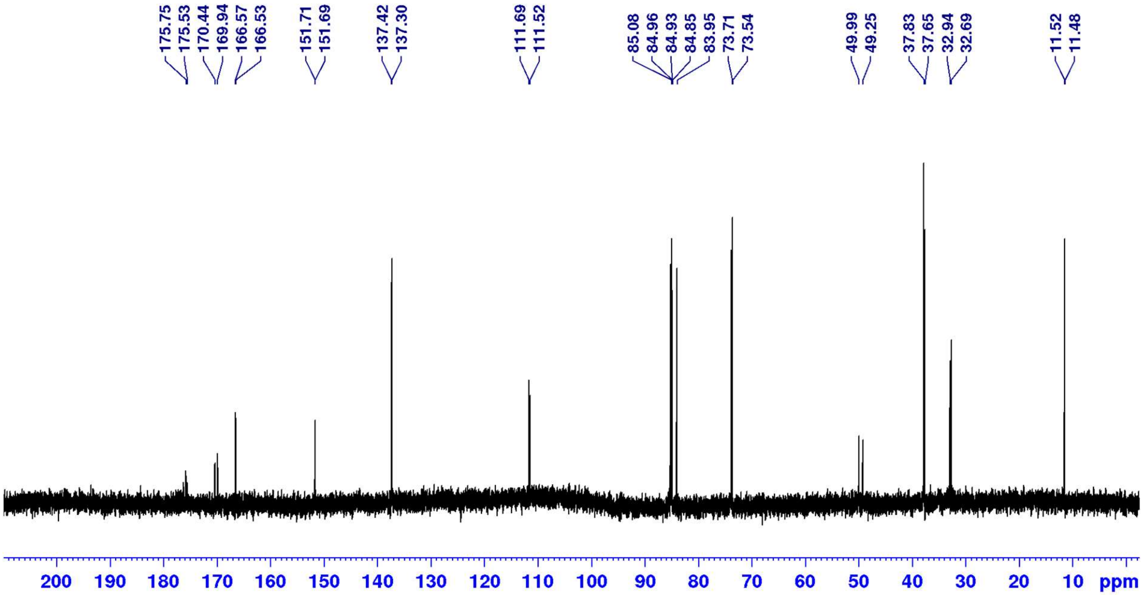

N-(5′-Deoxythymidin-5′-yl) amido methyl malonate (13):

^{1}H NMR (600 MHz, DMSO-d_6)

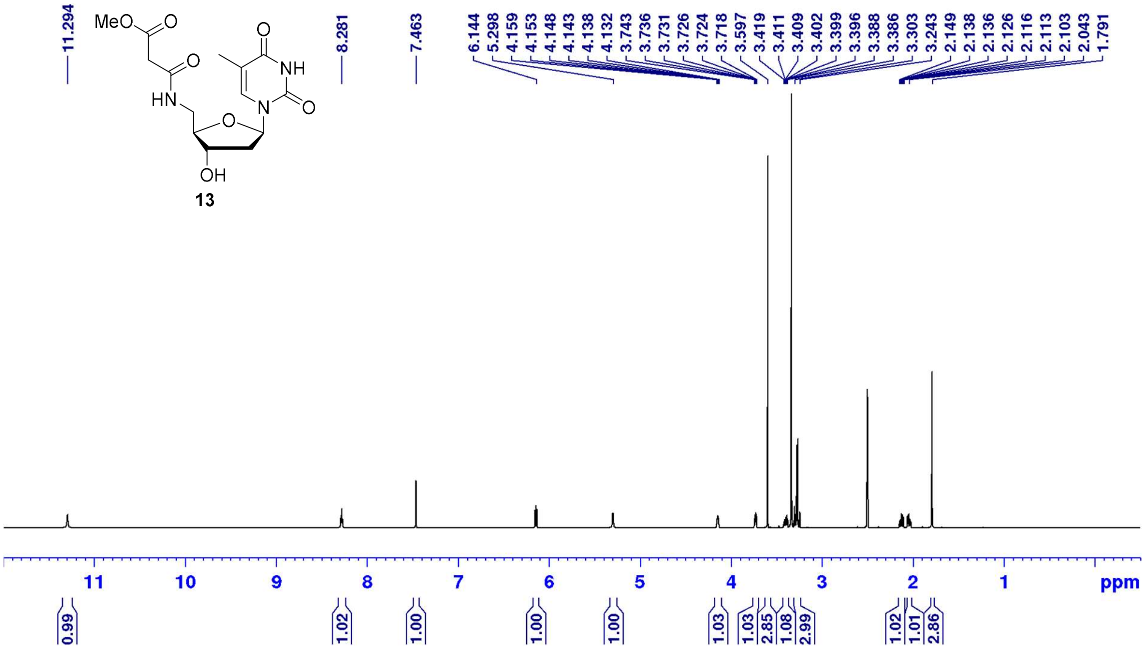

^{13}C NMR (150 MHz, DMSO-d_6)

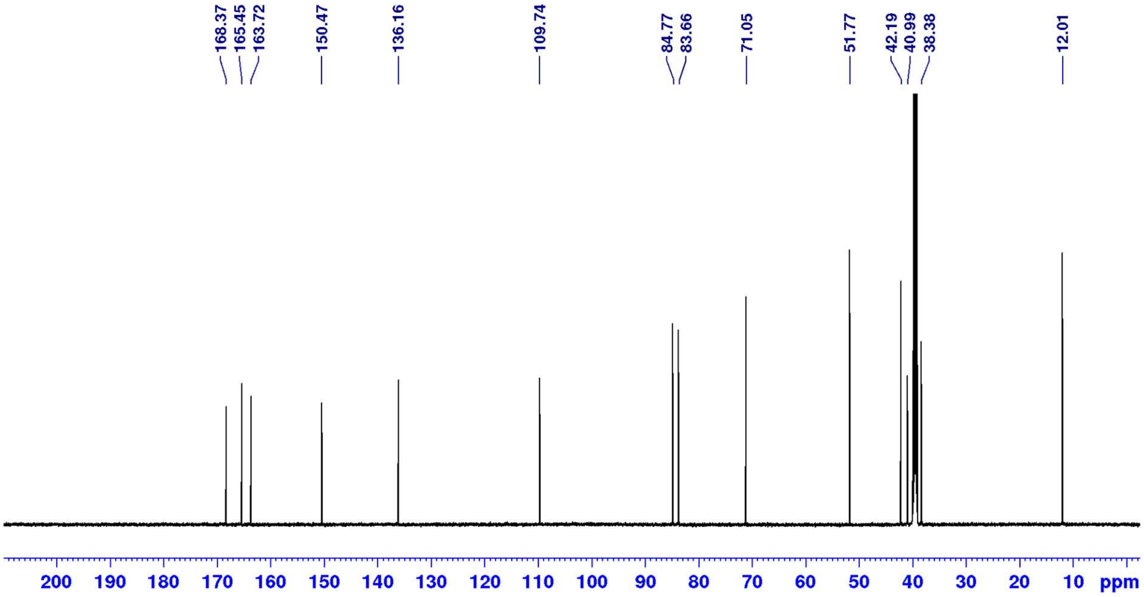

***N*-(5′-Deoxythymidin-5′-yl) amido sodium malonate (14):**

^{1}H NMR (400 MHz, D_2O)

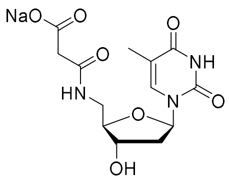

14

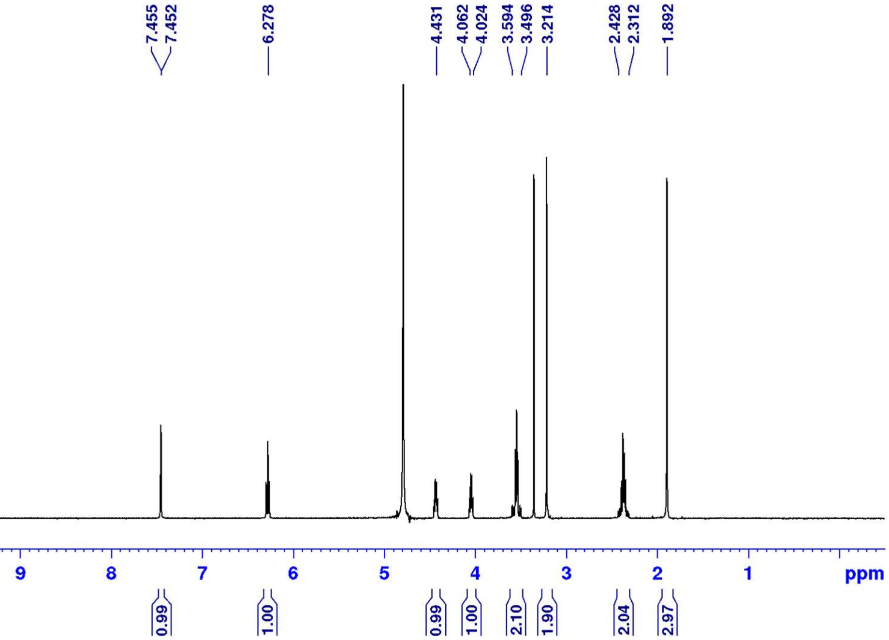

^{13}C NMR (100 MHz, D_2O)

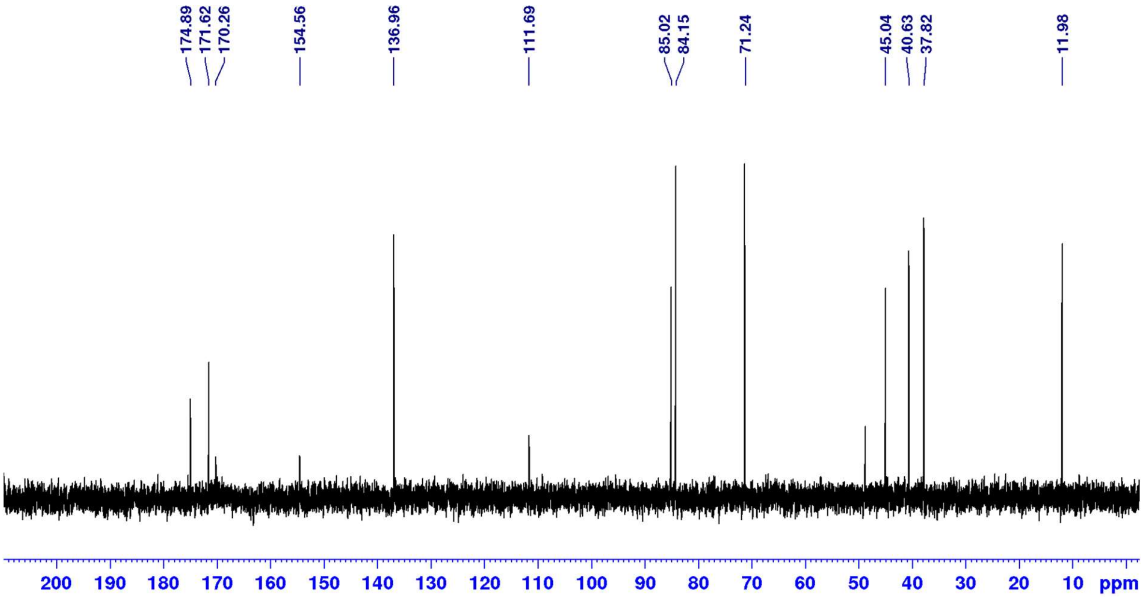

N-(5′-Deoxythymidin-5′-yl) amido N-hydroxymalonamide (15):

^{1}H NMR (600 MHz, DMSO-d6)

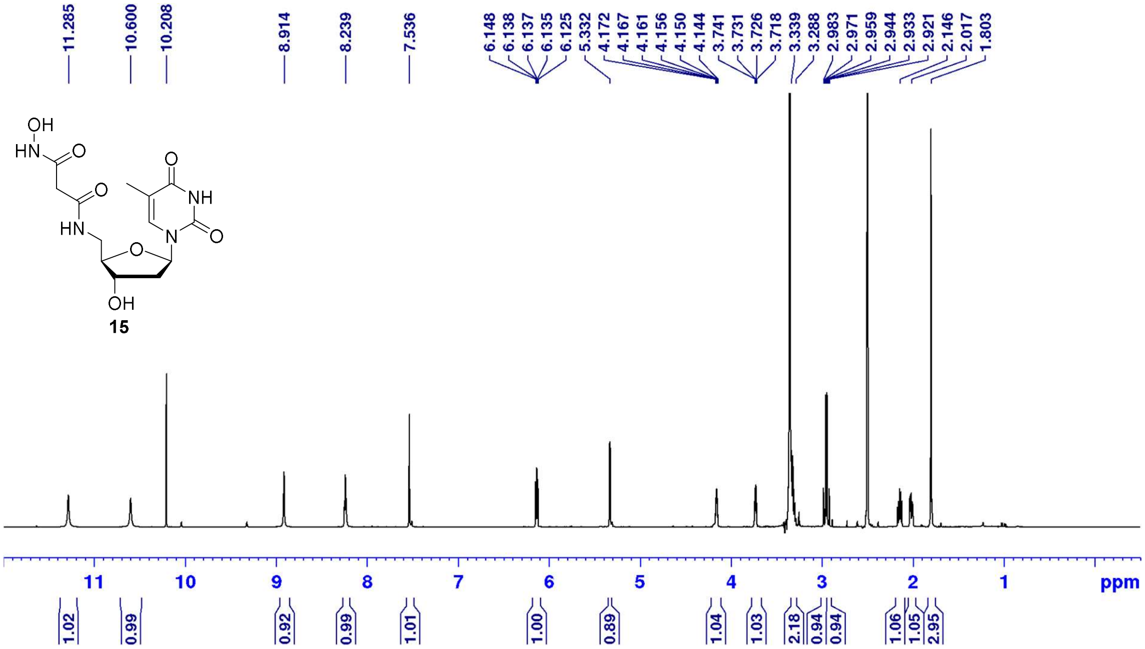

^{13}C NMR (150 MHz, DMSO-d6)

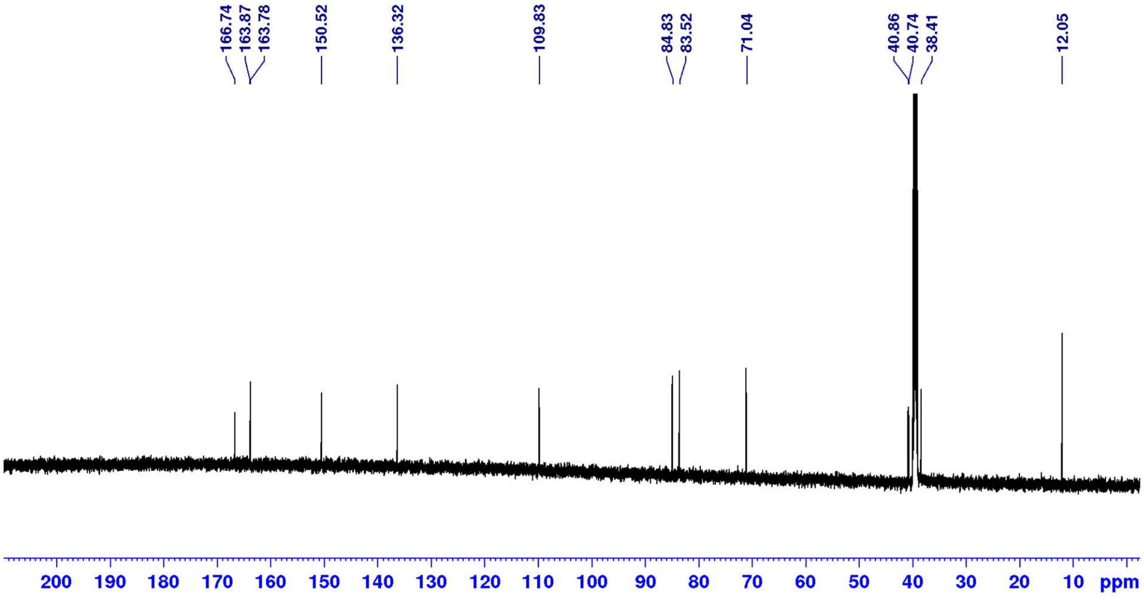

1-[5′-*O*-(4,4′-Dimethoxytrityl)-2′-deoxy-β-D-furanosyl]thymine (17):

^{1}H NMR (400 MHz, DMSO-d$_6$)

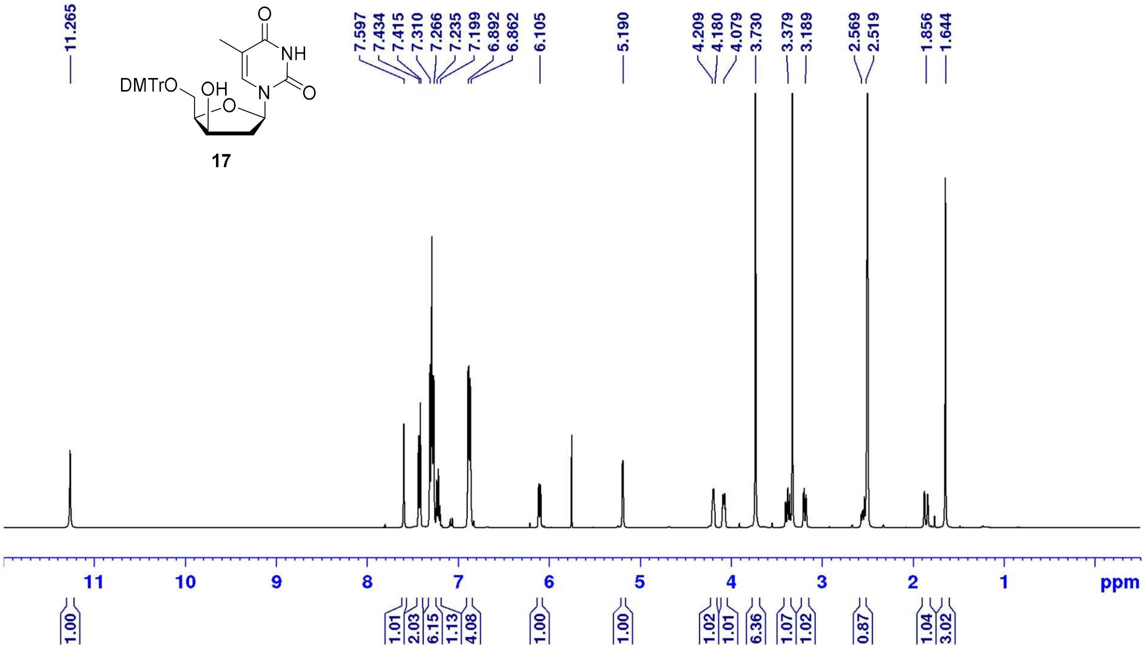

^{13}C NMR (100 MHz, DMSO-d$_6$)

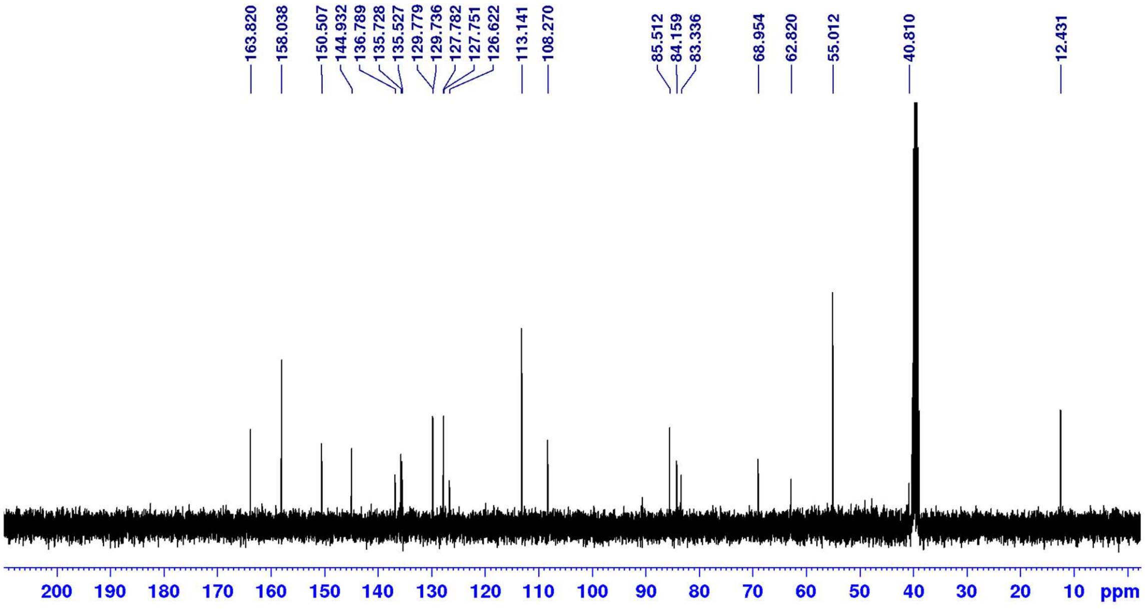

1-[5′-*O*-(4,4′-dimethoxytrityl)-3′-*O*-mesyl-2′-deoxy-β-D-furanosyl]thymine (18):

^{1}H NMR (400 MHz, acetone-d$_6$)

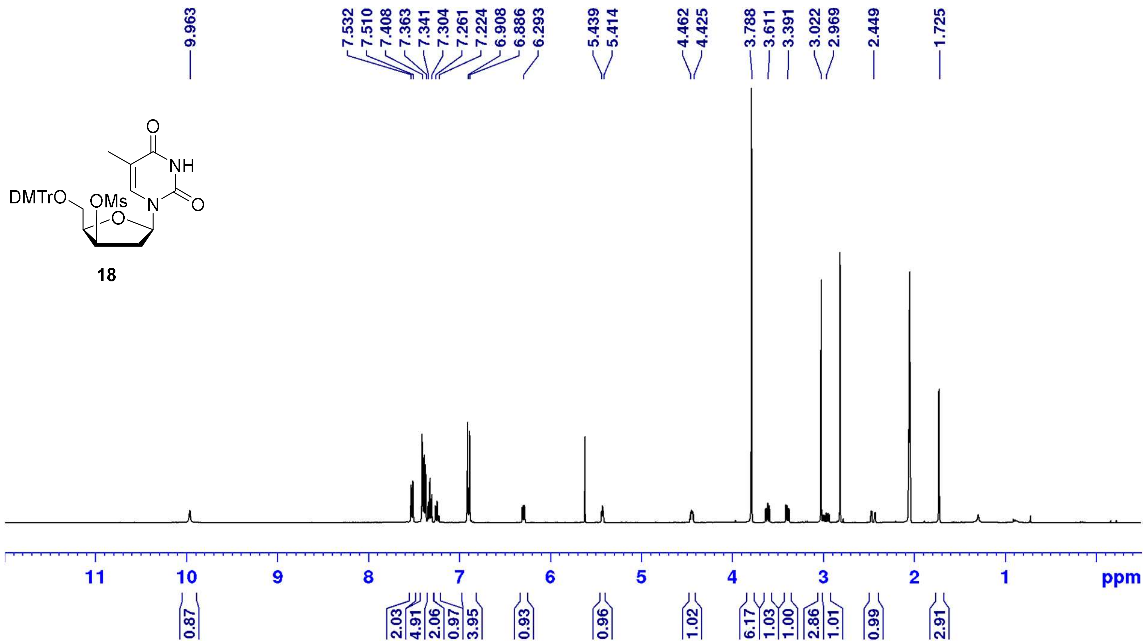

^{13}C NMR (100 MHz, acetone-d$_6$)

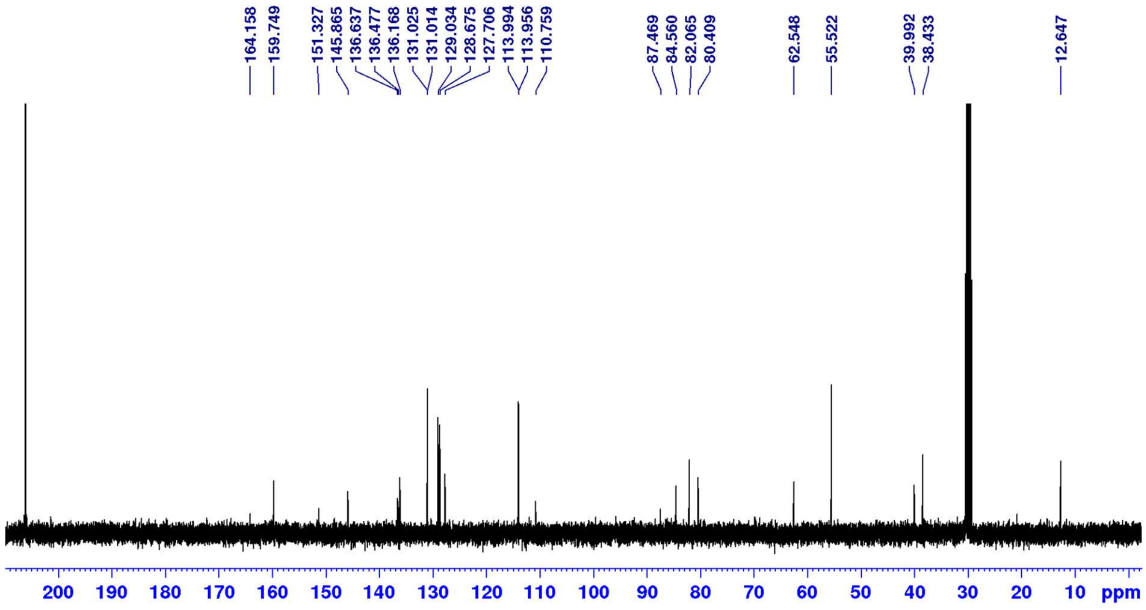

3′-Deoxy-2′,3′-didehydro-5′-*O*-(4,4'-dimethoxytrityl)-*N*³-methylthymidine (20):

^{1}H NMR (600 MHz, acetone-d$_6$)

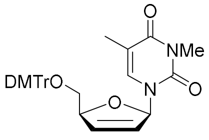

20

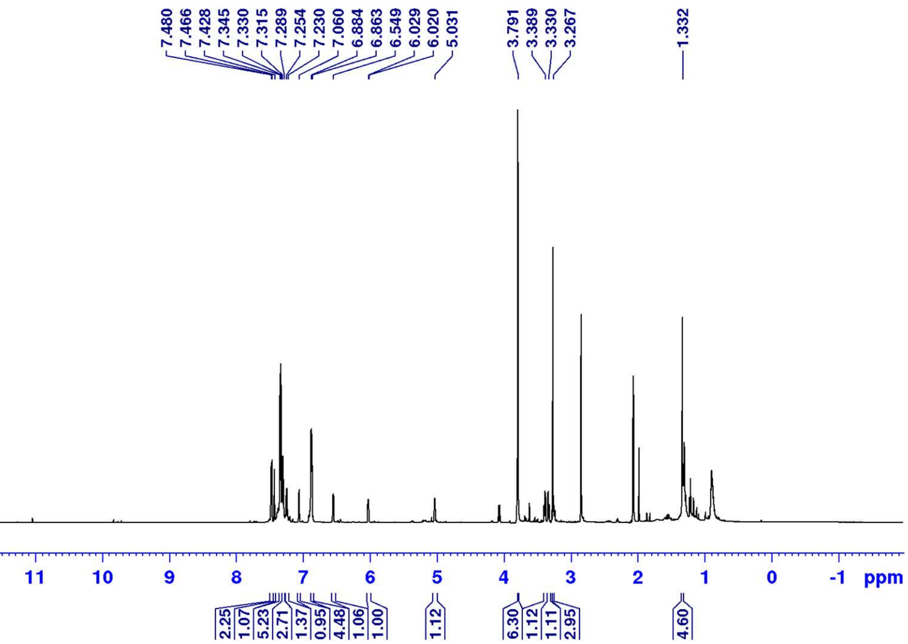

^{13}C NMR (150 MHz, acetone-d$_6$):

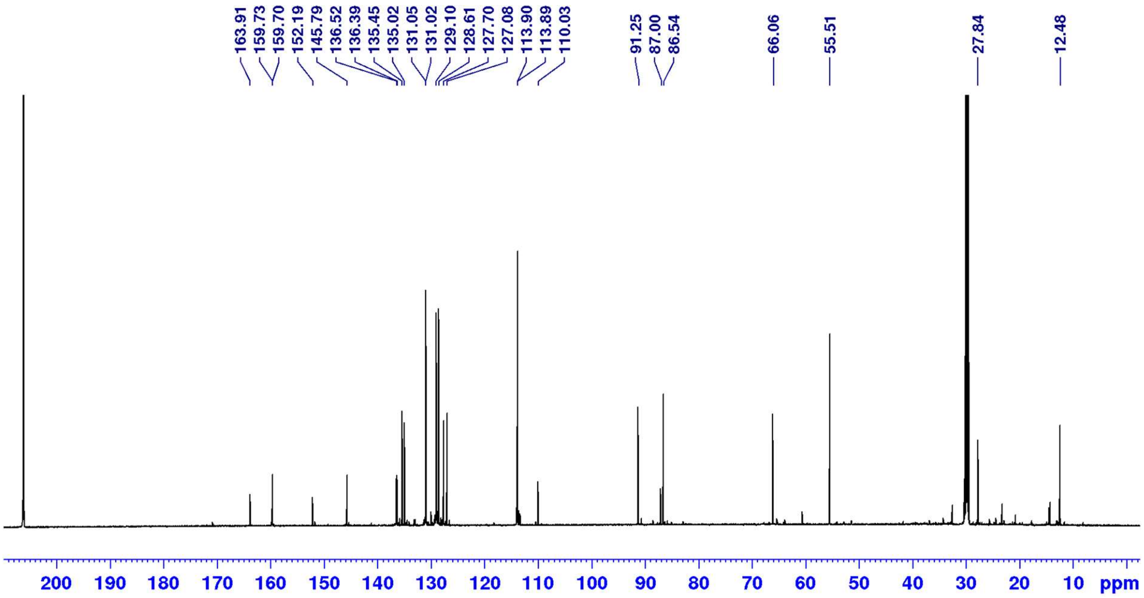

5′-*O*-(4,4'-Dimethoxytrityl)-*N*³-methyl thymidine (23):

^{1}H NMR (400 MHz, acetone-d$_6$)

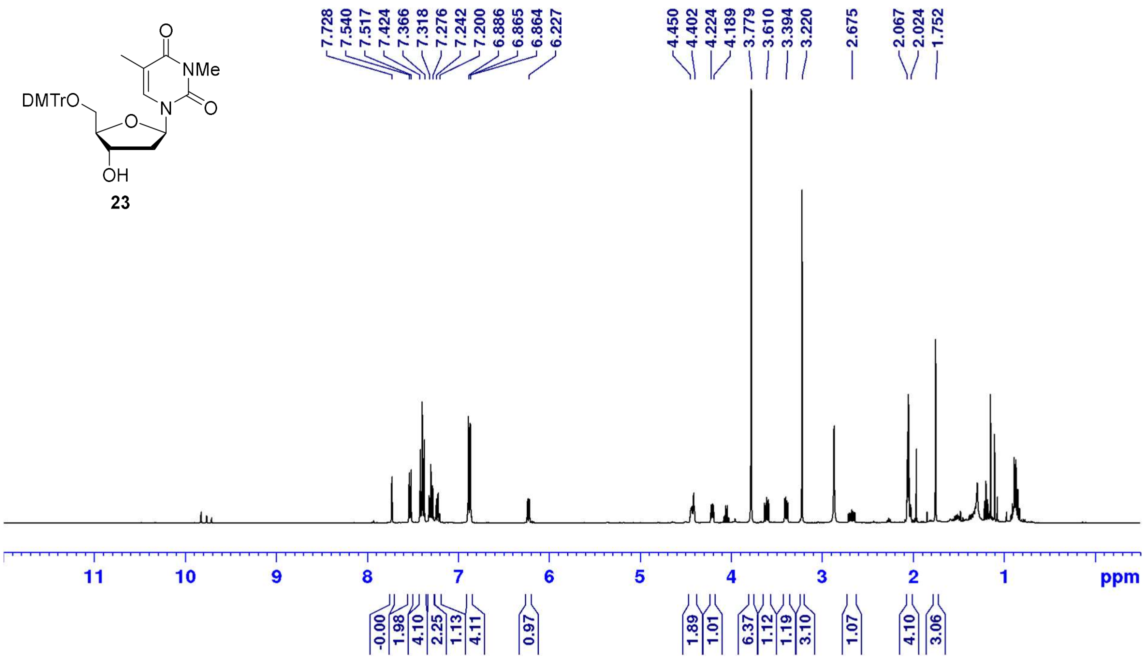

^{13}C NMR (100 MHz, acetone-d$_6$)

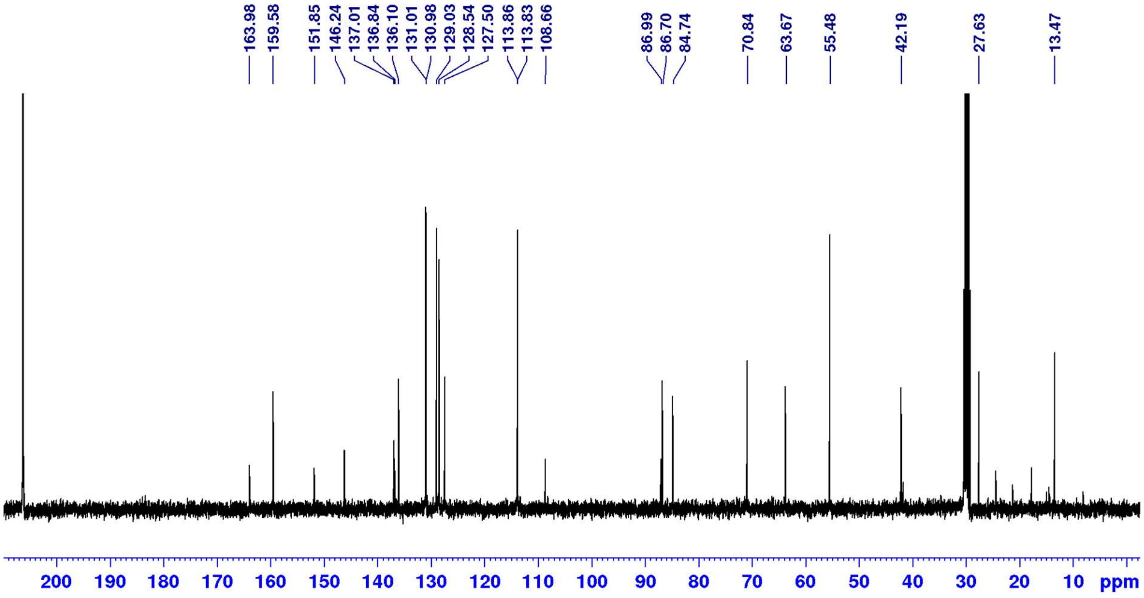

3′-Amino-3′-deoxythymidine (25):

^{1}H NMR (400 MHz, DMSO-d$_6$)

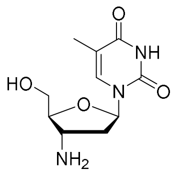

25

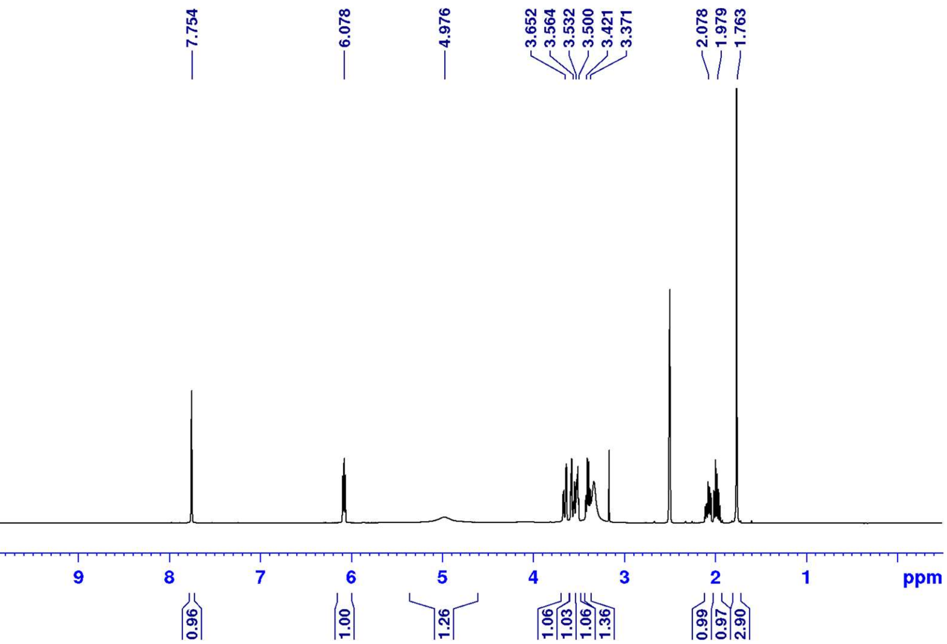

^{13}C NMR (100 MHz, DMSO-d$_6$)

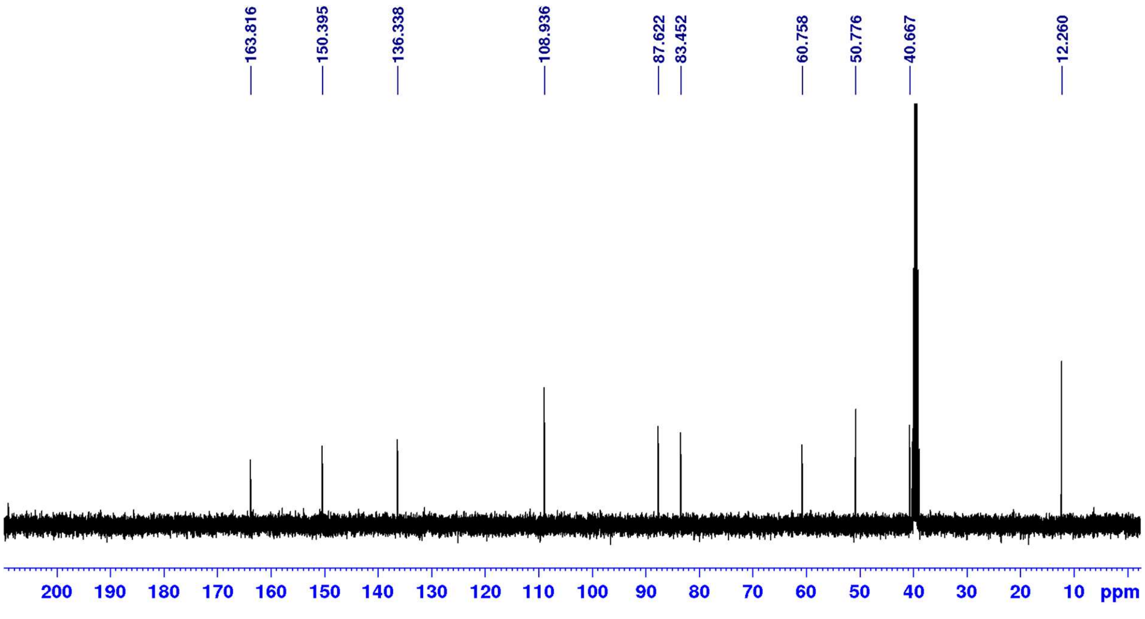

N-(3′-Deoxythymidin-3′-yl) amido methyl malonamide (26):

^{1}H NMR (400 MHz, DMSO-d$_6$)

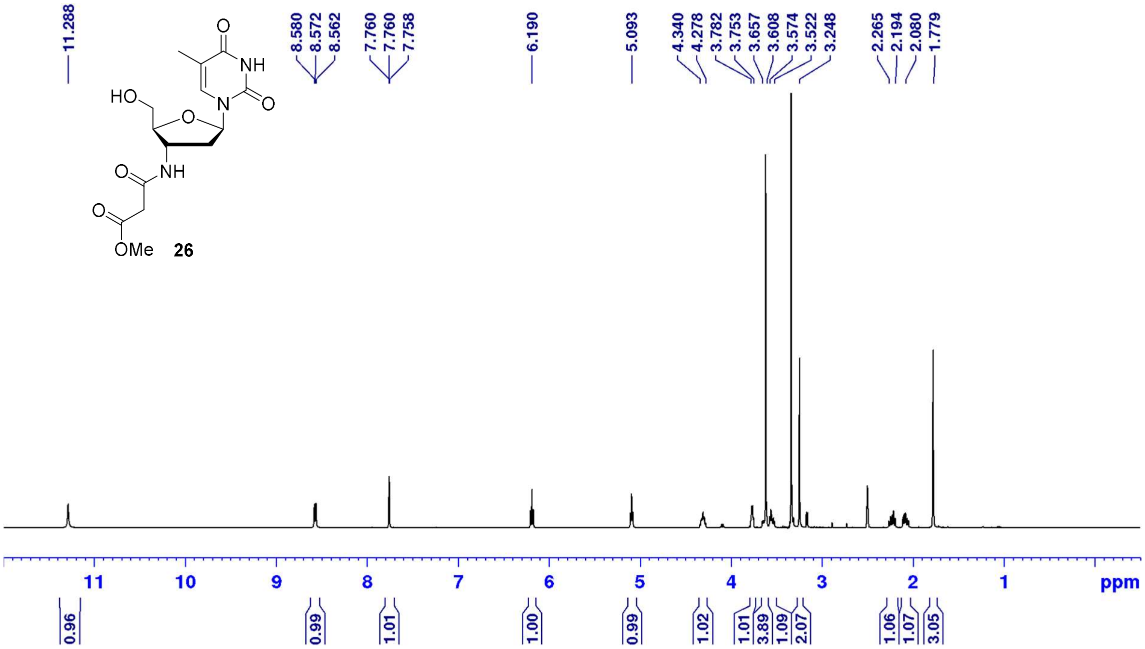

^{13}C NMR (100 MHz, DMSO-d$_6$)

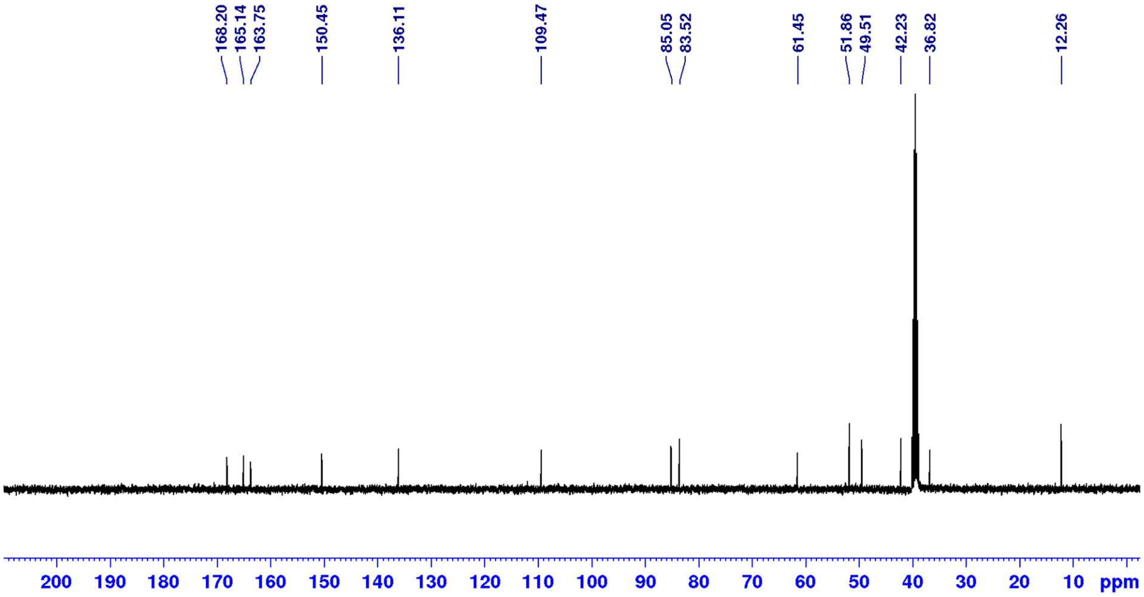

N-(3′-Deoxythymidin-3′-yl) amido sodium malonamide (27):

^{1}H NMR (600 MHz, DMSO-d$_6$)

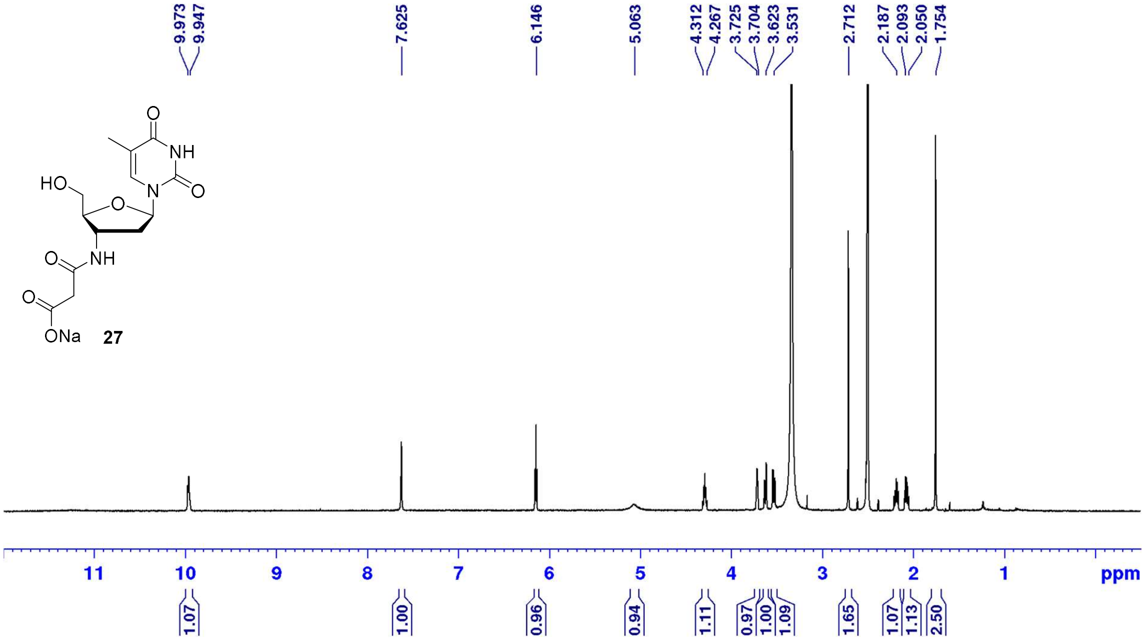

^{13}C NMR (150 MHz, DMSO-d$_6$)

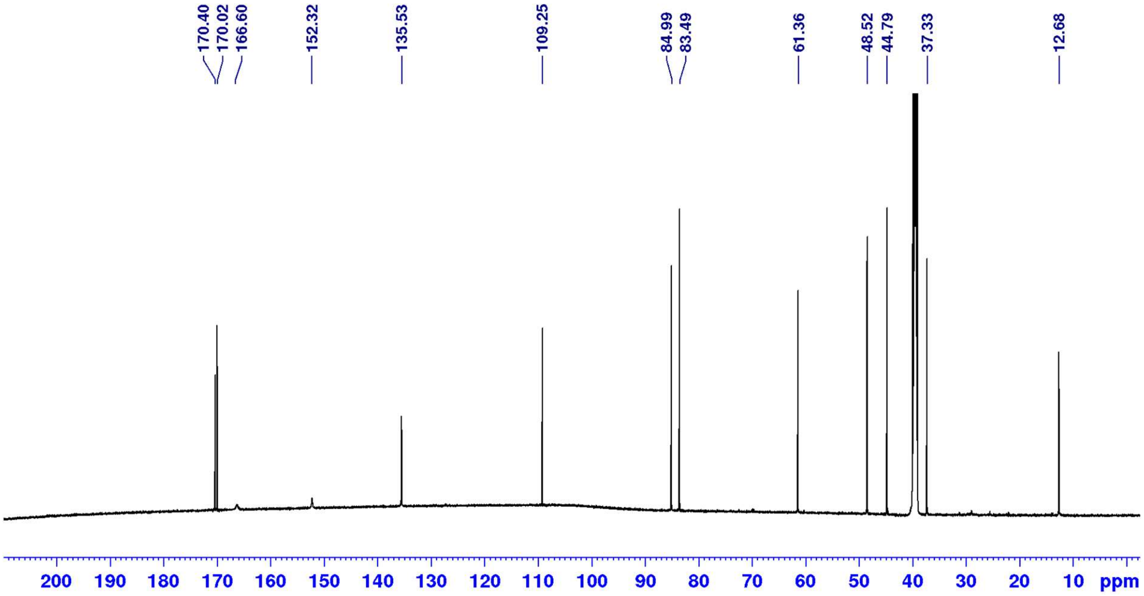

***N*-(3′-Deoxythymidin-3′-yl) amido *N*-hydroxymalonamide (28):**

^{1}H NMR (600 MHz, DMSO-d$_6$)

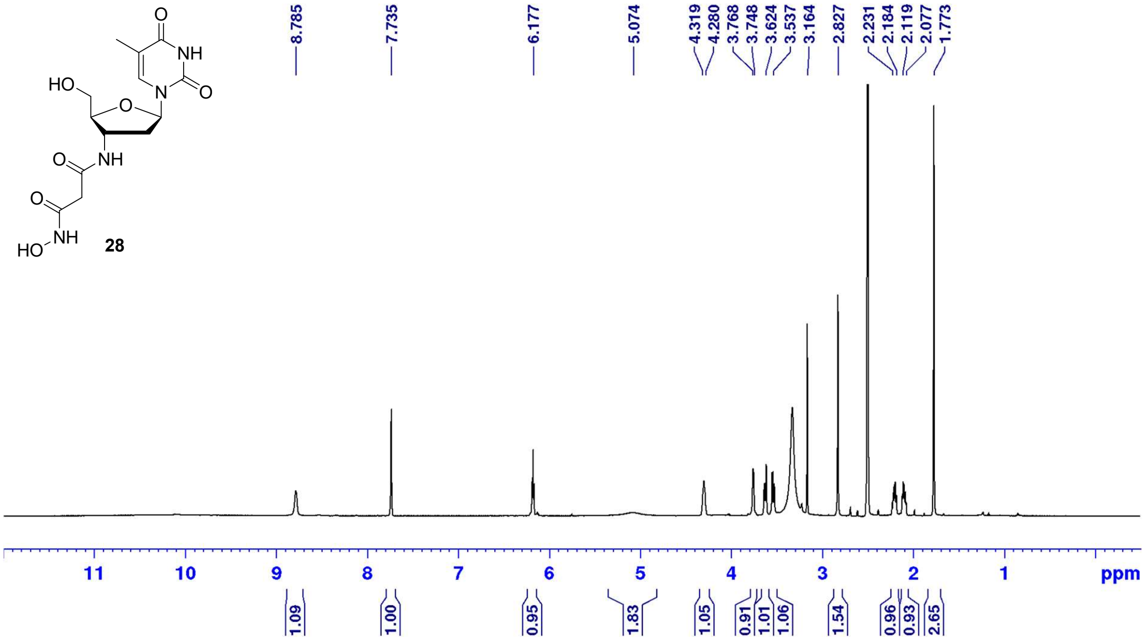

^{13}C NMR (150 MHz, DMSO-d$_6$)

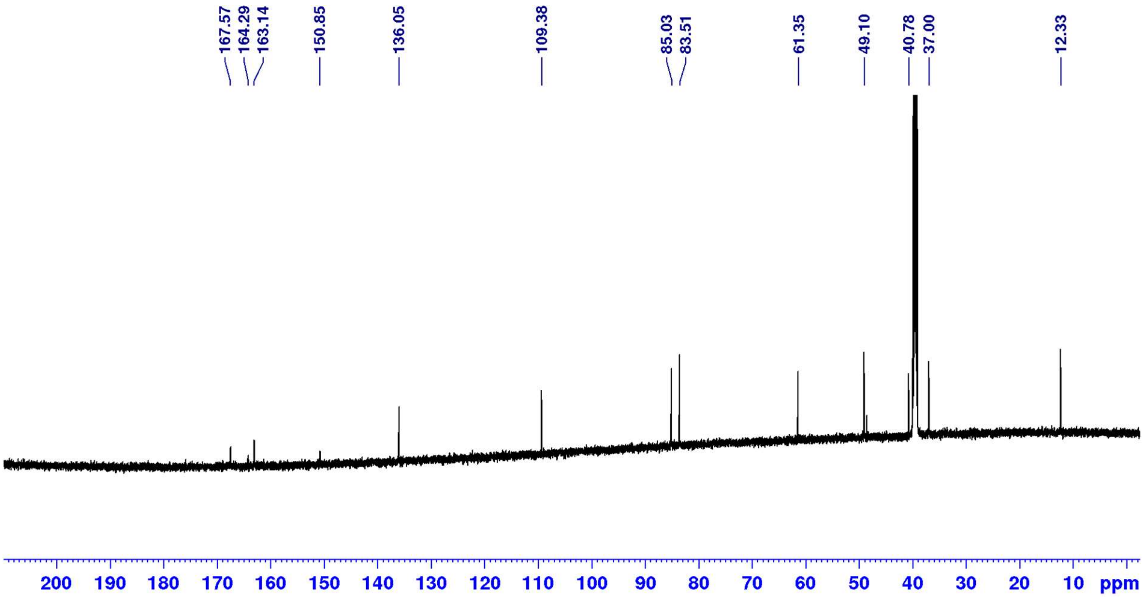

C-(3′-O-(tert-Butyldimethylsilyl-5′-deoxy-N3-hydroxymethyl)thymidin-5′-yl) malonic acid monomethyl ester (29):

^{1}H NMR (600 MHz, acetone-d$_6$)

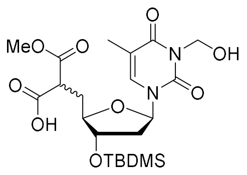

29

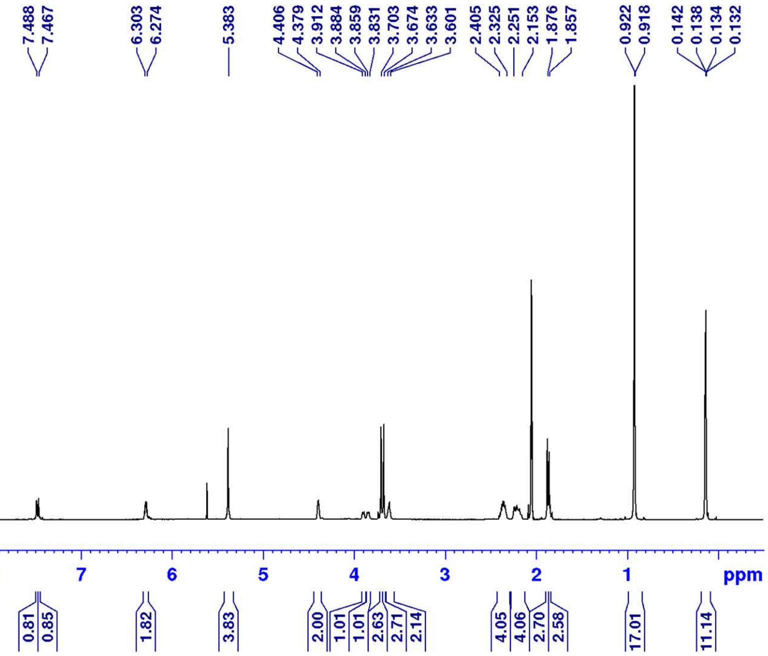

^{13}C NMR (151 MHz, acetone-d$_6$)

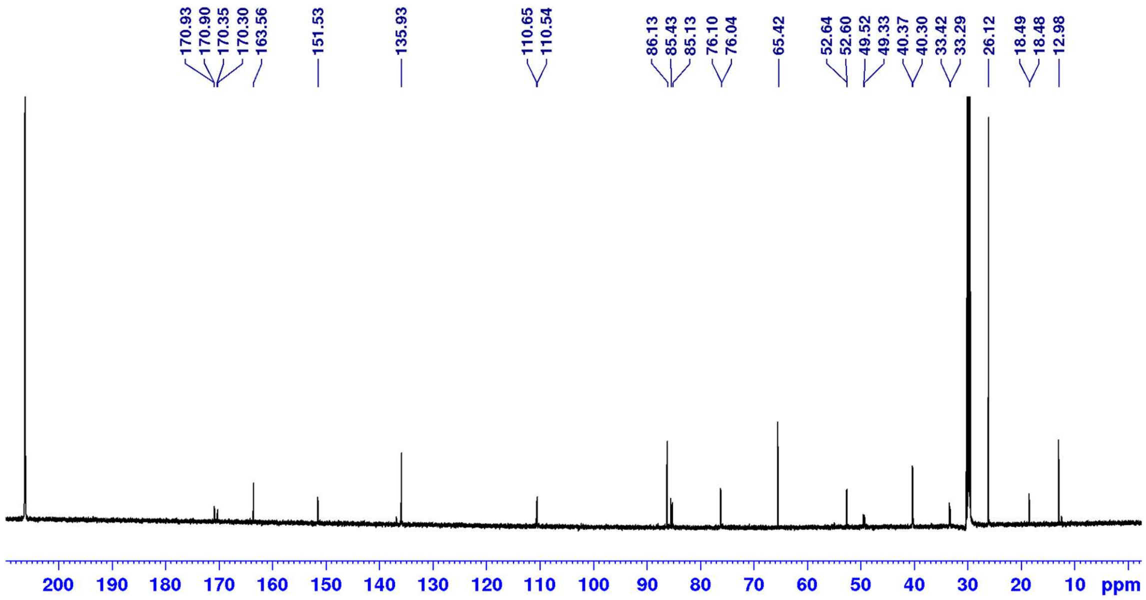

3′-Amino-3′-deoxy-5′-*O*-(4,4′-dimethoxytrityl)thymidine (31):

^{1}H NMR (400 MHz, acetone-d$_6$)

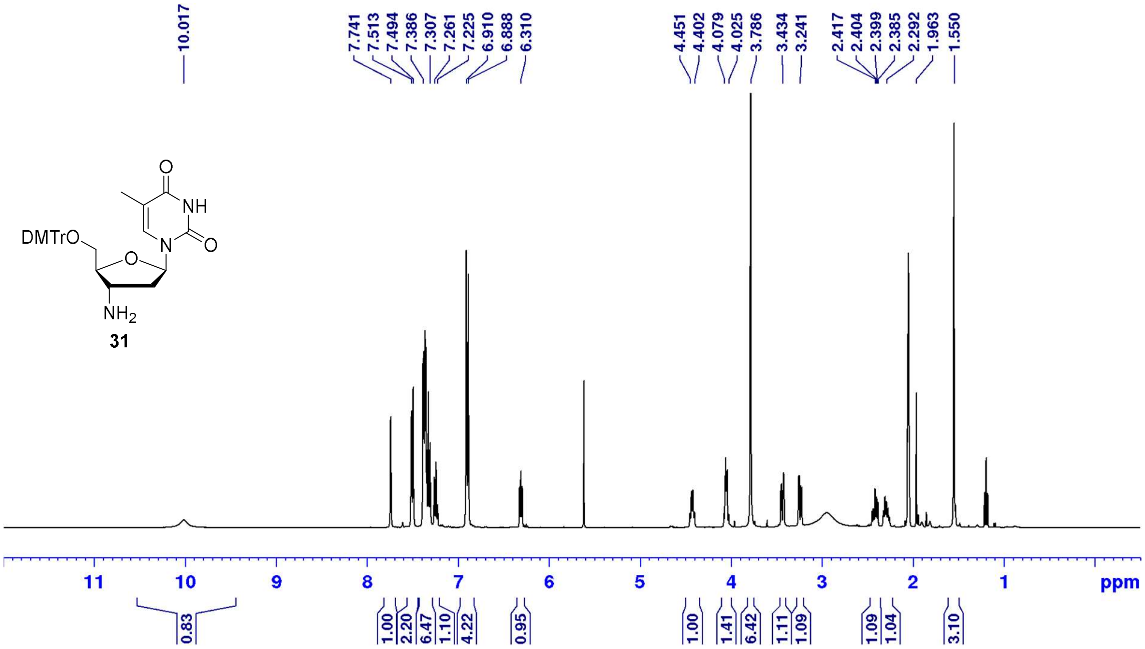

^{13}C NMR (151 MHz, acetone-d$_6$)

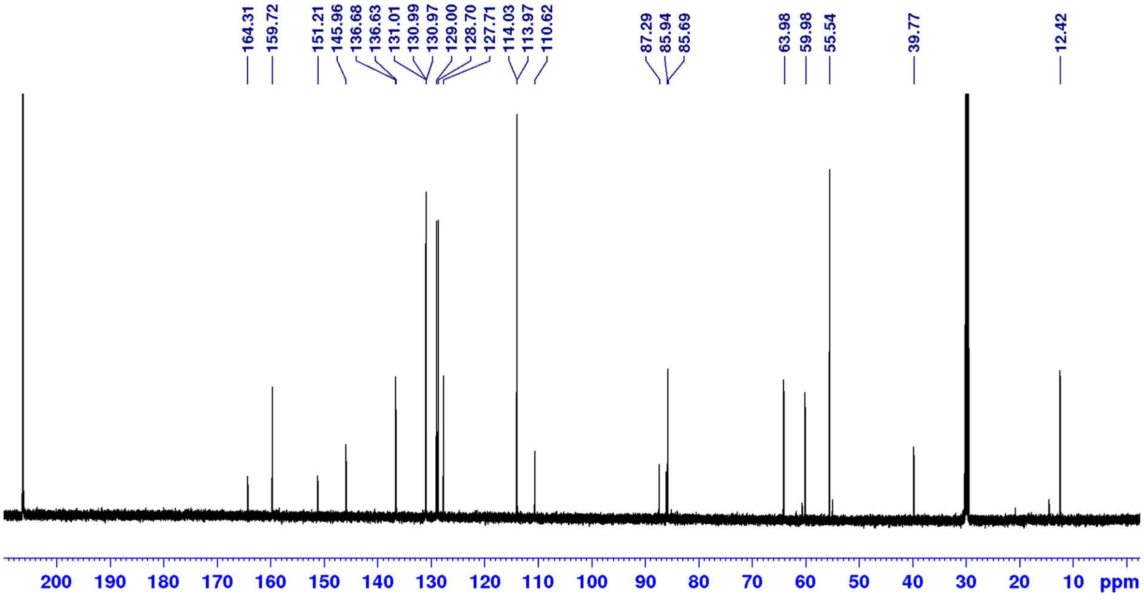

_C_-(3′-_O_-(_tert_-Butyldimethylsilyl)-5′-deoxythymidin-5′-yl)-_N_-(3′-deoxy-5′-_O_-(4,4′-dimethoxytrityl)thymidin-3′-yl) amido methyl malonate (32):

^{1}H NMR (600 MHz, acetone-d$_6$)

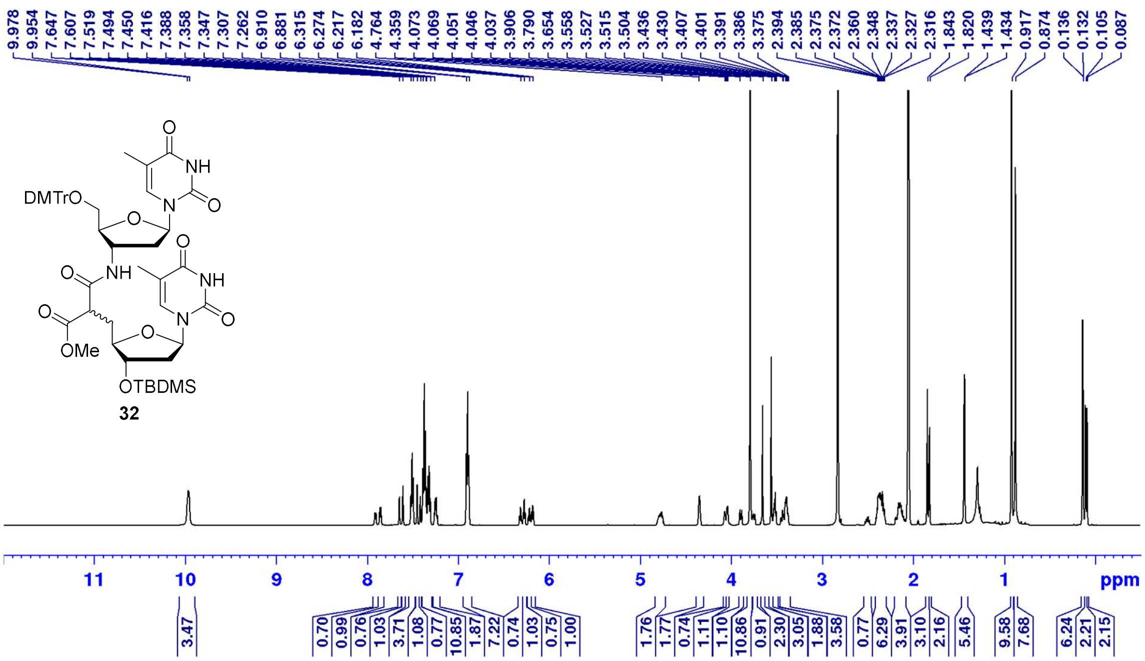

^{13}C NMR (151 MHz, acetone-d$_6$)

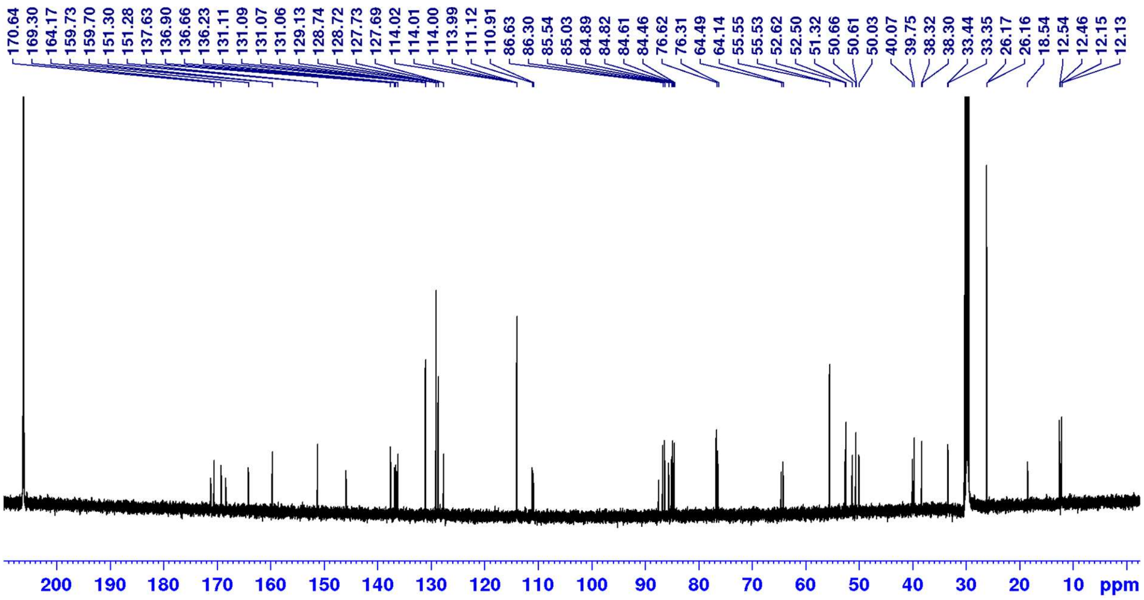

_N_-(3′-Deoxy-5′-_O_-(4,4′-dimethoxytrityl)thymidin-3′-yl)-_C_-(5′-deoxythymidin-5′-yl) amido methyl malonate (33):

^{1}H NMR (600 MHz, acetone-d$_6$)

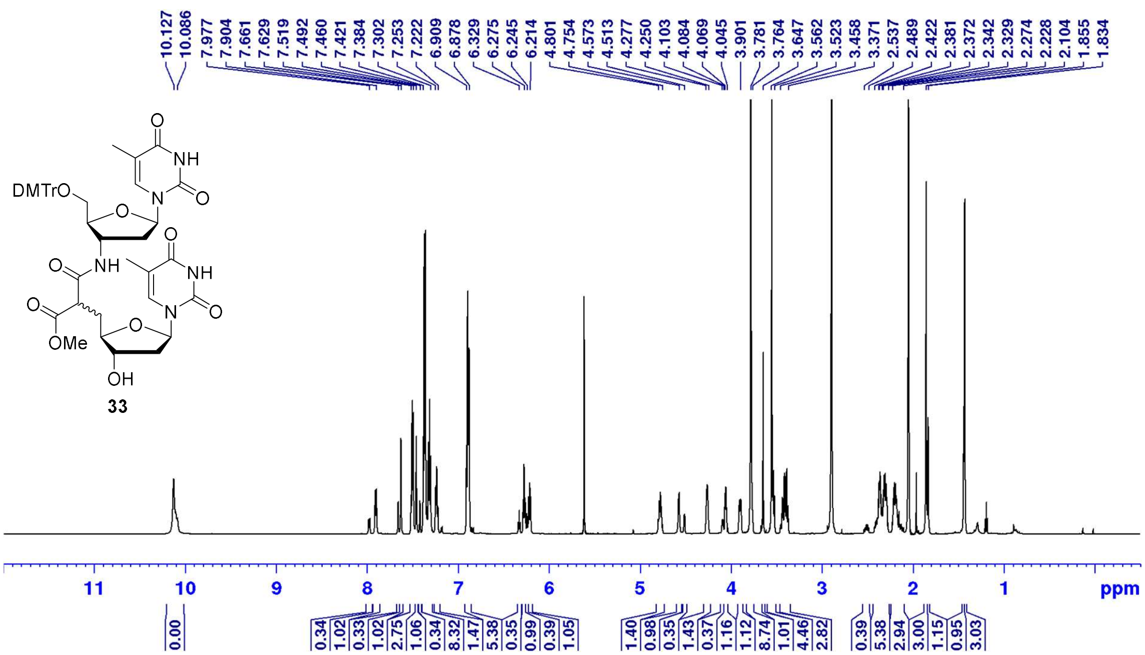

^{13}C NMR (151 MHz, acetone-d$_6$)

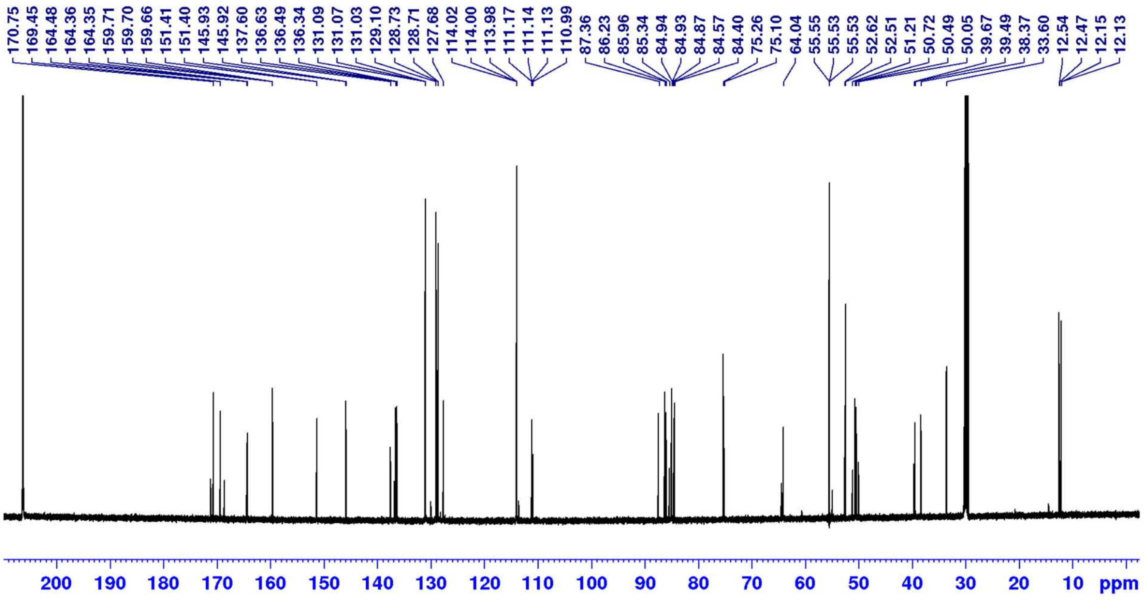

N-(3′-Deoxythymidin-3′-yl)-C-(5′-deoxythymidin-5′-yl) amido methyl malonate (34):

^{1}H NMR (400 MHz, DMSO-d$_6$)

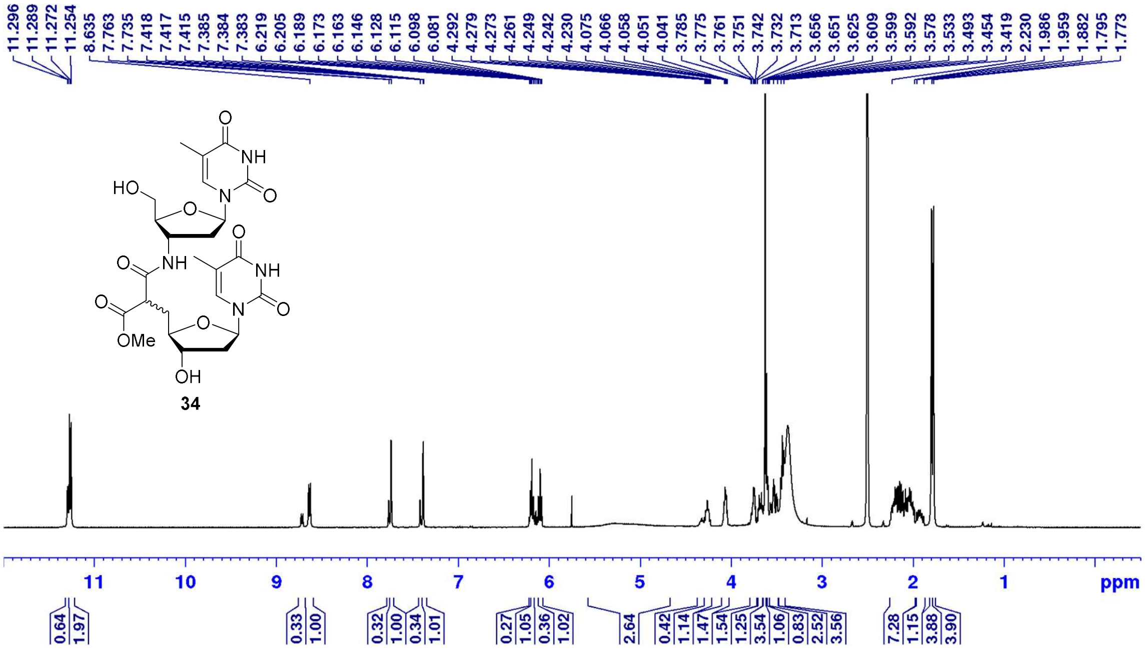

^{13}C NMR (100 MHz, DMSO-d$_6$)

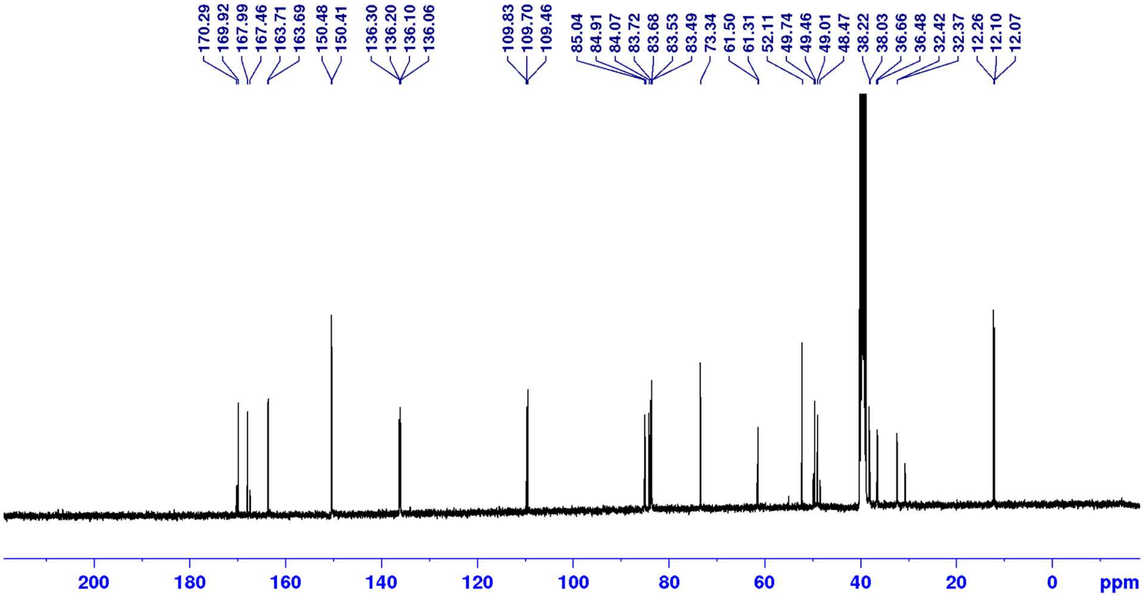

N-(3′-Deoxythymidin-3′-yl)-C-(5′-deoxythymidin-5′-yl) amido sodium malonate (35):

^{1}H NMR (600 MHz, CD_3OD)

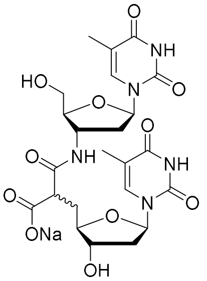

35

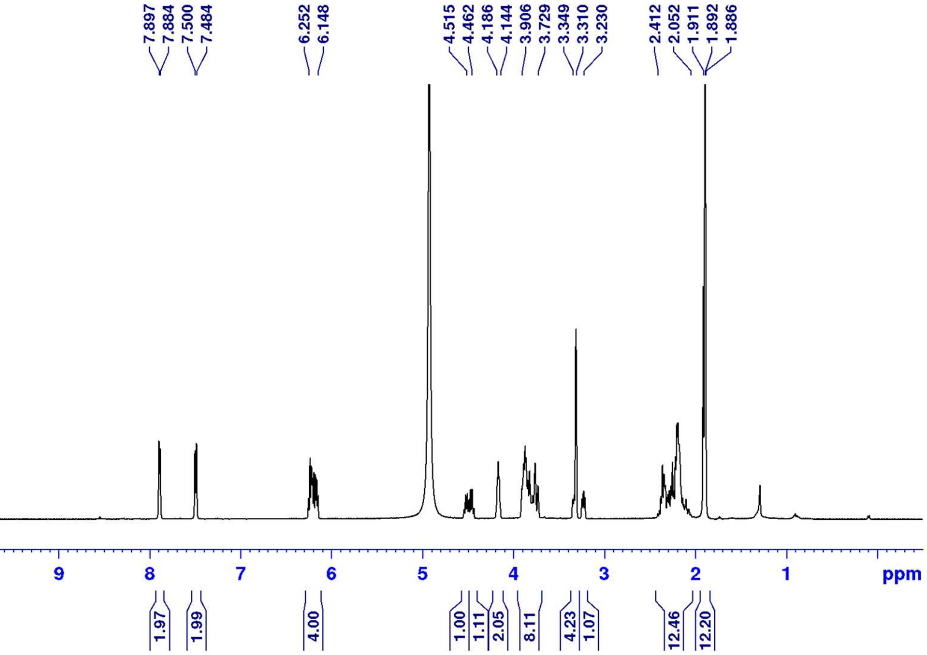

^{13}C NMR (151 MHz, CD_3OD)

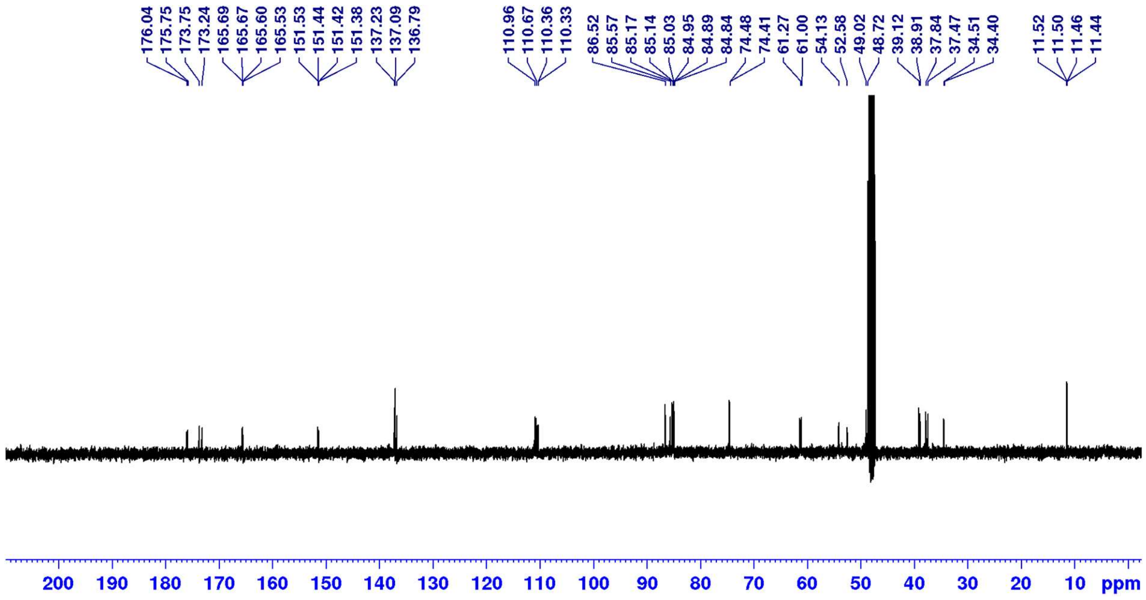

_N_-(3′-Deoxythymidin-3′-yl)-_C_-(5′-deoxythymidin-5′-yl) amido _N_-hydroxy malonamide (36):

^{1}H NMR (600 MHz, DMSO-d$_6$)

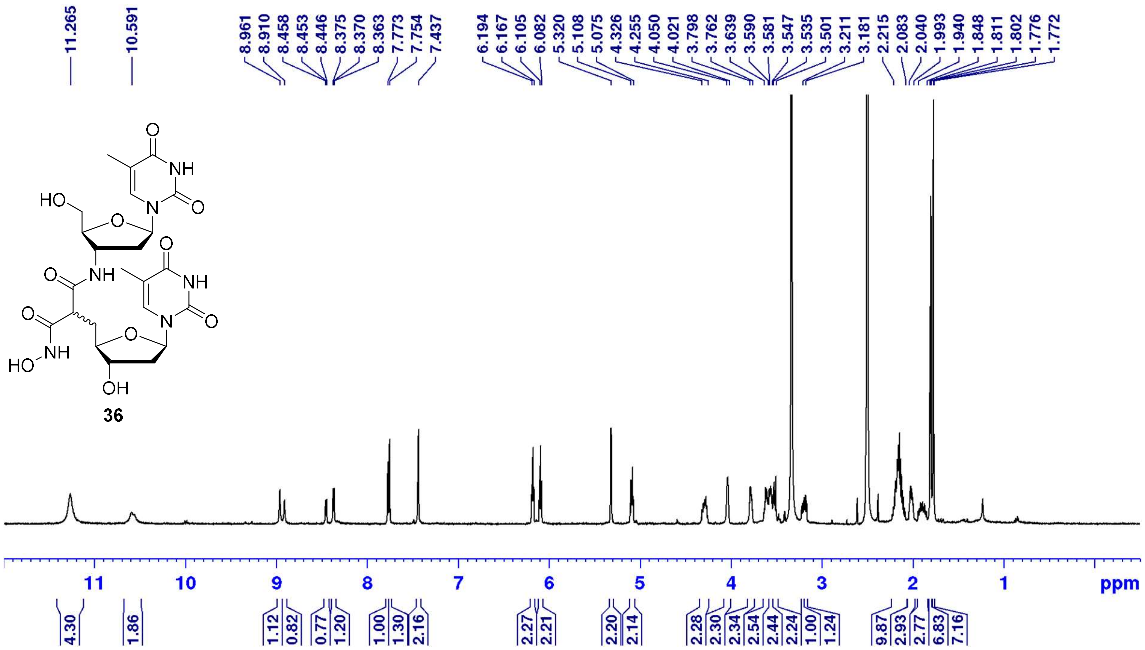

^{13}C NMR (151 MHz, DMSO-d$_6$)

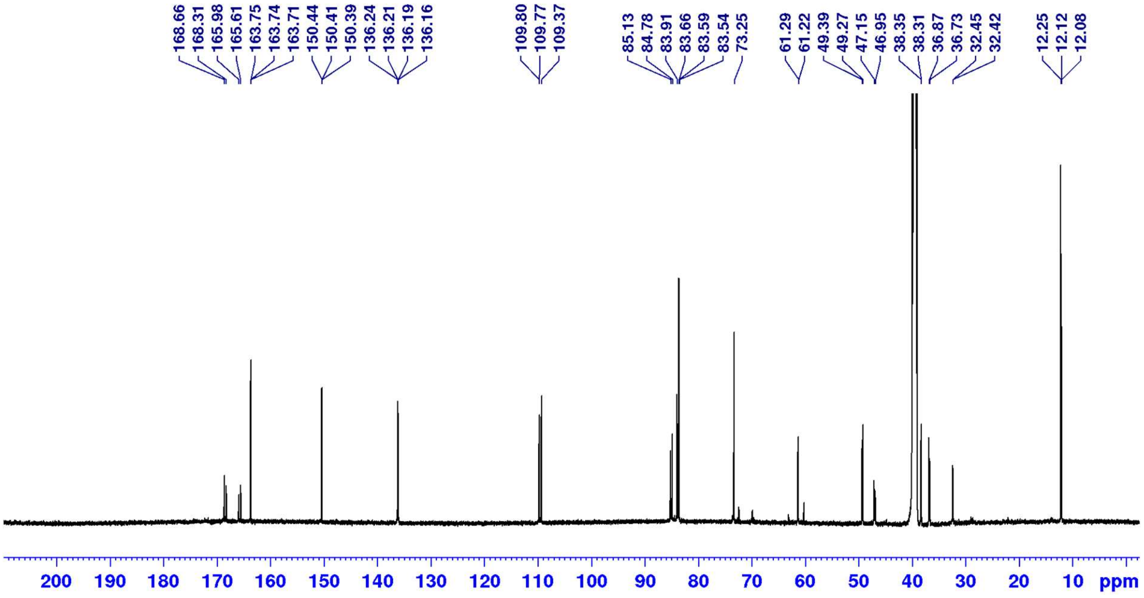

Supplement: Supplementary file 1 [file molecules-26-00320-s001.pdf]
